# Mannose‐Glycated Metal‐Phenolic Microcapsules Orchestrate Phenotype Switch of Macrophages for Boosting Tumor Immunotherapy

**DOI:** 10.1002/advs.202415565

**Published:** 2025-02-27

**Authors:** Xin Tan, Renwang Sheng, Weikun Li, Yinghua Tao, Zonghao Liu, Ning Yang, Syeda Safia Hashmi, Feiling Feng, Fangzhou Liu, Liqin Ge

**Affiliations:** ^1^ State Key Laboratory of Digital Medical Engineering School of Biological Science and Medical Engineering Southeast University Nanjing 210096 China; ^2^ School of Medicine Southeast University Nanjing 210009 China; ^3^ Department of Biliary Tract Surgery I Shanghai Eastern Hepatobiliary Surgery Hospital Navy Medical University Shanghai 200438 China; ^4^ Department of Head & Neck Surgery Jiangsu Cancer Hospital & Jiangsu Institute of Cancer Research & The Affiliated Cancer Hospital of Nanjing Medical University Nanjing 210029 China; ^5^ Advanced Ocean Institute of Southeast University Nantong 226000 China

**Keywords:** M1 macrophage polarization, macrophage‐related immunotherapy, mannose‐glycated bovine serum albumin, metal‐phenolic microcapsules

## Abstract

Microcapsules are advancing in immunotherapy, with both their core and shell being capable of loading immunoregulatory substances. Notably, microcapsules with intrinsic bioactivities can more directly modulate the immune microenvironment, while current research in this area remains scarce. Herein, immunomodulatory metal‐phenolic microcapsules (mMPMs) is developed through the one‐step assembly of dopamine‐modified hyaluronic acid (HADA) and Fe^III^ onto mannose‐glycated bovine serum albumin microbubbles (Man‐BSA MBs). Specifically, Man‐BSA formed during the early stages of the Maillard reaction is sonicated to produce microbubbles as templates for capsule preparation. Subsequently, HADA is rapidly coated on the templates and coordinates with Fe^III^ to form microcapsules after air escapes from MBs. Mass spectrometry analysis identifies abundant lysine glycation sites on Man‐BSA, with the highest glycation site percentage reaching 94.88%. Man‐BSA within mMPMs effectively promotes macrophage internalization, induces the accumulation of pro‐inflammatory mediators, and thereby results in the M1 polarization of macrophages, as further corroborated by proteomic analysis. Consequently, the compelling anti‐tumor effects of mMPMs are demonstrated both in vitro and in vivo. Overall, this work presents an immunomodulatory microcapsule that activates pro‐inflammatory phenotype macrophages, which is a promising microcarrier to improve immunotherapeutic effects.

## Graphical abstract

Immunomodulatory metal‐phenolic microcapsules (mMPMs) is developed in this study that leverages dopamine‐modified hyaluronic acid and Fe^III^ assemble on mannose‐glycated bovine serum albumin microbubbles. mMPMs effectively promote macrophage internalization and M1 polarization, enhancing pro‐inflammatory responses. Verified through proteomics and in vivo tests, mMPMs show significant anti‐tumor efficacy, offering a novel approach to boost immunotherapy.

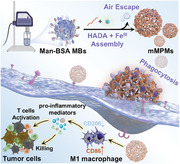

## 1 Introduction

Microcapsules have emerged as a promising drug delivery system due to their unique core‐shell structure, which allows for cargo loading or functional modification.^[^ ^1^, ^2^ ^]^ With advances in immunotherapy, antigens, drugs, immunostimulatory molecules, or adjuvants have been loaded into microcapsules to achieve high loading efficiency and controlled release for improved therapeutic effects.^[^ ^3^, ^4^ ^]^ Moreover, the microcapsule shell can be functionalized with immunopotentiators like cytosine–phosphate–guanosine and immunostimulatory lipids to synergistically activate immune cells.^[^ ^5^, ^6^ ^]^ As a critical component of the innate immune system, the macrophage is the primary line of host defense against many pathogens and diseases. Upon different stimuli, macrophages can be activated and polarized into classically activated pro‐inflammatory (M1) or alternatively activated anti‐inflammatory (M2) phenotypes to exert distinct biological functions and maintain tissue homeostasis. M2 macrophages predominantly produce high amounts of anti‐inflammatory cytokines, contributing to inflammation resolution, tissue repair, and inhibition of the tumor immuno‐microenvironment.^[^ ^7^, ^8^ ^]^ Conversely, M1 macrophages release reactive oxygen species (ROS), chemokines (e.g., CC chemokine ligand 2 and CXC chemokine ligand 2 (CXCL2)), and pro‐inflammatory cytokines (e.g., tumor necrosis factor α (TNF‐α), interleukin 1β (IL‐1β), and IL‐12), thus playing a critical role in antiviral, antitumoral, and antimicrobial processes.^[^ ^9^, ^10^, ^11^, ^12^ ^]^ In addition, M1 macrophage activation facilitates the subsequent activation of adaptive immune cells to perform immune tasks and reverse the immunosuppressive microenvironments of disease sites. Therefore, developing microcapsules that precisely induce macrophage polarization toward a pro‐inflammatory state is beneficial to enhance macrophage‐based immunotherapies. Current microcapsule‐based strategies to modulate M1 macrophage polarization include (i) loading pro‐inflammatory substances, such as proteins with modulatory properties in inflammation; (ii) utilizing β‐glucans and chitin from natural yeast microcapsule walls; (iii) delivering oxygen via microcapsules to alleviate hypoxia conditions.^[^ ^13^, ^14^, ^15^ ^]^ However, synthetic microcapsules with the intrinsic ability to induce M1 polarization of macrophages have rarely been reported.

It has been found that glycated proteins can induce M1 polarization of macrophages by activating related signaling pathways, such as the nuclear factor kappa‐B (NF‐κB) and mitogen‐activated protein kinase (MAPK) pathways.^[^ ^16^, ^17^, ^18^ ^]^ As a promising method to synthesize glycated proteins, the Maillard reaction, a non‐enzymatic reaction between the free amino group of a protein and the carbonyl group of reducing sugar, is a natural and spontaneous process that requires no additional chemicals and can be performed under controlled and safe conditions.^[^ ^19^, ^20^, ^21^ ^]^ D‐mannose (a natural C‐2 epimer of glucose, hereinafter referred to as “mannose”) is a reducing sugar that plays a crucial role in immune modulation upon recognition and uptake by immune cells via C‐type lectin receptors.^[^ ^22^ ^]^ Thus, mannose‐glycated proteins are expected to exhibit high affinity and immunomodulatory effects on macrophages. Specifically, existing studies have shown that glycation, particularly lysine glycation can alter protein conformation and consequently induce the expression of pro‐inflammatory cytokines, such as TNF‐α and IL‐1β.^[^ ^23^, ^24^ ^]^ Our previous studies demonstrated the one‐step generation of functional microcapsules through the assembly of metal–phenolic networks on bovine serum albumin microbubbles (BSA MBs).^[^ ^25^ ^]^ We hypothesize that mannose‐glycated BSA (Man‐BSA) could not only serve as a template for microbubble preparation but also remain on the resulting capsules to induce macrophage polarization, thereby endowing the microcapsules with intrinsic immunoregulatory properties.

**Scheme 1:**
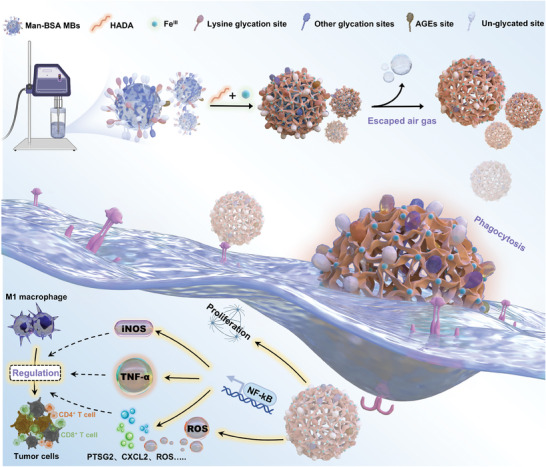
Preparation schematic of mMPMs and the mechanisms of mMPMs‐mediated M1 macrophage polarization and regulation of tumor cell death.

In this study, a one‐step assembly of engineered metal‐phenolic networks on Man‐BSA MBs was developed for the rapid construction of microcapsules with the intrinsic ability to induce M1 macrophage polarization (**Scheme** **1**). First, Man‐BSA for microbubble production was prepared by optimizing Maillard reaction conditions. The specific glycation sites and levels were precisely analyzed by mass spectrometry (MS). Subsequently, the dopamine‐modified hyaluronic acid (HADA) and Fe^III^ were coated onto the surface of microbubbles and crosslinked through hydrophobic and coordinated interactions. After the spontaneous escape of air from MBs, mannose‐glycated metal‐phenolic microcapsules (mMPMs) were successfully prepared. The remaining Man‐BSA on capsules exerted multiple effects on macrophages, including enhancing the internalization of mMPMs and activating oxidative stress. More specifically, the interactions between mMPMs and macrophages, as well as their effects on macrophage behaviors and functions, were comprehensively evaluated by global proteomics analysis. Furthermore, mMPMs‐mediated M1 polarization was validated at both transcriptional and protein levels. Finally, the anti‐tumor efficacy of mMPMs was assessed, demonstrating their potential as a microcarrier platform to enhance macrophage‐based tumor immunotherapies.

## 2 Results and Discussion

### 2.1 Preparation of Mannose‐Glycated BSA via the Early Stage of the Maillard Reaction

**Figure 1:**
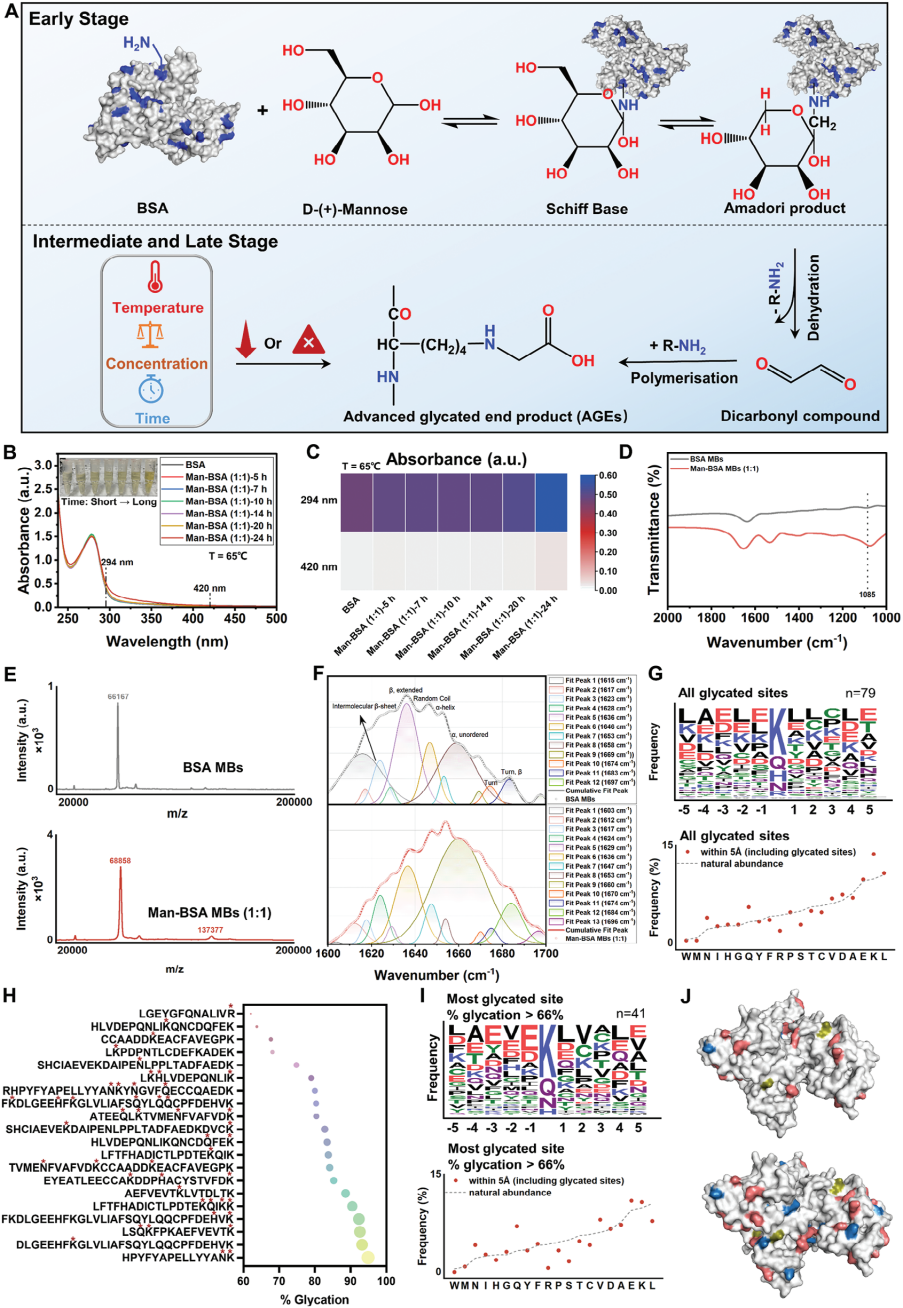
Preparation of mannose‐glycated BSA via the early stage of the Maillard reaction. A) A schematic representation of the early, intermediate, and late stages of the Maillard reaction between mannose and BSA, highlighting three key parameters influencing the reaction rate: temperature, time, and concentration. B) UV‐vis spectra of Man‐BSA (1:1) obtained at different heating times at 65 °C. The inset shows the macrographs of corresponding solutions. C) Heatmap illustrating the absorbance values at 294 and 420 nm for BSA and Man‐BSA (1:1) at different heating times at 65 °C. D) FTIR spectra of BSA MBs and Man‐BSA MBs (1:1). E) MALDI‐TOF mass spectrometry analysis of BSA MBs and Man‐BSA MBs (1:1). F) Curve‐fitting results of the FTIR spectra for BSA MBs and Man‐BSA MBs (1:1) in the region of the amide I band (1600–1700 cm⁻¹). G) Frequency logo and percentage of flanking residues surrounding all glycated sites identified in Man‐BSA MBs (1:1). H) The quantitative analysis of the top 20 sites exhibiting the highest levels of glycation in Man‐BSA MBs (1:1). The glycation sites are marked by the symbol *. I) Frequency logo and percentage of flanking residues surrounding most glycated sites (% glycation >66%) identified in Man‐BSA MBs (1:1). J) Surface diagram of native BSA (upper) and glycated BSA (below) [Protein Data Bank (PDB) ID: 4F5S]. The structure of BSA is shown in white, where lysine glycation sites and other glycation sites are colored red and blue, respectively. AGEs sites are indicated in brown.

Our previous study demonstrated that when BSA microbubbles served as templates for capsule preparation, BSA was still present in the interior of the formed capsule after the air escaped from the microbubble.^[^ ^25^ ^]^ In addition, it was reported that glycated products prepared by Maillard reaction of proteins and reducing sugars had immunomodulatory activity.^[^ ^16^, ^18^ ^]^ Thus, we expected that the mannose‐glycated BSA could remain in capsules and endow capsules with immunoregulatory ability. In the Maillard reaction between BSA and mannose, temperature, time, and concentration should be precisely controlled to obtain a higher yield of intermediate products (e.g., Amadori products) and to minimize the formation of advanced glycation end‐products (AGEs) (**Figure** **1**A). These AGEs will lead to the loss of protein value, the formation of potentially toxic substances, and age‐related diseases, and therefore should be inhibited.^[^ ^20^ ^]^ The presence of intermediate products in the Maillard reaction can be evaluated by measuring absorbance at 294 nm, while the formation of AGEs can be assessed by absorbance at 420 nm. To preliminarily determine the conditions of the Maillard reaction, solutions containing mannose and BSA in various mass ratios were heated at 70 °C (close to the denaturation temperature of BSA) for 30 min. It was found that different concentrations of mannose had no significant influence on the production of intermediate products and AGEs in a short time, as indicated by the similar absorbance at 294 and 420 nm (Figure S1A,B, Supporting Information). This implied that only a small proportion of mannose was attached to the protein, which was possibly caused by insufficient reaction time. Surprisingly, we observed that high concentrations of mannose can stabilize the protein structure near BSA's denaturation temperature and reduce the extent of heat‐induced cross‐linking, which facilitated the binding of mannose to BSA under prolonged reaction conditions (Figure S1C, Supporting Information).^[^ ^26^ ^]^ To avoid protein cross‐linking and further investigate the appropriate reaction time, the solution with a mannose‐to‐BSA mass ratio of 1:1 was chosen to explore the effect of different reaction times on glycated products. The results showed that the prolonged reaction time at this temperature led to the formation of more AGEs (Figure S1D,E, Supporting Information). Slight browning began to appear after 7 h, indicating that higher temperatures and longer heating times accelerated the Maillard reaction, thereby leading to a faster formation of AGEs.^[^ ^21^ ^]^ Related research has reported that 3 min of sonication can increase the solution temperature by 5 °C and that a temperature range between 60 and 65 °C was beneficial for obtaining the highest number of microbubbles in the solution.^[^ ^27^, ^28^ ^]^ Consequently, with the mannose‐to‐BSA ratio maintained at 1:1 and the reaction time unchanged, a reaction temperature of 65 °C (5 °C below the denaturation temperature of BSA) was selected. The result showed that as the reaction time increased, there was no significant formation of brown substances at 65 °C (Figure 1B,C). A slight increase in absorbance at 420 nm was observed only after a reaction time of 24 h, suggesting that the optimal reaction conditions could be set at 65 °C for 20 h for further investigation of the reaction ratio. The fluorescence emission spectra can reflect the binding situation of mannose and BSA. It was found that the fluorescence intensity at 340 nm decreased proportionally with an increasing mannose ratio, indicating that increased mannose‐binding to BSA led to the quenching of fluorescence from tryptophan residues in BSA (Figure S2A, Supporting Information). Fourier transform infrared (FTIR) spectroscopy analysis further revealed greater absorption in glycated BSA compared to native BSA near 1085 cm^−1^ (‐OH groups), which indicated the presence of extra saccharide groups in Man‐BSA (Figure 1D; Figure S2B, Supporting Information). Matrix‐assisted laser desorption/ionization time‐of‐flight (MALDI‐TOF) mass spectrometry accurately measured the molecular weight (MW) of glycated BSA with different mannose ratios. The results revealed that Man‐BSA with higher MW was obtained when the ratio of mannose increased, indicating that more mannose was incorporated into the BSA molecule (Figure 1E; Figure S2C, Supporting Information). These results strongly indicated that a higher proportion of mannose was beneficial for achieving a higher degree of glycation in BSA. However, a decreased foaming activity of Man‐BSA solution (2:1) was observed, which hindered the subsequent preparation of MBs and capsules (Figure S2D, Supporting Information). Consequently, the final parameters for manufacturing mannose‐glycated BSA were determined to be the reaction temperature of 65 °C, mannose‐to‐BSA ratio of 1:1, and heating time of 20 h. By comparing the molecular weight shift from native BSA to the corresponding glycated BSA, the incorporation ratio (IR) of the obtained Man‐BSA MBs (1:1) was 16.1, suggesting that each BSA molecule was attached to ≈16 mannose molecules (Figure 1E). Through secondary structure analysis of the prepared Man‐BSA MBs, we further discovered a proportional increase in glycation degree with the increase of α‐unordered and β‐turn structure. These findings were consistent with the previous reports that glycation sites tended to occur in β‐turn regions (Figure 1F; Figure S2E, Table S1, Supporting Information).^[^ ^29^ ^]^

To fully reveal the detailed glycation sites of Man‐BSA MBs used for capsule preparation, the MS and MS/MS spectra of Man‐BSA MBs (1:1) were analyzed. All potential glycation sites of Man‐BSA MBs were detected and displayed in Figure S3A (Supporting Information). Compared to native BSA MBs, Man‐BSA MBs prepared through the Maillard reaction and subsequent sonication exhibited 79 glycation sites (Figure S3B, Supporting Information). These included mono‐glycation sites as well as dual‐glycation sites that were not present in the original BSA MBs, along with sites where both mono‐ and dual‐glycations coexisted. This data was further used to generate frequency logos and percentages illustrating the five residues located on each side of the glycated site (Figure 1G). We observed that lysine (K) residues were most susceptible to glycation, followed by glutamine (Q), histidine (H), and asparagine (N) residues. Analysis of the chemical structures revealed that the ε‐amino groups of K residues and the imidazolyl groups of H residues exhibited high reactivity with reducing sugars, particularly the ε‐amino groups of K residues (Figure S3C,D, Supporting Information). Both Q and N residues, containing two amino groups, also showed relatively high reactivity (Figure S3E,F, Supporting Information). Frequency percentage analysis indicated that leucine and glutamic acid residues frequently occurred in the vicinity of glycation sites, suggesting that these sites were often surrounded by an acidic and hydrophobic environment. Subsequently, we performed quantitative analysis on all detectable glycation sites (Table S2, Supporting Information). Given the large number of glycation sites and variations in the degree of glycation for each site, we highlighted the top 20 sites with the highest glycation levels, which were likely to be closely related to the function of Man‐BSA (Figure 1H). Most of these highly‐glycated sites contained lysine residues, with the highest percentage of glycated sites reaching 94.88%. Figure 1I further demonstrated the frequent occurrence of K in the set of “most‐glycated” sequences, suggesting that these lysine glycation sites played a crucial role in the biological functions of Man‐BSA. Additionally, the enrichment of acidic residues (E) around “most‐glycated” sites was observed (Figure 1I). Next, representative AGEs were identified from the original MS and MS/MS data. Compared to native BSA MBs, the Man‐BSA MBs group exhibited only three additional AGEs sites (A) (Figure S3A,B, Supporting Information). Furthermore, quantitative analysis revealed that the percentage of newly added AGEs sites was less than 1%, indicating that the Maillard reaction had not progressed to the late stage (Figure S4A, Supporting Information). Surface electrostatic potential mapping showed that AGEs sites were more likely to occur in regions with higher positive charges (Figure S4B, Supporting Information). Finally, through the comprehensive analysis of the mass spectrometry data, the glycation sites of the prepared Man‐BSA were clearly identified. Except for glycated sites (represented in blue), such as Q, H, N, arginine, tryptophan, and a small number of AGEs (shown in brown), Man‐BSA had large amounts of lysine glycation sites (indicated in red), with the highest glycation site percentage of 94.88% (Figure 1J). Lysine glycation sites have been found to promote the production of inflammatory factors, such as TNF‐α and IL‐1β, suggesting that we can use the mannose‐modified BSA MBs as templates and further construct immunoregulatory capsules.^[^ ^23^ ^]^

### 2.2 Synthesis of HADA and Fabrication of mMPMs

**Figure 2:**
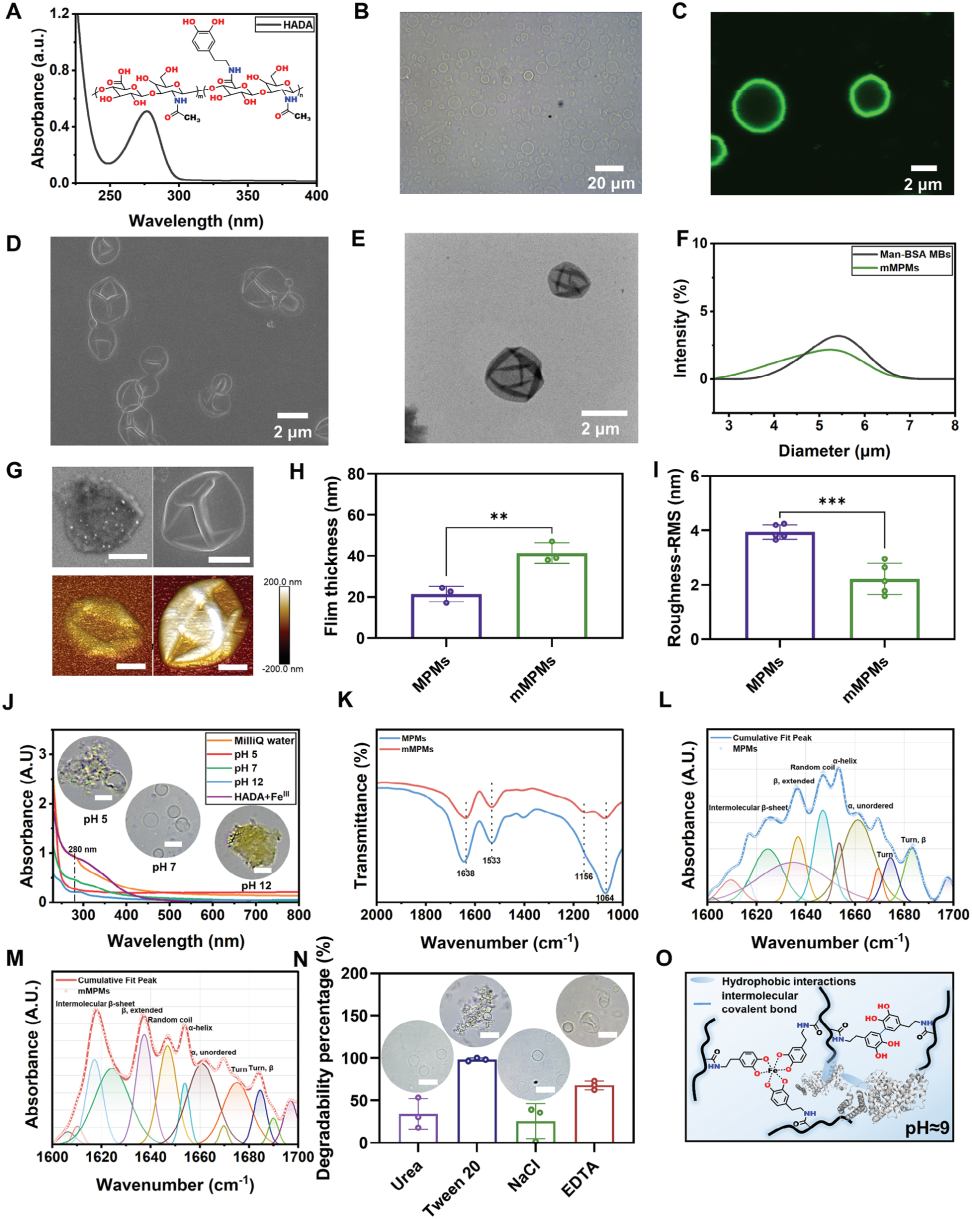
Synthesis of HADA and fabrication of mMPMs. A) UV‐vis spectra and chemical structure of the synthesized HADA. B) The optical image, C) CLSM image, D) SEM image, and E) TEM image of mMPMs. F) size distribution of Man‐BSA MBs and mMPMs. G) SEM and AFM images of MPMs and mMPMs (left: MPMs; right: mMPMs). The scale bars are 1 µm. H) Thickness (*n* = 3 capsules) and I) surface roughness (*n* = 5 capsules) of MPMs and mMPMs. J) UV‐vis spectra of HADA‐Fe^III^ complex aqueous solution, mMPMs in MilliQ water, and in solutions with different pH (*n* = 3). The insets are representative optical images of the corresponding capsules at various pH values. Scale bars are 10 µm. K) The FTIR spectra of MPMs and mMPMs. L) Curve‐fitted FTIR spectra focusing on the amide I band region (1600–1700 cm⁻¹) of MPMs. M) Curve‐fitted FTIR spectra highlighting the amide I band region (1600–1700 cm⁻¹) of mMPMs. N) Degradability percentage of mMPMs in the presence of 100 mm of urea, Tween 20, NaCl, or EDTA as assessed by counting chamber (hemocytometer) (*n* = 3 samples). The insets are the representative optical images of the corresponding capsules after incubation with the indicated solution. Scale bars are 10 µm. O) The dominant stabilized interactions in mMPMs, including hydrophobic (blue oval), coordination (black dotted line), and intermolecular covalent interactions (blue solid line). The results are shown as the mean ± SD. **(*p* < 0.01) and ***(*p* < 0.001) determined using a two‐tailed Student's *t*‐test H and I).

To construct immunomodulatory microcapsules, we selected Man‐BSA MBs as templates and hyaluronic acid (HA) as the shell material to enhance the affinity of the fabricated mMPMs with macrophages. HA, a molecule with excellent biocompatibility, is a major component of the extracellular matrix that can promote cell proliferation and migration.^[^ ^30^ ^]^ Therefore, we hypothesized that the HA‐based metal‐phenolic capsules would improve biocompatibility and promote cell growth. To assemble HA onto the capsules, HADA was synthesized by conjugating the carboxylic acid groups of HA (400 kDa) and the amine groups of dopamine using N‐(3‐(dimethylamino)propyl)‐N′‐ethylcarbodiimide (EDC) and N‐hydroxysuccinimide (NHS) (Figure S5A, Supporting Information), enabling the subsequent one‐step generation of HA‐based metal‐phenolic microcapsules through the coordinated interaction between dopamine and Fe^III^. The presence of a catechol absorption peak at 280 nm in UV‐vis spectrophotometry confirmed the successful preparation of HADA (**Figure** **2**A). Additionally, distinct peaks at δ 6.95–6.60 ppm and δ 1.8–2.0 ppm in the ^1^H NMR spectrum corresponded to the aromatic protons of grafted catechol moieties and the methylene group of HA, respectively. This further suggested the successful conjugation of dopamine to HA. By calculating the integral area ratio of the two peaks or using a standard curve established through UV‐vis spectroscopy, the ratio of dopamine conjugated to HA was determined to be ≈3.46% (Figure S5B–D, Supporting Information). Next, mMPMs were formed via the one‐step assembly of HADA and Fe^III^ onto Man‐BSA MBs, accompanied by the spontaneous escape of air from MBs during the centrifugation process (Scheme 1; Figure S6, Supporting Information). Optical microscopy, confocal laser scanning microscopy (CLSM), scanning electron microscopy (SEM), and transmission electron microscopy (TEM) images of mMPMs demonstrated that large quantities of capsules were successfully fabricated (Figure 2B–E). From Man‐BSA MBs to the formation of corresponding mMPMs, the zeta potential remained negative, with a slight decrease observed (Figure S7, Supporting Information). Additionally, the average size decreased from 5.43 ± 1.45 to 5.26 ± 2.04 µm accompanied by an increased polydispersity index, which was originated from the dynamic changes of Man‐BSA microbubbles during the assembly process (Figure 2F). High‐angle annular darkfield (HAADF) microscopy and energy‐dispersive X‐ray (EDX) spectroscopy mapping showed the presence of elements C, O, N, and Fe within the capsules (Figure S8, Supporting Information). The atomic force microscopy (AFM) images directly reflected the surface morphology of mMPMs. In comparison to the capsules templated by BSA MBs (metal‐phenolic microcapsules, MPMs, Figure S9, Supporting Information), mMPMs showed an increased thickness and a smoother surface, attributing to the integration of mannose into the shell of the capsules (Figure 2G–I). The UV‐vis absorption spectrum indicated that the capsules exhibited a wide UV‐vis absorbance band within the range of 300–400 nm, aligning with the characteristics of the HADA‐Fe^III^ complex sample. The pH‐dependent disassembly characteristics of mMPMs were also demonstrated using UV‐vis absorption spectra. Under pH 5 conditions, the capsules contracted and coalesced, while at pH 12, the capsules were notably disrupted, resulting in the disappearance of peaks at 300–400 nm (Figure 2J). FTIR spectrum provided deep insights into the presence of functional groups on mMPMs. The peaks observed at 1638 and 1533 cm^−1^ corresponded to the characteristic bands of amide I and amide II from BSA, respectively, indicating the incorporation of BSA within the capsules. The vibrational signals detected at 1064 cm^−1^ can be ascribed to the ‐OH groups originating from the synthesized HADA. Moreover, the distinct peak observed at 1156 cm^−1^ in the mMPMs correlated with the saccharide structure of mannose, which was not detected in MPMs (Figure 2K).^[^ ^31^ ^]^ Analysis of secondary structural conformations of BSA within the capsules (amide I band) further revealed a higher proportion of intermolecular β‐sheet, turn, and β‐turn structures in the mMPMs, consistent with the results observed in Man‐BSA MBs (Figure 2L,M; Table S3, Supporting Information). In addition, Tables S1 and S3 (Supporting Information) demonstrated a notable increase in the fraction of β‐extended structures in Man‐BSA after assembly of HADA and Fe^III^ compared to Man‐BSA MBs. This observation was consistent with our previous research, indicating that the secondary structure of Man‐BSA around the air core became more flexible and more exposed to the surrounding solution located on the Man‐BSA.^[^ ^25^ ^]^ The conformational changes in Man‐BSA following its assembly with HADA and Fe^III^ led to a decrease in the shell resistance of the Man‐BSA MBs. This, in turn, facilitated the release of air gases from the MBs, contributing to the formation of mMPMs. Subsequently, dominant forces that stabilized the mMPMs were investigated. Capsules with the same number were individually added to urea, Tween 20, NaCl, and ethylenediaminetetraacetic acid (EDTA) solution and incubated for 2 days. The remaining number of capsules was then monitored. The results revealed that only a small number of capsules remained after incubation in Tween 20. Optical microscopy images showed that all mMPMs were aggregated together (Figure 2N). Additionally, 67.85% of the capsules underwent disassembly in EDTA, as indicated by the wrinkled morphology of the capsules. These results suggested that hydrophobic and coordinated interactions were the dominant interactions for the stability of the mMPMs. Conversely, urea and NaCl solutions showed limited dissociation impact on the capsules, implying that hydrogen bonding and electrostatic interactions might not be the primary stabilizing forces for the mMPMs. The UV‐vis spectra further indicated that the catechol absorption peak at 280 nm and additional peaks at ≈350 nm that arose from catechol‐catechol or amino‐quinone bond formation, disappeared in the presence of Tween 20 and EDTA.^[^ ^32^ ^]^ However, these characteristic peaks remained in solutions of NaCl, urea, or PBS at pH 7, showing the good stability of mMPMs under physiological conditions (Figure S10, Supporting Information; Figure 2J). Therefore, we have confirmed that mMPMs were stabilized mainly through hydrophobic, coordination, and intermolecular covalent interactions, as indicated in Figure 2O.

### 2.3 The Interaction of mMPMs with Macrophages and Their Intrinsic Bioactivities in Regulating Macrophage Polarization

**Figure 3:**
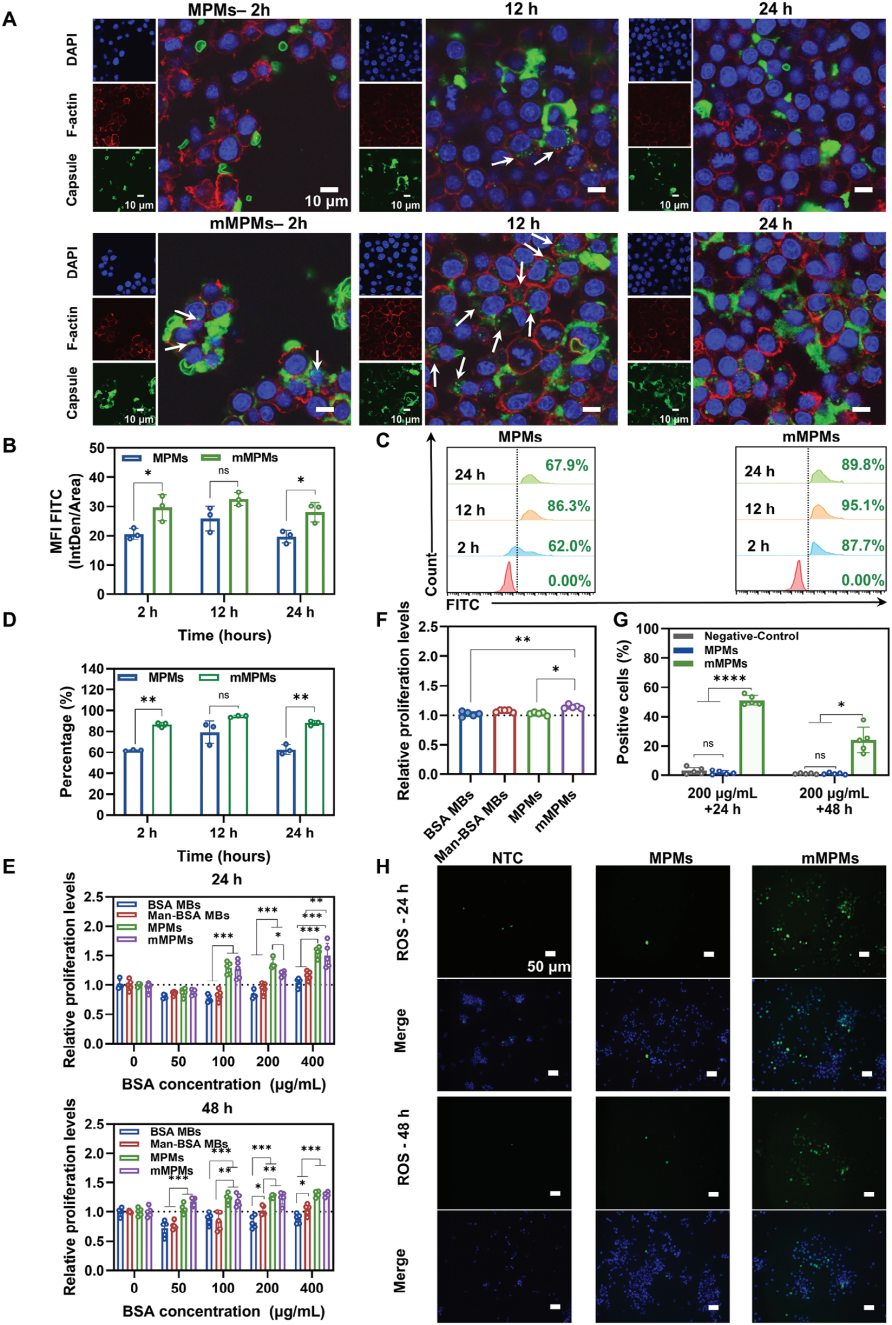
The interaction of mMPMs and macrophages in vitro. A) Confocal microscopy images of RAW 264.7 cells following exposure to MPMs or mMPMs for 2, 12, and 24 h. The microcapsules are stained with FITC (green), while cell nuclei and cytoskeleton are visualized in blue (DAPI) and red (Actin‐Tracker Red), respectively. White arrows indicate the capsules internalized by the cells. B) Intracellular mean fluorescence intensity (MFI) derived from 3 independent experiments (*n* = 3 independent experimental units (EUs)). C) Flow cytometry assessment of RAW 264.7 cells treated with 200 µg mL^−1^ MPMs and mMPMs for 2, 12, and 24 h. D) Quantitative evaluation of the flow cytometry data (*n* = 3 independent EUs). E) Relative cell proliferation levels of RAW 264.7 cells treated with BSA MBs, Man‐BSA MBs, MPMs, or mMPMs at the concentration of 0 to 400 µg mL^−1^ for 24 or 48 h (*n* = 5 independent EUs). F) Relative cell proliferation levels of RAW 264.7 cells treated with BSA MBs, Man‐BSA MBs, MPMs, or mMPMs at the concentration of 200 µg mL^−1^ for 72 h (*n* = 5 independent EUs). G) Quantitative analysis of ROS staining in macrophages (*n* = 5 independent EUs). H) Representative images of ROS staining in macrophages treated with MPMs or mMPMs at the concentration of 200 µg mL^−1^ for 24 or 48 h. The untreated cell was set as a NTC. The results are shown as the mean ± SD. ns (*p* > 0.05); *(*p* < 0.05); **(*p* < 0.01); ***(*p* < 0.001); and ****(*p* < 0.0001) determined using the Student's *t*‐test B and D), one‐way ANOVA with Tukey's post hoc test E and G) and Kruskal‐Wallis test F).

To explore the biological effects of mMPMs on macrophages, we first investigated the interactions of these microcapsules with macrophages. MPMs and mMPMs were labeled with fluorescein isothiocyanate (FITC). After incubation with these capsules for various durations, RAW 264.7 cells were stained with DAPI and Actin‐Tracker Red for confocal imaging analysis. As shown in **Figure** **3**A, a strong green fluorescence intensity was observed around macrophages in the mMPMs group at 2 h, significantly higher than that in the MPMs group. When the incubation time increased to 12 h, more fluorescence signals were detected within cells in mMPMs group, as indicated by white arrows. As time increased, the fluorescent signals inside the cells of both groups gradually decayed, which may be attributed to the normal digestive activity of macrophages to exogenous substances.^[^ ^33^ ^]^ The quantitative analysis revealed that the mean fluorescence intensity (MFI) in the mMPMs group was ≈1.5‐fold higher than that in the MPMs group at 2 h, and remained at a high level until 12 h (Figure 3B). Similar results were also obtained by flow cytometry (Figure 3C,D). In comparison to the MPMs group (62%) at 2 h, the percentage of FITC‐positive macrophages in the mMPMs group increased to 87.7%. At 12 h, more than 95% of capsules in the mMPMs group had entered the cells. Subsequently, FITC‐positive macrophages in both groups gradually decreased, which was consistent with the confocal microscopy results. Given the proliferative characteristics of RAW 264.7 cells, primary bone marrow‐derived macrophages (BMDMs) were utilized as a model to more accurately investigate the interactions between mMPMs and macrophages. This approach provided a closer representation of in vivo conditions. The results demonstrated that BMDMs interacted more rapidly with mMPMs at 2 h, evidenced by increased overlapping green and red fluorescence (indicated by white arrows in Figure S11, Supporting Information). In contrast, many capsules in the MPMs group remained extracellular at this time point (indicated by gray arrows). At 12 h, green fluorescence was detected within the cytoplasm of both groups, indicating that the capsules had been fully internalized by the BMDMs. At 24 h, the intensity of green fluorescence within cells decreased, likely due to the normal digestion of foreign substances by macrophages, similar to observations with RAW 264.7 cells.^[^ ^33^ ^]^ Collectively, these findings were similar to observations with RAW 264.7 cells, indicating that the internalization of mMPMs by macrophages was significantly enhanced compared to that of MPMs. Furthermore, we investigated the uptake of mMPMs by tumor cells and fibroblasts. As shown in Figure S12 (Supporting Information), after co‐incubation of MPMs and mMPMs with mouse breast cancer (4T1) cells or mouse fibroblast (L929) cells for 2 h, similar levels of green fluorescence were detected intracellularly. No significant difference in intracellular MFI was observed between groups, indicating that unlike macrophages, tumor cells and stromal cells exhibited no apparent differences in their uptake capabilities toward MPMs and mMPMs.

**Figure 4:**
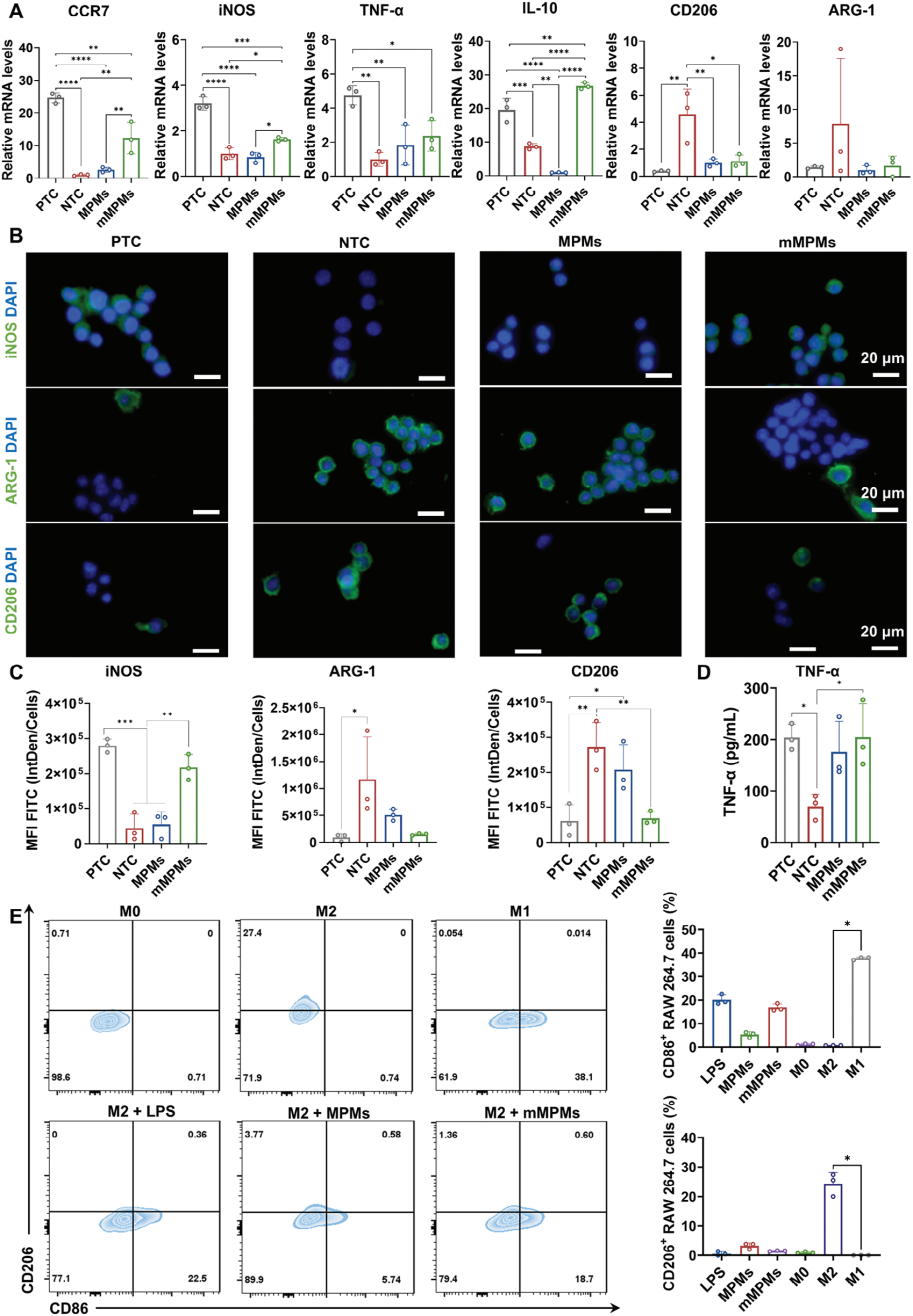
Biological effects of mMPMs on macrophages in vitro. A) Relative gene expressions of *CCR7*, *iNOS*, *TNF‐α*, *IL‐10*, *CD206*, and *ARG‐1* in RAW 264.7 cells treated with 200 µg mL^−1^ MPMs or mMPMs for 48 h (*n* = 3 independent EUs). B) Representative images of immunofluorescence staining for iNOS, ARG‐1, and CD206 in RAW 264.7 cells after LPS, MPMs or mMPMs treatment for 48 h. Scale bars = 20 µm. C) Quantitative analysis of immunofluorescence staining for iNOS, ARG‐1, and CD206 (*n* = 3 independent EUs). D) Expression levels of TNF‐α in RAW 264.7 cells treated with LPS, MPMs or mMPMs analyzed by ELISA (*n* = 3 independent EUs). The untreated cell was set as a NTC, and the LPS‐stimulated one was set as a PTC. E) Representative flow cytometry analysis and corresponding quantification of the number of M1 (CD86^+^) and M2 (CD206^+^) macrophages after different treatments in vitro (*n* = 3 independent EUs). The results are shown as the mean ± SD. *(*p* < 0.05); **(*p* < 0.01); ***(*p* < 0.001); and ****(*p* < 0.0001) determined using one‐way ANOVA with Tukey's post hoc test (A, C: iNOS and CD206, and D) and Kruskal‐Waillis test (A and C: ARG‐1; E).

Subsequently, we further elucidated the effect of the internalized microcapsules on macrophages. First, the proliferation and viability of macrophages under different treatments were evaluated using the cell counting kit‐8 (CCK8) assay after co‐incubation for 24 and 48 h. The results indicated that when the concentration was lower than 400 µg mL^−1^, the relative cell proliferation level in BSA MBs and Man‐BSA MBs groups was slightly less than 1 (Figure 3E). Thus, the live/dead staining experiment was carried out to further evaluate their potential cytotoxicity. As shown in Figure S13 (Supporting Information), large areas of green fluorescence were observed in all groups within the tested concentration range (50–400 µg mL^−1^). Similar to the control group, BSA MBs and Man‐BSA MBs groups did not cause significant cell death after incubation with RAW 264.7 cells for 24 h, as indicated by the low level of red fluorescence intensity. These results highlighted no significant cytotoxic effects of BSA and Man‐BSA MBs on macrophages in the range of concentrations used. Surprisingly, we found that high concentrations (exceeding 100 µg mL^−1^) of MPMs and mMPMs notably enhanced macrophage proliferation, with proliferation levels higher than control (Figure 3E). In addition, we observed that although MPMs exhibited higher relative proliferation levels after 24 h of co‐incubation, there was no significant proliferation difference between MPMs and mMPMs groups at 48 h. Building upon this observation, we further co‐cultured mMPMs and MPMs with RAW 264.7 cells for 3 days to validate the prolonged pro‐proliferative effects of mMPMs. As depicted in Figure 3F, the growth of cells was not inhibited in all groups. mMPMs exerted the greatest effect, significantly promoting cell proliferation. The above results demonstrated that after coating with HADA and Fe^III^, both mannose‐glycated and non‐glycated capsules could significantly boost macrophage proliferation. Especially, mMPMs exhibited a prolonged promotion of cell proliferation, which could be attributed to the Man‐BSA within the mMPMs, as evidenced by the slightly higher relative proliferation level of Man‐BSA MBs compared to BSA MBs. Next, DCFH‐DA, a reactive oxygen species assay kit, was used to examine the intracellular ROS production of RAW 264.7 cells, which initially reflected the activation and functions of macrophages.^[^ ^33^ ^]^ First, we observed the higher fluorescence from the ROS probe in the Man‐BSA MBs group rather than the BSA MBs group, suggesting that mannose‐glycated BSA may trigger ROS accumulation in macrophages (Figure S14, Supporting Information). To further validate, RAW 264.7 cells were treated with MPMs or mMPMs for 24 and 48 h, with untreated cells serving as the negative control (NTC). The results revealed that no obvious fluorescence was observed in the NTC and MPMs groups at 24 or 48 h, indicating the negligible effects of MPMs on ROS generation. On the contrary, the fluorescence images and quantitative analysis revealed a significantly higher percentage of ROS‐positive cells in the mMPMs group at 24 and 48 h compared to the NTC and MPMs groups, indicating that mMPMs promoted ROS accumulation in macrophages (Figure 3G,H). It has been widely reported that increased ROS production is involved in the metabolic reprogramming and M1 polarization of macrophages.^[^ ^34^ ^]^ Thus, we further investigated gene expression of M1 (*CCR7*, *iNOS*, and *TNF‐α*) and M2 (*CD206*, *ARG‐1*, and *IL‐10*) markers in macrophages treated with different microcapsules by qPCR. RAW 264.7 cells stimulated with LPS were used as a positive control (PTC) for M1 polarization, while untreated ones served as the NTC. After treatment with MPMs, mMPMs, and LPS for 24 h, we first observed the morphological changes of macrophages under the bright field microscope (Figure S15, Supporting Information). Compared to the unpolarized spherical morphology in the NTC and MPMs groups, RAW 264.7 cells in mMPMs and PTC groups exhibited significant elongation and tentacles, suggesting the potential M1 polarization. After incubation for 48 h, qPCR results showed that LPS induced significantly increased expressions of *CCR7*, *iNOS*, and *TNF‐α* and substantially reduced *CD206* and *ARG‐1* expressions compared to the NTC (**Figure** **4**A), confirming M1 polarization of macrophages after LPS stimulation. The macrophages in the MPMs group showed significantly decreased expressions of *CD206* and *IL‐10* but similar expression levels of pro‐inflammatory genes including *CCR7*, *iNOS*, and *TNF‐α* compared to the NTC, suggesting its mild role in activating the pro‐inflammatory responses of macrophages (Figure 4A). In contrast, mMPMs substantially increased *CCR7*, *iNOS*, and *TNF‐α* expressions and inhibited *CD206* and *ARG‐1* expressions compared to the NTC, which was highly similar to the expression patterns of LPS‐induced M1 macrophages. Interestingly, IL‐10, a typical anti‐inflammatory cytokine, increased in macrophages treated with LPS and mMPMs stimulations, which could be attributed to a self‐regulating reaction of the cell to prevent excessive inflammation (Figure 4A).^[^ ^35^ ^]^ In addition, the macrophages in the mMPMs group had significantly higher expression levels of *iNOS*, a gene encoding the crucial enzyme for nitric oxide (NO) generation,^[^ ^34^ ^]^ compared to those in the NTC and MPMs groups, which probably resulted in the increased ROS accumulations in mMPMs‐treated macrophages (Figure 3H). Moreover, immunofluorescence staining and enzyme‐linked immunosorbent assay (ELISA) were used to verify the expression of M1 and M2 markers at the protein level. After 48 h of co‐culture, both LPS and mMPMs induced macrophages to polarize toward an M1 phenotype, as evidenced by a significant increase in iNOS‐positive macrophages and a marked decrease in ARG‐1 and CD206‐positive macrophages (Figure 4B,C). Similar to the NTC group, cells in the MPMs group did not exhibit significant expression of pro‐inflammatory markers. ELISA results further demonstrated that mMPMs stimulated macrophages to secrete more pro‐inflammatory factor TNF‐α, showing effects similar to PTC (Figure 4D). Taken together, these findings combined with qPCR results demonstrated that the glycation of MPMs enhanced their immunomodulatory properties, thus endowing mMPMs with the capacity to induce M1 polarization and activate pro‐inflammatory responses in macrophages. The tumor microenvironment typically contains a higher proportion of M2 macrophages, which are known to promote tumor growth and angiogenesis while contributing to an immunosuppressive milieu that hinders effective antitumor immune responses. The repolarization of M2‐like macrophages to the M1 phenotype can reverse the immunosuppressive microenvironment and enhance anti‐tumor immunity. ROS and inflammatory mediators have been reported to have the ability to repolarize the M2‐phenotype macrophages toward the M1 phenotype, thereby promoting a pro‐inflammatory and anti‐tumor immune response.^[^ ^36^ ^]^ To substantiate the immunotherapeutic potential of mMPMs, we first evaluated the capacity of mMPMs to induce M2‐to‐M1 repolarization of macrophage in vitro by flow cytometry. RAW 264.7 cells, when induced by cell culture medium, IL‐4 + IL‐13, and LPS, represent macrophages with M0, M2, and M1 phenotypes, respectively (Figure 4E). RAW 264.7 cells were induced to the M2 phenotype prior to further treatment. After incubation with LPS, MPMs, and mMPMs for 24 h, we found that LPS notably induced the expression of CD86 with a positive rate of 22.5%, showing M2‐to‐M1 repolarization of macrophages induced by LPS. Similar to the expression patterns of LPS‐induced M1 macrophage, 18.7% of the CD86^+^ cells in the mMPMs group were detected. In contrast, a substantial presence of CD86^+^ cells was not observed in the MPMs group, suggesting that MPMs lacked the capability to induce the repolarization of M2‐like macrophages toward an M1 phenotype. To provide more robust and relevant data, we further investigated the expression of M1 and M2 markers at both the gene and protein levels in BMDMs. First, we extracted bone marrow cells from mice and induced their differentiation into M2‐type BMDMs using 20% L929 cell supernatant over a period of 5 days. Optical microscopy images showed that on day 5, the cells had adhered and exhibited a spindle shape, indicating their M2 phenotype (Figure S16A, Supporting Information). Flow cytometry confirmed that the proportion of CD206^+^ cells was ≈55.4 ± 6.8% (Figure S16B, Supporting Information). After incubating M2‐type BMDMs with mMPMs and MPMs for 48 h, the gene expressions of M1 (*iNOS, IL‐1β, TNF‐α*) and M2 (*CD206*) were detected by qPCR. The results showed that mMPMs significantly upregulated the expression of pro‐inflammatory genes, including *iNOS*, *IL‐1β*, and *TNF‐α* while downregulating the expression of the anti‐inflammatory gene *CD206* (Figure S17A, Supporting Information). Specifically, the expression of *iNOS* was notably higher in the PTC and mMPMs groups compared to the NTC and MPMs groups. The mMPMs group showed a significant increase in *IL‐1β* expression, similar to the PTC group (LPS‐stimulated BMDMs). The expression of *CD206*, an anti‐inflammatory marker, was significantly lower in the mMPMs group compared to the NTC and MPMs groups, again resembling the pattern observed in the PTC group. These findings suggested that both mMPMs and LPS (PTC group) can induce a shift in BMDM polarization from an anti‐inflammatory phenotype to a pro‐inflammatory phenotype. Subsequently, the expression of iNOS at the protein level in BMDMs after different treatments was evaluated by immunofluorescence staining. The images demonstrated that no obvious fluorescence was observed around the nuclei in the NTC and MPMs groups, while similar green fluorescence intensity was detected in the PTC and mMPMs groups (Figure S17B,C, Supporting Information). These results confirmed the ability of mMPMs to induce M2‐to‐M1 repolarization of macrophages.

### 2.4 Proteomic Analysis to Uncover the mMPMs‐Triggered Cellular Responses and Underlying Mechanisms in Macrophages

**Figure 5:**
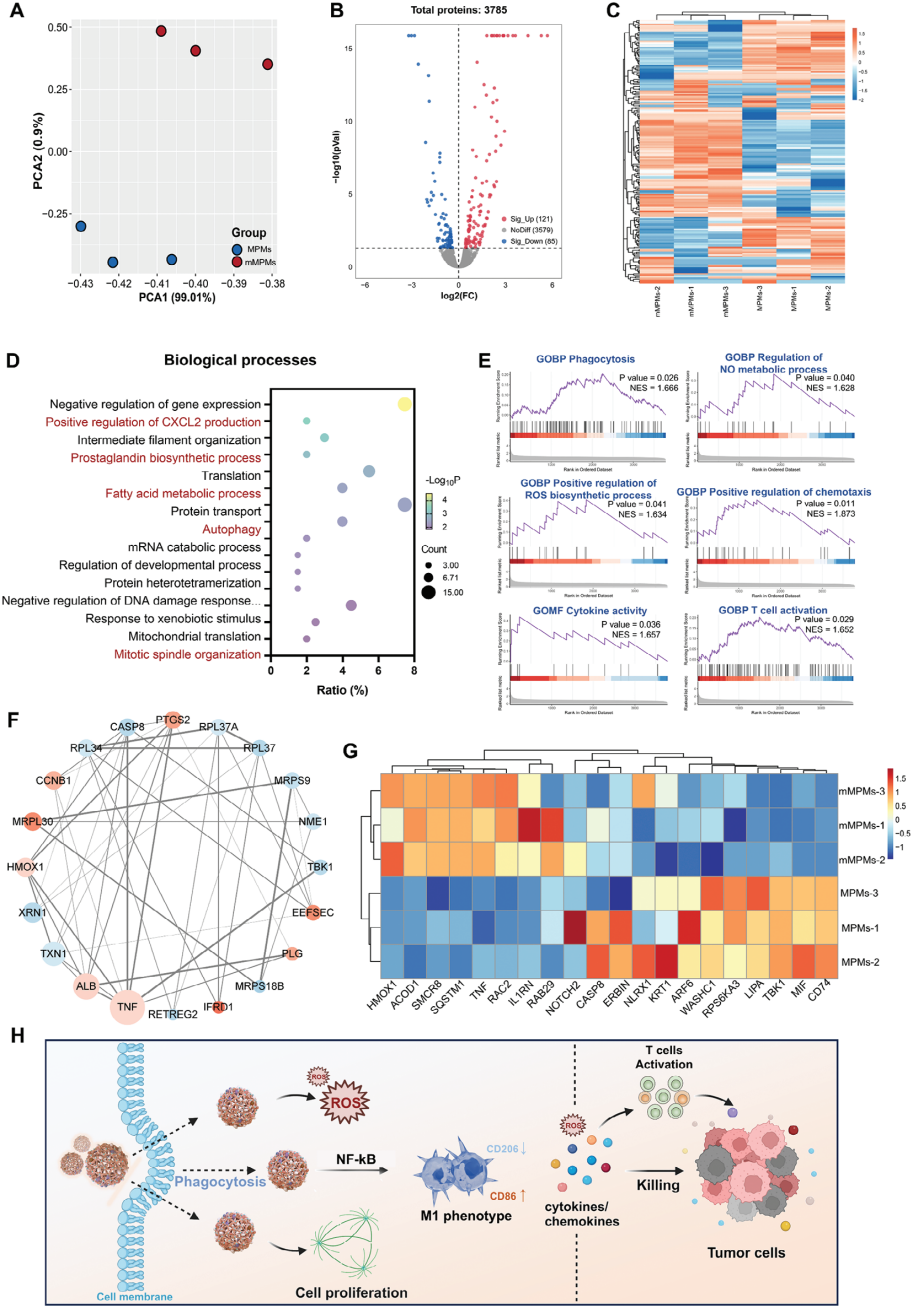
The altered cellular responses and its underlying mechanisms in macrophages treated with mMPMs via global proteomic analysis. A) PCA plot of proteomic data in the two groups (*n* = 3 independent EUs). B) Volcano plot of all protein expressions in MPMs versus mMPMs. C) Heatmap of all DEPs in two groups after cluster analysis. D) Top GO terms in BP significantly enriched from all DEPs (mMPMs vs MPMs). E) GSEA plots associated with phagocytosis, NO metabolic and ROS biosynthetic process, chemotaxis and cytokine, and T cell activation (mMPMs vs MPMs). F) Protein interaction network of the top 20 DEPs (mMPMs vs MPMs). G) Protein expression of immune response‐related proteins from proteomic profiles (mMPMs vs MPMs). H) The proposed mechanisms of mMPMs‐mediated macrophage M1 polarization and anti‐tumor growth. The diagram was created using BioRender (https://biorender.com/).

To validate the biological effects of mMPMs on macrophage polarization and elucidate their underlying mechanisms, proteomic analysis was performed to obtain a global view of the changed biological processes and signaling pathways in macrophages treated with un‐glycated or glycated capsules (MPMs or mMPMs). After incubating RAW 264.7 cells with MPMs or mMPMs for 3 d, cells were collected for label‐free quantification (LFQ)‐based proteomic analysis. As shown in **Figure** **5**A, principal component analysis (PCA) showed that the replicates of both groups were assembled into clear groups, indicating high reliability and repeatability of proteomic data. A total of 3785 proteins were identified in both groups (Figure 5B). When comparing the mMPMs group to the MPMs group, we recognized 206 differentially expressed proteins (DEPs, *p* < 0.05), including 121 up‐regulated and 85 down‐regulated (Figure 5B). The heatmap of all DEPs after cluster analysis exhibited notably different expression patterns in the two groups (Figure 5C).

Subsequently, to obtain functional insight into these changed proteins, all DEPs were used for Gene ontology (GO) and Kyoto Encyclopedia of Genes and Genomes enrichment analysis. Figure 5D showed the top GO terms in the biological process (BP), which were most likely influenced by mMPMs. The result revealed that various cellular responses associated with the generation of chemokines and pro‐inflammatory factors, autophagy, and cell proliferation were significantly changed, with specific to “positive regulation of CXCL2 production”, “prostaglandin biosynthetic process”, “fatty acid metabolic process”, “autophagy”, and “mitotic spindle organization”. Specifically, CXCL2 is a class of CXC chemokines that are strong chemoattractants for macrophages and also are inflammation stimulators.^[^ ^37^ ^]^ Similarly, prostaglandins are physiologically active lipid compounds with diverse hormone‐like effects, which are involved in inflammation‐related signaling.^[^ ^38^ ^]^ The fatty acid metabolic process may influence endoplasmic reticulum stress, which in turn affects inflammation in macrophages and drives M1 polarization.^[^ ^39^ ^]^ Autophagy is a stress‐induced process, such as increased ROS. It has been reported that autophagy can mediate macrophage polarization, and down‐regulation of autophagy promotes M1 polarization.^[^ ^40^ ^]^ These terms suggested that mMPMs induced macrophage polarization toward the M1 phenotype by regulating the above biological processes. In addition, the term “mitotic spindle organization” revealed that mMPMs can initiate cell proliferation, which was consistent with our previous observation in Figure 3F. To complement the limitations of GO analysis, gene set enrichment analysis (GSEA) analysis was performed to further elucidate the cellular changes mediated by mMPMs. Consistent with the result in Figure 3A–D, the phagocytosis of macrophages was significantly enhanced by mMPMs (Figure 5E). The results further revealed that “regulation of NO metabolic process” and “positive regulation of ROS biosynthetic process” were up‐regulated in the mMPMs group compared to the MPMs group, which could be contributed to the increased ROS generation and M1 polarization of macrophages after the internalization of mMPMs (Figures 3H and 4). In addition, it was observed that the mMPMs‐induced macrophages showed an up‐regulation of chemokines and cytokines, probably resulting in the production and secretion of more pro‐inflammatory factors. These pro‐inflammatory ROS, NO, cytokines, and chemokines not only enhanced the ability of macrophages to eliminate pathogens such as viruses and tumors but also effectively reshaped the immunosuppressive microenvironment at disease sites. Furthermore, we observed that the macrophages activated by mMPMs could further trigger subsequent adaptive immune responses of other immune cells, such as T cells (Figure 5E).

Then, we analyzed the interaction network of all recognized DEPs in mMPMs versus MPMs (Figure S18, Supporting Information) and screened the top 20 DEPs that possibly play more important roles in the changed cellular processes in macrophages (Figure 5F). Notably, TNF, a classical pro‐inflammatory cytokine, was significantly up‐regulated in the mMPMs group compared to the MPMs group, which was also observed in the transcriptional and protein levels in Figure 4A,D, confirming mMPMs‐stimulated M1 polarization of macrophage. TNF is rapidly released after trauma or infection and serves as one of the most abundant early mediators in inflammatory responses. Increased TNF expression enhances the ability of macrophages to eliminate tumors, viruses, and other pathogens.^[^ ^41^ ^]^ Heme Oxygenase 1 (HMOX1) is an important antioxidant enzyme that will be up‐regulated in the cells with enhanced oxidative stress.^[^ ^42^ ^]^ HMOX1 expression was significantly up‐regulated, which can be attributed to increased iNOS expression and ROS generation in macrophages treated with mMPMs, as evidenced by Figures 3H and 4A–C. Besides, the proteins responsible for pro‐inflammatory factor generation (PTGS2) and macrophage recruitment/activation (PLG and CCNB1) displayed increased expressions in macrophages after mMPMs stimulations. The significant expression of these pro‐inflammatory factors in the mMPMs group highlighted that mMPMs could activate macrophages and induce them to polarize to a pro‐inflammatory phenotype. Subsequently, we further elucidated the effect of mMPMs on macrophage activation and identified proteins associated with “Macrophage activation” (https://www.informatics.jax.org/vocab/gene_ontology/GO:0042116). TNF and group IVA phospholipase A_2_ (PLA2G4A) were the two most distinct proteins significantly up‐regulated in the mMPMs group (Figure S19, Supporting Information). Similar to TNF, PLA2G4A is considered a crucial regulatory factor, and variations in its expression levels may influence the polarization state of macrophages. Specifically, studies have documented a higher abundance of group IVA cytosolic phospholipase A2 (cPLA2α or PLA2G4A) in M1 macrophages, indicating that elevated PLA2G4A expression could potentially promote the polarization of pro‐inflammatory M1‐type macrophages.^[^ ^43^ ^]^

Numerous studies have shown that glycated proteins may activate downstream intracellular signaling pathways, such as NF‐κB and MAPK pathways (including c‐Jun N‐terminal kinase (JNK), extracellular signal‐regulated kinase (ERK), and p38).^[^ ^16^, ^17^, ^18^, ^44^, ^45^ ^]^ These downstream signaling pathways can mediate oxidative stress or increased inflammatory mediators, thereby regulating macrophage M1 polarization. To elucidate the specific signaling pathways, we assessed changes in NF‐κB and MAPK‐related protein levels in macrophages treated with MPMs and mMPMs. Western blotting was performed to investigate the contribution of MAPK family proteins ERK and JNK to mMPMs‐induced macrophage activation. The data demonstrated no significant differences in the levels of p‐ERK and p‐JNK after treatment with mMPMs or MPMs, indicating that M1 polarization of macrophages induced by mMPMs may not be mediated through MAPK pathways (Figure S20, Supporting Information). Subsequently, immunofluorescence staining was employed to further investigate NF‐κB activation in macrophages following treatment with mMPMs. After treatment with 200 µg mL^−1^ MPMs or mMPMs for 48 h, accumulation of NF‐κB in the nucleus was observed in macrophages with mMPMs exposure compared to the control and MPMs groups (Figure S21, Supporting Information). Quantitative analysis further revealed that the nuclear‐to‐cytoplasmic fluorescence ratio in the mMPMs group was ≈1.5‐fold higher than in the other two groups. Proteomic analysis combined with qPCR and ELISA results demonstrated that TNF‐α was significantly increased in macrophages after mMPMs treatment. NF‐κB is widely recognized as a key downstream effector in TNF signaling. These results confirmed that the NF‐κB signaling pathway in macrophages was activated following mMPMs stimulation, thereby promoting the production of inflammatory mediators and mediating M1 polarization. To further elucidate the impact of mMPMs on the immune responses of macrophages, we investigated the proteins associated with “Immune response” (https://www.informatics.jax.org/go/term/GO:0006955) and identified 20 proteins dysregulated (Figure 5G; Figure S18, Supporting Information). In addition to the upregulated pro‐inflammatory proteins, we identified several significantly down‐regulated proteins, such as CD74 and macrophage migration inhibitory factor (MIF). CD74 is a type II transmembrane glycoprotein that possesses several biological functions. A small fraction of CD74 displayed on the cell surface and served as a receptor for MIF. The MIF‐CD74 axis has been demonstrated to play a critical role in initiating oncogenic signaling pathways, thereby promoting tumor growth and inducing an immunosuppressive environment.^[^ ^46^ ^]^ Thus, the mMPMs‐induced M1 macrophages showed the potential to change the immunosuppressive environment of tumors and suppress tumor growth. In general, these results confirmed that mMPMs effectively induced macrophages polarized into the M1 phenotype, which exhibited increased expressions of pro‐inflammatory factors (TNF and PTGS2) and inhibited the MIF‐CD74 axis (Figure S22, Supporting Information).

Taken together, these findings provided a comprehensive understanding of the immunomodulatory effects of mMPMs on macrophage polarization. Specifically, the Man‐BSA on mMPMs promoted the phagocytosis of macrophages to mediate the rapid internalization of microcapsules. Then, the internalized mMPM stimulated the generation of intracellular ROS and pro‐inflammatory factors through the NF‐κB signaling pathway, resulting in the activation of M1‐type macrophages. These paracrine signals of mMPMs‐induced macrophages may play roles in activating T cells and suppressing the growth of tumor cells. Therefore, the mMPMs with intrinsic immunomodulatory properties are a potential candidate to enhance macrophage‐based immunotherapy for tumor treatment (Figure 5H).

### 2.5 Evaluation of the Anti‐Tumor Effect of mMPMs In Vitro and In Vivo

**Figure 6:**
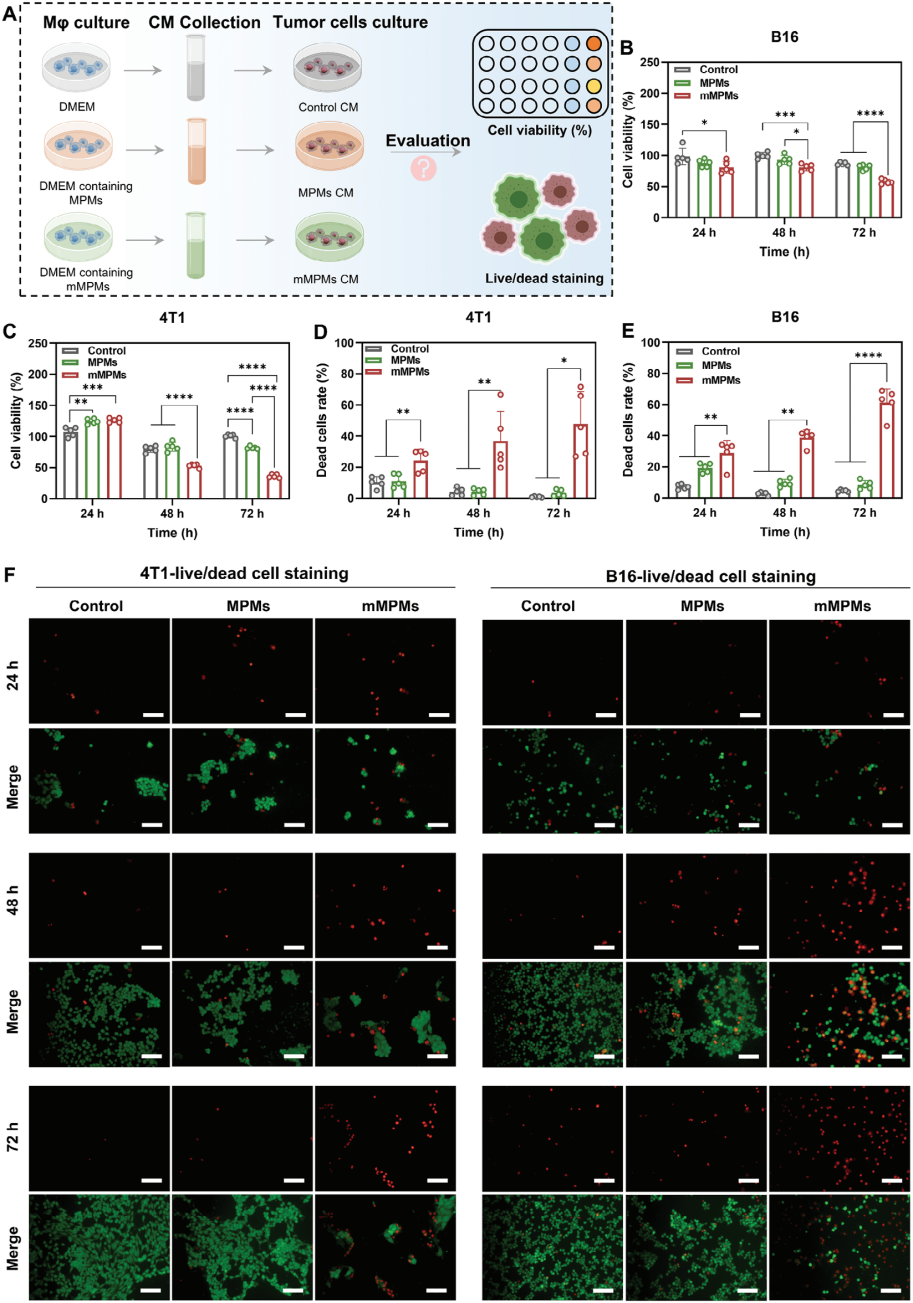
Evaluation of mMPMs‐mediated anti‐tumor effects in vitro. A) Illustrative diagram outlining the procedures for examining the impact of paracrine signals from mMPMs or MPMs‐activated macrophages on tumor cell proliferation. B) Cell viability of B16 cells treated with control and the paracrine signals from MPMs or mMPMs‐activated macrophage for 24, 48, and 72 h (*n* = 5 independent EUs). C) Cell viability of 4T1 cells treated with control and the paracrine signals from MPMs or mMPMs‐activated macrophage for 24, 48, and 72 h (*n* = 5 independent EUs). D) Quantification of dead 4T1 cells from live/dead staining images (*n* = 5 independent EUs). E) Quantification of dead B16 cells from live/dead staining images (*n* = 5 independent EUs). F) Representative live/dead staining images of 4T1 and B16 cells incubated with normal cell culture medium (control) and medium containing paracrine signals from MPMs and mMPMs‐activated macrophage for 24, 48, and 72 h. Scale bars are 100 µm. The results are shown as the mean ± SD. *(*p* < 0.05); **(*p* < 0.01); ***(*p* < 0.001); and ****(*p* < 0.0001) determined using one‐way ANOVA with Tukey's post hoc test B–E).

**Figure 7:**
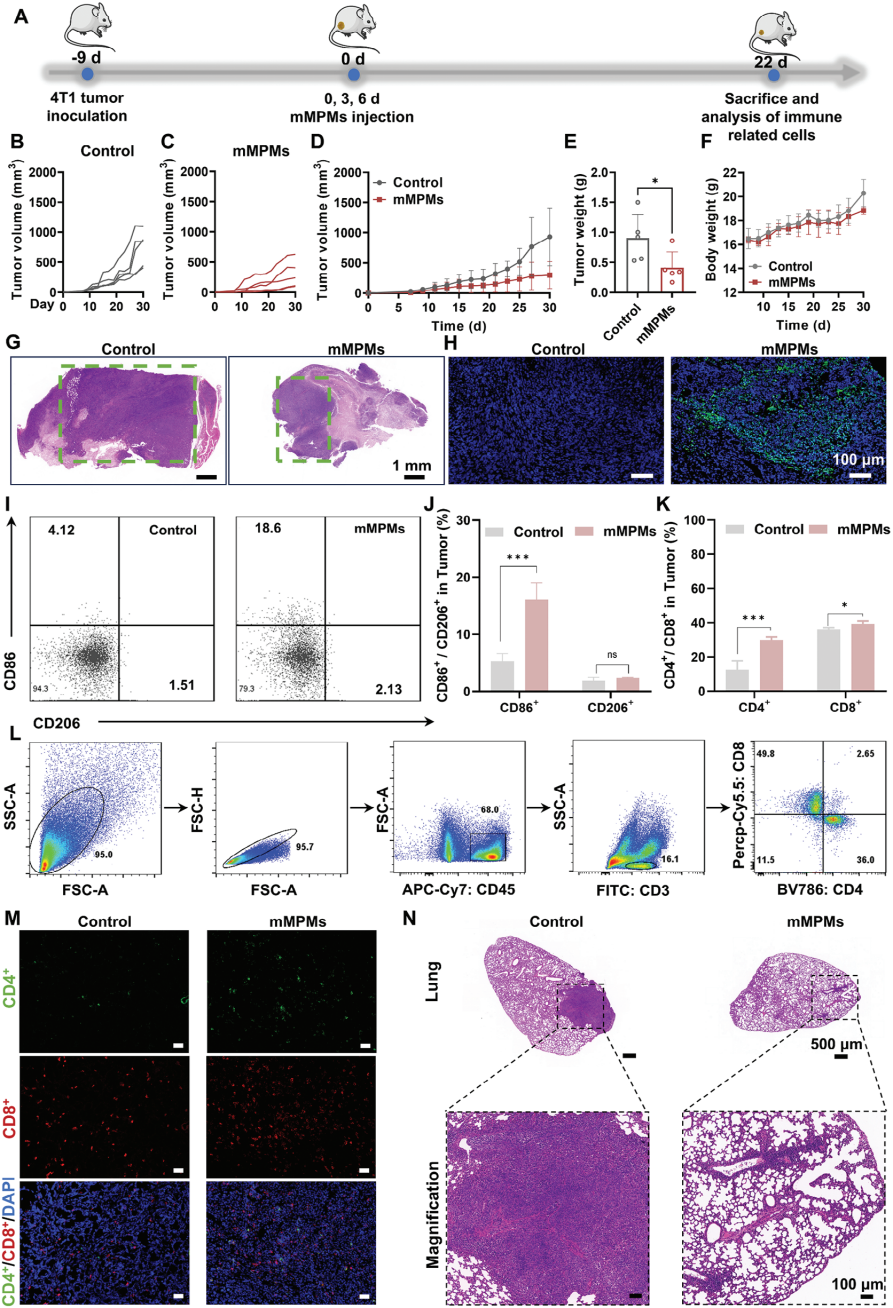
Evaluation of mMPMs‐mediated anti‐tumor effects in vivo. A) Illustration depicting the treatment protocol for 4T1 tumor‐bearing mice. B) The tumor growth curves of mice in the control group. C) The tumor growth curves of mice in mMPMs group. D) The average tumor growth curve for both groups of mice (*n* = 5 biological replicates). E) The tumor weight in control and mMPMs groups after treatment (*n* = 5 biological replicates). F) The body weight of mice in control and mMPMs groups during treatment (*n* = 3 biological replicates). G) H&E and H) TUNEL staining of tumor tissues from mMPMs and control groups at the end of treatment. I) Representative flow cytometry analysis and J) corresponding quantification of M1 (CD86^+^) and M2 (CD206^+^) macrophages in tumors (*n* = 3 biological replicates). K,L) The flow cytometric gating strategy and corresponding quantification of CD4^+^ and CD8^+^ T cells in tumors (*n* = 3 biological replicates). M) The representative images of the CD8^+^ (red) and CD4^+^ (green) immunofluorescence staining outcomes on tumor tissue sections from 4T1 tumor‐bearing mice sacrificed on day 22 post either PBS (control) or mMPMs treatment. Scale bars are 50 µm. N) H&E‐stained histological sections of lung tissue from the control and mMPMs groups. Enlarged images highlight areas of tumor metastasis. Scale bars of the top row and bottom row are 500 and 100 µm, respectively. The results are shown as the mean ± SD. ns (*p* > 0.05); *(*p* < 0.05); and ***(*p* < 0.001) determined using the two‐tailed Student's *t*‐test E, J, and K).

Based on the above results, we further evaluated the anti‐tumor effects of mMPMs in vitro and in vivo. For the cell experiments, we collected conditioned media (CMs) from macrophages treated with MPMs or mMPMs for 1 d, as illustrated in **Figure** **6**A. To remove any potential residual MPMs or mMPMs, CMs were filtered and further used for culturing different tumor cells, including 4T1 and mouse melanoma (B16) cells. CCK8 assay and live/dead staining were conducted to evaluate the effects of these macrophage paracrine signals on tumor cell growth. We found that the cell viability of B16 and 4T1 cells in the mMPMs group significantly decreased compared to the control and MPMs groups at different time points (Figure 6B,C). Specifically, after 72 h of incubation, the cell viabilities of B16 and 4T1 cells in the mMPMs group were 60.77 ± 3.74% and 35.16 ± 2.83%, respectively, indicating that the paracrine signals of mMPMs‐activated M1 macrophages effectively inhibited tumor cell growth. In contrast, MPMs showed a mild influence on the viability of tumor cells, especially for B16 cells (Figure 6B). The cell viabilities of 4T1 cells in the MPMs and mMPMs groups were slightly increased compared to the control groups at 24 h, which could be attributed to insufficient incubation time (Figure 6C). As incubation time prolonged, the growth of 4T1 cells was significantly inhibited by the secreted paracrine signals in mMPMs groups. We further clarified the effects of macrophage‐derived paracrine signals on killing tumor cells using live/dead staining. After being incubated with the corresponding CMs for 24, 48, and 72 h, B16 and 4T1 cells in the mMPMs group showed a substantially higher number of dead cells than those in the control and MPMs groups (Figure 6F). Quantitative analysis revealed that the percentage of dead cells for both B16 and 4T1 cells was below 20% in the control and MPMs groups at all time points, while that in the mMPMs group gradually increased with incubation time. At 72 h, the percentage of dead B16 and 4T1 cells in the mMPMs group reached 61.30 ± 8.93% and 45.71 ± 20.61%, respectively (Figure 6D,E). Collectively, these results demonstrated that the paracrine signals of mMPMs‐stimulated macrophage effectively inhibited the growth of different tumor cells. The therapeutic effect of mMPMs in vivo was further investigated. Our study focused on comparing control and mMPMs groups to highlight the comprehensive effects of mMPMs as a potential delivery system on tumor treatment and immune activation in vivo, emphasizing its role and impact as an independent therapeutic platform. The treatment protocol was depicted in **Figure** **7**A. The tumor growth curves for both groups of mice during the entire treatment period were shown in Figure 7B,C, with the average tumor growth curve presented in Figure 7D. The results indicated that mMPMs significantly inhibited tumor growth compared to the control group. The statistical analysis of tumor weights further confirmed the anti‐tumor effects of mMPMs (Figure 7E). Additionally, the body weight of the mice increased during the treatment period and no obvious organ damage was observed in histological sections at the end of treatment, indicating good in vivo biocompatibility of mMPMs (Figure 7F; Figure S23, Supporting Information). Hematoxylin and eosin (H&E) staining results of the tumor tissues showed that owing to their intrinsic immunoregulatory effects, mMPMs effectively inhibited tumor growth compared to the control group, as shown by less tumor cell nuclear infiltration and smaller tumor size (Figure 7G). Furthermore, green fluorescence was detected in terminal deoxynucleotidyl transferase‐mediated deoxyuridine triphosphate nick end labeling (TUNEL) staining, suggesting that mMPMs could trigger apoptosis in tumors, with ≈20% of cells showing TUNEL positivity (Figure 7H; Figure S24, Supporting Information). These results demonstrated the capability of mMPMs to effectively suppress tumor growth. Subsequently, immune analysis of the tumor tissues after the treatment was conducted to reveal the potential ability of mMPMs in activating immune systems. The polarization of macrophages in tumors was evaluated by flow cytometry. The gating strategies of fluorescence‐activated cell sorting were depicted in Figure S25 (Supporting Information). We found that the proportion of M1 macrophage in the tumors of mMPMs‐treated mice increased significantly, showing more than a three‐fold increase compared to the control group. In contrast, there was no significant difference in the proportion of M2 macrophages between the two groups (Figure 7I,J). This suggested that mMPMs had the potential to induce the polarization of tumor‐associated macrophages from the M2 phenotype to the M1 phenotype. As mentioned previously in Figure 5E, we presented an enrichment analysis related to T cells, highlighting the potential regulatory effect of mMPMs on the tumor immune microenvironment, such as the activation of T cells. Therefore, we further analyzed the changes in T cell‐mediated adaptive anti‐tumor immunity. The gating strategies for the analysis of lymphocytes are shown in Figure 7L. First, we studied the proportion of helper T cells (CD4^+^) and cytotoxic T cells (CD8^+^). They were two important anti‐tumor T cell subtypes, with helper T cells aiding cytotoxic T cells in killing tumor cells. Consistent with proteomic analysis, CD4^+^ T cells in mMPMs group were significantly higher than in the control group, indicating that mMPMs could promote the infiltration of CD4^+^ T cells in tumors. A similar phenomenon was observed in CD8^+^ T cells, with a higher proportion in the mMPMs group (Figure 7K). Additionally, the results of immunofluorescence staining showed a significant increase in green (CD4^+^ T cells) and red (CD8^+^ T cells) fluorescence in the tumors of mMPMs‐treated mice compared to the control group (Figure 7M). This indicated that mMPMs can enhance both helper and cytotoxic T cell subtypes, ultimately achieving T cell‐mediated immune activation. Surprisingly, we observed extensive lung metastatic lesions in the control group from the lung tissue sections, but few in the mMPMs group (Figure 7N). The result indicated that mMPMs can inhibit the metastasis of tumors due to their significant anti‐tumor effects. In summary, these results demonstrated that mMPMs can regulate macrophage M1 polarization and activate T cells to boost tumor immunotherapy.

Overall, this study successfully constructed immunomodulatory metal‐phenolic microcapsules through the one‐step assembly of HADA and Fe^III^ onto the template of Man‐BSA MBs. The developed approach innovatively imparted immunoregulatory bioactivity to capsules by glycating the template of BSA MBs and subsequently incorporating the glycated template within the capsule structure. To begin, a 79‐glycation site‐rich Man‐BSA was prepared by restricting the Maillard reaction process to its early stages. Mass spectrometry analysis confirmed the presence of abundant lysine glycation sites on Man‐BSA, with a maximum percentage of glycation sites of 94.88%. These lysine glycation sites have been discovered to induce the production of inflammatory factors in macrophages. Subsequently, based on Man‐BSA MBs generated through sonication, the one‐step assembly of HADA and Fe^III^ onto their surfaces was achieved through hydrophobic and coordination interactions. Upon the spontaneous escape of air from the interior of the bubbles, Man‐BSA was retained within the capsules, resulting in the formation of numerous metal‐phenolic microcapsules with immunomodulatory capabilities. In vitro cell experiments and proteomic analysis collectively revealed that Man‐BSA enhanced the internalization of these capsules by macrophages, accompanied by a notable increase in macrophage proliferation. These events resulted in the release of ROS and pro‐inflammatory cytokines, thereby activating macrophages with M1 phenotype, as evidenced by significant expression of M1 markers at gene and protein levels. The underlying mechanisms of glycated capsules on macrophage polarization were investigated in depth. It was found that these capsules promoted phagocytosis of macrophages and then activated cellular ROS and pro‐inflammatory mediators accumulation through the NF‐κB pathway to modulate macrophage‐mediated immune responses. Furthermore, in vivo experiments demonstrated that mMPMs could mediate macrophage M1 polarization and T‐cell activation to improve the immunosuppressive microenvironment and suppress tumor growth.

## 3 Conclusion

In conclusion, this study developed mannose‐glycated metal‐phenolic microcapsules with inherent abilities to regulate macrophage M1 polarization. By precisely adjusting reaction parameters, we controlled early Maillard reactions between mannose and BSA, yielding Man‐BSA with minimal AGEs. Lysine glycation sites on Man‐BSA were abundant, and they have been reported to promote the production of inflammatory factors. Thus, immunomodulatory metal‐phenolic microcapsules were constructed through the assembly of dopamine‐modified HA and Fe^III^ on these Man‐BSA microbubbles, and then Man‐BSA was retained to impart immunomodulatory activity to the capsule. Upon interacting with macrophages, the capsules induced ROS production and pro‐inflammatory cytokine secretion, promoting the polarization of macrophages toward the M1 phenotype. The proteomic analysis combined with in vitro and in vivo experiments demonstrated that these capsules promoted macrophage M1 polarization, activated T cells, and reshaped the tumor microenvironment, showing satisfactory anti‐tumor effects. Notably, these novel capsules exhibited intrinsic immune‐modulating properties, obviating the need for additional immune agents. This unique feature allowed for saving the structural space of the capsules to accommodate more therapeutic drugs, making them a promising delivery platform for macrophage‐based immunotherapy.

## 4 Experimental Section

### Materials

The bovine serum albumin (BSA, V900933) and collagenase IV (C5138) were purchased from Sigma‐Aldrich Chemicals Reagent Co. Ltd., USA. Hyaluronic acid (HA, MW = 400 000, RH445435) was obtained from Rhawn Chemicals Reagent Co. Ltd., China. D‐(+)‐mannose (M103969), dopamine hydrochloride (D103111), N‐Hydroxysuccinimide (H109330), N‐(3‐Dimethylaminopropyl)‐N’‐ethylcarbodiimmide hydrochloride (E106172), iron (III) chloride hexahydrate (FeCl_3_·6H_2_O, F419646), NH_4_HCO_3_ (A431333), Dithiothreitol (DTT, D104859), Indole‐3‐acetic acid (IAA, I101073), FITC (F106837) and Tris(hydroxymethyl)aminomethane (T591027) were bought from Aladdin Biochemical Technology Co. Ltd., China. Urea (69 137 761), Tween 20 (30 189 328), NaCl (10 019 328), NaOH (10 019 718), HCl (10 011 018), and ethylenediaminetetraacetic acid (EDTA, 10 009 617) were obtained from Sinopharm Chemical Reagent Co. Ltd., China. Calcein‐AM/PI double staining kit (KGA9501), reactive oxygen species assay kit (KGA7308), and CCK‐8 kit (KGA9305) were all obtained from KeyGEN Biotechnology Co. Ltd., China. Triton X‐100 (P0096), Actin‐Tracker Red (C2203S), DAPI (C1002), Hoechst 33 342 (C1022), total RNA extraction reagent (R0018S), QuickBlock™ Blocking Buffer (P0260), CD4 mouse monoclonal antibody (AG1393), CD8 alpha rabbit monoclonal antibody (AG1414) and Penicillin‐Streptomycin‐Gentamicin solution (C0224) were bought from Beyotime Biotechnology Co. Ltd., China. Goat anti‐rabbit IgG (SA00013‐2), phosphor‐ERK (28733‐1‐AP), and phosphor‐JNK (80024‐1‐RR) antibodies were purchased from Proteintech Group, Inc., USA. DNase I (1121MG010) was bought from Biofroxx, Germany. Hyaluronidase (H792266) was from Macklin Biotech Co. Ltd., China, and blood lysis buffer (555 899) was obtained from Becton, Dickinson and Company (BD). FC Block (130 092 575) was from Miltenyi Biotec, Germany. Antibodies for flow cytometry were purchased from Thermo, BD Biosciences or BioLegend, and the detailed information was described in corresponding locations. Dulbecco's modified Eagle's medium (DMEM), RPMI‐1640 medium, fetal calf serum (FBS, BC‐SE‐FBS07), 4% paraformaldehyde, and bicinchoninic acid (BCA) protein assay kit were obtained from BioChannel Biological Technology Co. Ltd., China. Milli‐Q water was used in the experiments.

### Preparation of Man‐BSA Solution

Man‐BSA solution was prepared by wet‐state heating using a modified procedure described in a previous study.^[^ ^47^ ^]^ Specifically, BSA solution (100 mg mL^−1^) was mixed with an equal concentration of mannose and then heated in a 65 °C water bath for 20 h. To investigate the influence of mannose concentration, temperature, and time of the Maillard reaction on the glycation degree and structure of BSA, BSA solutions (100 mg mL^−1^) with 25, 50, 100, or 200 mg mL^−1^ mannose were heated in a 65 or 70 C water bath for 5, 7, 10, 14, 20 or 24 h. The degree of glycation in the Man‐BSA solutions was initially assessed by measuring the peak absorbance at 420 nm and 294 nm using a UV‐vis spectrophotometer (Evolution 300PC, Thermo).

### Microbubble Preparation

Briefly, BSA or Man‐BSA solution with concentrations of 100 mg mL^−1^ was preheated in a thermostat water bath at 75 °C for 5 min. Subsequently, a 20KHz ultrasonic probe (Nanjing ATPIO Instruments Manufacture Co., Ltd) was positioned at the gas‐liquid interface of a 1 mL protein solution. The solution was sonicated at an 80% amplitude power setting (105 W) for ≈2 min, with an ultrasonic time of 3 s and an intermittent time of 3 s. Following preparation, the MB dispersions were centrifuged at 300 g for 10 min. The upper layer, predominantly containing MBs, was retrieved, mixed with 1 mL of Milli‐Q water, and then subjected to centrifugation for 25 min at 300 g. The top layer was again collected for the following microcapsule preparation. These prepared MBs were then analyzed using a UV‐vis spectrophotometer, particle size and zeta potential analyzer, fluorescence spectrometer, FTIR spectroscopy, and mass spectrometer (MS). The detailed methods of BSA MBs and Man‐BSA MBs samples used for MS/MS analysis have been described in previous studies.^[^ ^47^ ^]^

### Synthesis of HADA

As previously described, dopamine was conjugated to the carboxylic acids of HA using the carbodiimide coupling reaction to synthesize HADA.^[^ ^48^ ^]^ Typically, 0.5 g HA was dissolved in Milli‐Q water (20 mg mL^−1^), and the pH value was adjusted to 5.5 using the HCl solution (1.0 M). Next, EDC (9.56 mg/mL) and NHS (5.72 mg mL^−1^) were slowly added to the resulting HA solution and stirred at room temperature for 15 min. Subsequently, dopamine hydrochloride (11.32 mg mL^−1^) was introduced into the solution. The mixture was agitated at ambient temperature in an argon environment for 24 h. After the reaction, the solution was transferred to a dialysis bag (14kDa, regenerated cellulose membrane, Biosharp) and dialyzed against ≈5 L of aqueous HCl solution (pH 5.0) for 3 days followed by dialysis against Milli‐Q water for 12 h. The final product underwent lyophilization and was kept at −20 °C until further use. The effectiveness of catechol modification was evaluated by analyzing the absorption peak of the HADA solution at 280 nm using a UV‐vis spectrophotometer. To quantify the catechol groups on HA, a standard curve was generated by measuring the absorbance at 280 nm of dopamine solutions at various concentrations. In addition, ^1^H NMR spectroscopy was performed in D_2_O at 25 °C using an AVANCE III HD 600MHz spectrometer (Bruker) to confirm the conjugation of catechol groups to HA. The extent of dopamine substitution in HA was determined by evaluating the integral area ratio between the relevant peaks originating from H atoms in the aromatic rings of dopamine and those in the methylene group of HADA.

### HADA‐Fe^III^ Coating on BSA or Man‐BSA Microbubble

Aliquots (50 µL) of HADA (17.7 mg mL^−1^) and FeCl_3_·6H_2_O (5.7 mg mL^−1^) solutions were added to the BSA or Man‐BSA MBs dispersions (390 µL) to yield the following final concentrations (BSA: 10.5 mg/mL, HADA: 1.77 mg mL^−1^, FeCl_3_·6H_2_O: 0.57 mg/mL in 0.5 mL of MilliQ water). The pH of the resulting MBs suspension was raised by adding 10 µL of Tris(hydroxymethyl)aminomethane solution (200 mg mL^−1^). The suspension was vigorously mixed using a vortex mixer for 60 s after each addition of HADA, FeCl_3_·6H_2_O, and Tris solution. The mixture solutions were washed with water three times to remove excess HADA, FeCl_3_ and MBs. The washing steps involved centrifuging the mixed solutions at the relative centrifugal force of 2000 g for 10 min and then removing the supernatant and top layer of MBs. The capsule templated by BSA MBs was named MPMs, while those templated by Man‐BSA MBs were named mMPMs. The concentration of the resulting microcapsule suspension was determined using the BCA method. Specifically, the calibration curve of BSA was established following the manufacturer's instructions. Then 200 µL of BCA working solution was added to 20 µL of the capsule solution. The mixture was incubated at 37 °C for 30 min, and the absorbance of the solutions was measured at 562 nm. Additionally, the number of microcapsules in the suspension was determined using a counting chamber (hemocytometer). For fluorescent labeling of microcapsules, BSA or Man‐BSA was labeled with FITC using a standard protocol.^[^ ^49^ ^]^ Then FITC‐BSA or FITC‐Man‐BSA (100 mg/mL) was used for microcapsule fabrication as described above while keeping the other variables constant. Confocal laser scanning microscopy was used to observe the capsules.

### Characterization

(I) Bright‐field optical microscopy: Capsule samples were gathered post‐preparation and visualized using a bright‐field optical microscope (MF52, Guangzhou Mingmei Optoelectronics Technology Co., Ltd). (II) SEM: Dried capsules were employed for SEM examination (Zeiss 11 003 457, Ultra Plus, Germany), energy dispersive X‐ray spectroscopy (EDS) profiles, and element mapping. (III) AFM: Standard scans were performed in intermittent contact mode with silicon cantilevers utilizing a BRUKER Dimension Icon. A drop of the capsule solution was dropped onto a clean silicon wafer, and the sample was prepared after natural drying. The thickness and roughness of microcapsules were analyzed using Nanoscope Analysis software. (IV) TEM: TEM (Thermoscientific) measurements were operated at 200 kV to obtain TEM and HAADF images and EDX mapping data. (V) CLSM: CLSM images of capsules were acquired with a Leica TCS SP8 laser scanning microscope. (VI) UV‐vis spectrophotometer: UV‐vis absorption measurements were carried out on the UV‐vis absorption spectrophotometer. (VII) Particle size and zeta potential analyzer: Zeta‐potential and size distribution measurements of BSA MBs, Man‐BSA MBs and capsules were carried out in the water by using a Zetasizer Nano ZSE (Malvern). (VIII) Fluorescence spectrometer: The fluorescence spectrometer (Fluoromax‐4) equipped with a quartz cell of 1.0 cm pathlength was used for fluorescence measurement of BSA MBs and Man‐BSA MBs. The excitation wavelength was set at 285 nm, with emission spectra being captured within the range of 300 to 500 nm at room temperature (21 °C). The fluorescence of the water was subtracted to correct for background fluorescence. (IX) FTIR spectroscopy: BSA MBs, Man‐BSA MBs and the corresponding capsules (i.e., MPMs and mMPMs) were analyzed using the FTIR spectrometer (Nicolet). To analyze the secondary structure, we used the second‐derivative method to enhance the resolution of the amide I band, employing Origin 2024 software (OriginLab, Northampton, MA, USA). Subsequently, the Gaussian function was applied to fit and assess the positions of the distinct peaks within the curve spanning 1700‐1600 cm^−1^. (X) MALDI‐TOF mass spectrometer: Molecular weight analysis of BSA MBs and Man‐BSA MBs was conducted using a MALDI‐TOF mass spectrometer (Bruker, Ultraflextreme, USA) operating in positive ion mode. IR, which represented the mean quantity of mannose molecules linked to each protein molecule, was employed to evaluate the extent of protein glycation.^[^ ^50^ ^]^ Through the comparison of MW alteration observed between native BSA and glycated BSA, the IR of mannose to BSA could be deduced as follows:

(1) $$\mathrm{IR}=\left(MW_{\mathrm{Man}-\mathrm{BSA}\;\mathrm{MBs}}-MW_{\mathrm{BSA}\;\mathrm{MBs}}\right)/162$$

The MW of mannose attached to BSA was denoted as 162, MW_Man‐BSA MBs_ represented the molecular weight of glycated BSA MBs, while MW_BSA MBs_ corresponded to the molecular weight of non‐glycated BSA MBs. (XI) Ultra‐high resolution biological mass spectrometer: The hydrolyzed samples of BSA MBs and Man‐BSA MBs were analyzed using a Thermo Fisher Orbitrap Eclipse Mass Spectrometer (Thermo Scientific, Waltham, MA, USA) for peptide mapping in positive mode. Peptides' MS and MS/MS spectra were acquired through full scan mode, with a scan range of 350 to 1800 m/z and a survey scan resolution of 70 000. The initial RAW file was processed using Xcalibur. For protein glycation site identification, Proteome Discoverer 2.4 was utilized. Based on the mass shift, the amino acid residues of the BSA molecule can undergo mono‐glycation or dual‐glycation by mannose, enabling the identification of potential mannose glycation sites. In theory, when a peptide is glycated with a single mannose molecule and loses a water molecule, the m/z values for peaks with charges of 1, 2, 3, 4, or 5 will display a mass increment of 163.0606 Da, resulting in m/z changes of 163.0606, 81.5303, 54.3535, 40.7652, and 32.6121 Da, respectively. In the case of dual‐glycated peptides, the mass shift will be 326.1212 Da. The search parameters included the following: enzyme = Trypsin, fixed modification = carbamidomethyl (C), variable modification = oxidation (M), N‐linked glycation (R, K, H, Q, and N), Double‐mannose (K, Q, H, N, and R), and Advanced glycation end products (N, K, Q, and W). To evaluate the extent of glycation for each peptide, the percent glycation was calculated using the following formula:^[^ ^50^, ^51^ ^]^

(2) $$\begin{array}{cc} & \mathrm{Glycation}\left(\%\right)\\ & =\mathrm{The}\;\mathrm{intensities}\;\mathrm{of}\;\mathrm{modified}\;\mathrm{peptide}\;\mathrm{in}\;\mathrm{the}\;\mathrm{Man}-\mathrm{BSA}\;\mathrm{MBs}\;\mathrm{sample}/\\ & \;\mathrm{The}\;\mathrm{intensities}\;\mathrm{of}\;\mathrm{the}\;\mathrm{corresponding}\;\mathrm{peptide}\;\mathrm{in}\;\mathrm{the}\;\mathrm{BSA}\;\mathrm{MBs}\;\mathrm{sample} \end{array}$$

In addition, frequency logos were obtained using WebLogo (https://Weblogo.berkeley.edu). Surface diagram and surface electrostatic potential mapping of BSA [PDB ID: 4F5S] was performed using the APBS plugin for PyMOL. The distribution of electric potential across a protein surface was determined utilizing the Poisson‐Boltzmann equation.^[^ ^51^ ^]^

### Disassembly Experiments

The disassembly and stability experiments were carried out in solutions at different pH values (HCl: pH 5; PBS: pH 7; NaOH: pH 12). The different pH solutions containing the same number of microcapsules were incubated in an incubator shaker at 37 °C. After 2 days of incubation, the remaining microcapsules were counted by cytometer. To investigate the dominant interaction in mMPMs, capsule suspensions (1.0 × 10^7^capsule mL^−1^) in 100 mm urea, 100 mm Tween 20, 100 mm NaCl, or 100 mm EDTA solution were incubated in an incubator shaker at 37 °C for the desired time. The number of remaining microcapsules was counted by cytometer.

### Cell Culture

RAW 264.7,4T1, B16, and L929 cells were from Shanghai National Cell Bank and cultured in a medium supplemented with 10% FBS, 100 U penicillin mL^−1^, and 100 µg streptomycin mL^−1^ at 37 °C with 5% CO_2_ in a humidified environment.

To prepare CM derived from macrophages, the supernatants of the culture medium were collected after treatment with control (DMEM), MPMs, and mMPMs. These collected supernatants were subjected to centrifugation at 2000 rpm for 30 min to remove residual cells and substances. Subsequently, the solution was filtered using 0.22 µm filters (Millipore, USA) and stored at ‐20 °C until further use. To restore the serum depleted during macrophage culture, a 5% FBS ratio was introduced to the CM.

To obtain BMDMs, femurs and tibias were harvested from 8‐week‐old C57BL/6 mice. Muscular tissue was carefully removed using gauze, and the bones were subsequently immersed in a sterile dish containing 75% ethanol for 3 min for surface sterilization. The bones were then washed with PBS to remove residual alcohol. Using surgical scissors, both ends of the bones were cut off, and the bone marrow was flushed out with PBS using a 10 mL sterile syringe. The resulting cell suspension underwent red blood cell lysis and was centrifuged at 300 g for 5 min. The cell pellet was resuspended in a culture medium composed of RPMI‐1640 supplemented with 20% L929 conditioned supernatant, 1% Penicillin‐Streptomycin‐Gentamicin solution, and 10% FBS. At day 0, cells were seeded into 12‐well plates at a density of 5 × 10^5^ cells mL^−1^ in a total volume of 2 mL per well, gently mixed, and incubated at 37 °C in a humidified atmosphere with 5% CO_2_. At day 3, half of the culture supernatant was collected, and the remaining cells were pelleted by centrifugation. The cells were resuspended in 1 mL of fresh culture medium containing 20% L929 conditioned supernatant and returned to their original wells. At day 5, the cells were ready for use or could be cultured further. At this point, the cells were collected and analyzed by flow cytometry for confirmation of macrophage differentiation.

### Cell Viability

RAW 264.7 cells were cultured in a 96‐well plate with a density of 1 × 10^4^ cells per well. Subsequently, they were exposed to varying concentrations of BSA MBs, Man‐BSA MBs, MPMs, and mMPMs in a total volume of 100 µL for 24–72 h at 37 °C. 4T1 or B16 cells were cultured in macrophage‐derived CMs (Control CM, MPMs CM, and mMPMs CM) with a total volume of 100 µL for 24 to 72 h at 37 °C. Following the incubation period, the cell culture media were substituted with fresh DMEM or RPMI‐1640 medium (100 µL) supplemented with CCK‐8. Following an additional 2‐hour incubation, the absorbance of the sample at a wavelength of 450 nm was assessed with an Infinite M200 microplate reader (Detie, China). The relative cell proliferation level was calculated.

### Live/Dead Staining

RAW 264.7, 4T1 or B16 cells (4×10^3^ per well) were separately seeded into the 96‐well plates and cultured with corresponding materials or macrophage‐derived CMs in a total volume of 100 µL for desired times. Afterward, 20 µL of the working solution from the Calcein‐AM/PI Double Staining Kit was added to each well, followed by a 15‐minute incubation period at 37 °C. Subsequently, live and dead cells within the 96‐well plate were visualized and captured using a fluorescence microscope. The percentage of dead cells was determined from live/dead staining images by quantifying the number of live and dead cells using ImageJ software. The results were expressed as mean ± standard deviation (*n* = 5 independent EUs).

### Cell Internalization

RAW 264.7, 4T1, L929 cells or BMDMs were separately seeded in confocal dishes at a density of 5000 cells per well and incubated at 37 °C overnight. FITC‐labeled capsules were introduced to each well at a ratio of 100 capsules to 1 cell. At specified time intervals, the cells were rinsed thrice with PBS to remove unbound capsules, then fixed using 4% paraformaldehyde at room temperature for 15 min. Cells were permeabilized with 0.1% Triton X‐100 for 5 min before being treated with Actin‐Tracker Red for one hour at room temperature to stain the actin cytoskeleton. Nuclear staining was performed using a DAPI solution for 5 min. Images of the stained samples were acquired using either a CLSM or a fluorescence microscope.

### ROS Detection

Cellular ROS production was assessed using DCFH‐DA. RAW 264.7 cells treated by various conditions (i.e., negative control, MPMs, and mMPMs) were co‐incubated with DCFH‐DA in fresh serum‐free medium at 37 °C for 20 min. Following that, the cells were treated with Hoechst staining solution according to the manufacturer's instructions. After thorough washing to remove excess probe and staining solution, the cells were observed using fluorescence microscopy. The intensity of fluorescence was determined using ImageJ software.

### Gene Expression Analysis Using qPCR

Gene expression of *CD206*, *CCR7*, *iNOS*, *TNF‐α*, *IL‐1β*, *IL‐10*, and *ARG‐1* was evaluated by qPCR. RAW 264.7 cells or BMDMs with a density of 2×10^5^ per mL were treated with the following: positive control (LPS: 300 ng mL^−1^), negative control (normal cell culture medium), MPMs (200 µg mL^−1^), and mMPMs (200 µg mL^−1^) for 48 h. Subsequently, procedures including total RNA extraction, reverse transcription (Toyobo, Japan), and qPCR analysis were carried out. The primer sequences, sourced from Genescript (Nanjing, China), were detailed in Table S4 (Supporting Information). The comparative C_T_ method (2^−∆∆C^_T_ method) was used to analyze qPCR data.

### Flow Cytometry Tests

RAW 264.7 cells (1×10^6^ per mL) were treated with FITC‐MPMs (200 µg mL^−1^), and FITC‐mMPMs (200 µg mL^−1^) for 2, 12, 24 h. Subsequently, the cells were collected, rinsed with a PBS solution, and prepared for flow cytometry examination using the BD LSRFortessa X‐20 system from Becton Dickinson and Company, USA. For macrophage polarization experiments, RAW 264.7 cells were pretreated to an M2 phenotype using IL‐4 (40 ng mL^−1^) and IL‐13 (20 ng mL^−1^) for 24 h. Subsequently, MPMs (200 µg mL^−1^), mMPMs (200 µg mL^−1^), and LPS (25 ng mL^−1^) were added and incubated with the above cells for 24 h. The untreated cells were set as negative control. RAW 264.7 cells, when induced by cell culture medium and LPS (300 ng mL^−1^), represented macrophages with M0 and M1 phenotypes, respectively. Cells were stained with Fixable viability dye EF506 (Thermo, 65‐0866‐14), anti‐mouse F4/80‐BV421 (Clone:BM8; 123 132; 1:200; BioLegend), anti‐mouse CD11b‐Super Bright 600 (Clone:M1/70; 63‐0112‐82; 1:200; Invitrogen), anti‐mouse CD206‐PE (Clone:C068C2; 141 706; 1:100; BioLegend), and anti‐mouse CD86‐FITC (Clone:GL1; 561 962; 1:200; BD Biosciences) antibodies. Flow cytometry analysis was performed on a full spectrum profiling flow cytometer (Cytek Biosciences).

### Western Blot Analysis

RAW 264.7 cells were exposed to MPMs or mMPMs for 48 h at a concentration of 200 µg mL^−1^, after which the cells were harvested for Western blot analysis. Western blot analyses of phospho‐ERK and phospho‐JNK were conducted according to standard protocols, with β‐actin serving as the loading control.

### Immunofluorescence Staining

In a 96‐well plate, RAW 264.7 cells or BMDMs were initially seeded at a density of 2000 cells per well and maintained in the cell culture medium. To detect M1 and M2 marker expression at the protein level, the cells were co‐cultured with MPMs (200 µg mL^−1^), mMPMs (200 µg mL^−1^), and LPS (25 ng mL^−1^) for 48 h, with the untreated cells serving as the negative control. Then, the cells were stained for iNOS (Affinity Bioscience, AF0199), ARG‐1 (proteintech, 66129‐1‐lg), and CD206 (proteintech, 18704‐1‐AP). For NF‐κB staining, following a 2‐h treatment with MPMs or mMPMs (200 µg mL^−1^), the cells were subjected to staining with an NF‐κB antibody (Cell Signaling, 8242). The staining procedure involved washing the cells with PBS, fixing them in 4% paraformaldehyde, permeabilizing with 0.1% Triton X‐100, and blocking with QuickBlock Blocking Buffer for 20 min. Subsequently, the samples were exposed to rabbit anti‐iNOS, mouse anti‐ARG‐1, rabbit anti‐CD206, or rabbit anti‐NF‐κB at 4 °C overnight. After PBS washing, the cells were treated with 488‐conjugated goat anti‐rabbit or anti‐mouse IgG for 1 h at room temperature, while DAPI was used for nuclear staining for 5 min. The samples underwent observation and imaging using the fluorescence microscope. The nuclear‐to‐cytoplasmic fluorescence ratio for NF‐κB and mean fluorescence intensity for iNOS, ARG‐1 and CD206 were quantified utilizing ImageJ software.

### ELISA

The levels of TNF‐α released from RAW 264.7 cells post‐treatment with MPMs, mMPMs, and LPS were analyzed by TNF‐α ELISA kits (Wuhan Huamei Biotechnology, China) following the manufacturer's instructions. The untreated cells served as the negative control.

### Proteomic Analysis

Following exposure to MPMs (200 µg mL^−1^) and mMPMs (200 µg mL^−1^) for 3 days, RAW 264.7 cells were gathered for proteomic investigation. The LFQ‐based proteomic analysis was employed to evaluate comprehensive protein expressions, utilizing established methodologies.^[^ ^52^ ^]^

### Animal Model

Female Balb/c mice aged 6–7 weeks were bought from Charles River Laboratory Animal Co., Ltd. (Zhengjiang, China) and maintained in specific pathogen‐free conditions with standard food and water. The animal experimental procedures were approved by the Animal Experimental Ethical Inspection Committee of Southeast University (approval no. 20 240 710 005). Tumor‐bearing mice were established by subcutaneously inoculating 100 µL of 2×10^7^ mL^−1^ 4T1 cells into the right flank of female mice. Upon reaching a tumor volume of ≈100 mm^3^, the mice were randomly divided into two groups: the mMPMs group and the control group (*n* = 5 biological replicates). Subsequently, the mice received intra‐tumoral injections of 100 µL of mMPMs or PBS every three days for a total of 3 doses, with a concentration of ≈15 mg kg^−1^ for the mMPMs injections. Tumor growth curves, tumor size, and mouse weight were recorded throughout the treatment period. Following 16 days of treatment, all mice were euthanized by inhalation of carbon dioxide (CO_2_) after induction of anesthesia with isoflurane, ensuring a humane and painless death. Tumor tissues were then harvested for weighing, after which they underwent histological analysis and flow cytometry.

### Flow Cytometry Analysis

Single‐cell suspensions from tumor tissues were prepared by digesting the samples in a culture medium that included 1 mg/mL collagenase IV, 0.2 mg mL^−1^ DNase I, and 0.2 mg mL^−1^ hyaluronidase at 37 °C for 1 to 1.5 h, using both mechanical and enzymatic methods. Following digestion, the single‐cell suspensions were harvested, centrifuged at 300 g for 5 min, and subjected to red blood cell lysis using a blood lysis buffer for 10 min at room temperature. After the lysis process, the cell suspensions were treated with FC Block on ice for 10 min. Subsequently, the single‐cell suspensions were stained with various antibodies, including Fixable viability dye EF506 (Thermo, 65‐0866‐14), CD45‐APC‐cy7 (Clone: 30‐F11; 557 659; 1:200; BD Biosciences), CD3e‐FITC (Clone: 145‐2C11; 553 061; 1:200; BioLegend), CD4‐BV786 (Clone: H129.19; 740 873; 1:200; BioLegend), CD8‐Percp‐cy5.5 (Clone: 53–6.7; 551 162; 1:100; BioLegend), CD11b‐Super Bright 600 (Clone: M1/70; 63‐0112‐82; 1:200; Invitrogen), F4/80‐BV421 (Clone: BM8; 123 132; 1:200; BioLegend), CD206‐PE (Clone: C068C2; 141 706; 1:100; BioLegend), CD86‐AF647 (Clone: GL‐1; 105 019; 1:200; BioLegend). Flow cytometry analysis was performed on a full spectrum profiling flow cytometer (Cytek Biosciences).

### Histological Staining

Post‐treatment, the BALB/c mice bearing 4T1 tumors were euthanized, and their tumors were harvested for histological staining in accordance with standard protocols, including the immunofluorescence analyses of CD4 and CD8, H&E, and TUNEL staining. Meanwhile, the heart, liver, spleen, lung, and kidney tissues from the mice were collected for H&E staining.

### Statistical Analysis

All results were expressed as mean ± standard deviation (SD). Statistical analyses were performed using GraphPad Prism 8 software. The normality of quantitative data was assessed using the Shapiro‐Wilk test and the homogeneity of variances was determined using the F‐test or Bartlett's test. For pairwise comparisons between two groups, the Student's *t*‐test (for normally distributed data) and Mann‐Whitney test (for non‐normally distributed data) were utilized. For comparisons among three groups, ANOVA (for normally distributed data) and Kruskal‐Wallis test (for non‐normally distributed data) were employed, as detailed in the figure legends. For experiments requiring statistical analysis, at least three separate experiments were conducted (*n* ≥ 3). ns (*p* > 0.05); *(*p* < 0.05); **(*p* < 0.01); ***(*p* < 0.001); ****(i < 0.0001).

## Conflict of Interest

The authors declare no conflict of interest.

## Supporting information



Supporting Information

## Data Availability

The data that support the findings of this study are available from the corresponding author upon reasonable request.
