# Supplementary material for: Mannose‐Glycated Metal‐Phenolic Microcapsules Orchestrate Phenotype Switch of Macrophages for Boosting Tumor Immunotherapy
Source: Adv Sci (Weinh). 2025 Feb 27;12(16):2415565. doi: 10.1002/advs.202415565 (PMC12021090; doi:10.1002/advs.202415565)
Supplement: Supplementary file 1 — Supporting Information [file ADVS-12-2415565-s001.docx]

Supporting Information

**Mannose-Glycated Metal-Phenolic Microcapsules Orchestrate Phenotype Switch of Macrophages for Boosting Tumor Immunotherapy**

*Xin Tan, Renwang Sheng, Weikun Li, Yinghua Tao, Zonghao Liu, Ning Yang, Syeda Safia Hashmi, Feiling Feng***, Fangzhou Liu***, Liqin Ge**

X. Tan, W. Li, Y. Tao, Z. Liu, N. Yang, S. Hashmi, L. Ge

State Key Laboratory of Digital Medical Engineering, School of Biological Science and Medical Engineering, Southeast University, Nanjing 210096, China

E-mail: [lqge@seu.edu.cn](mailto:lqge@seu.edu.cn)

L. Ge

Advanced Ocean Institute of Southeast University, Nantong 226000, China

F. Liu

Department of Head & Neck Surgery, Jiangsu Cancer Hospital & Jiangsu Institute of Cancer Research & The Affiliated Cancer Hospital of Nanjing Medical University, Nanjing 210029, China

E-mail: liufangzhou@njmu.edu.cn

R. Sheng

School of Medicine, Southeast University, Nanjing 210009, China

F. Feng

Department of Biliary Tract Surgery I, Shanghai Eastern Hepatobiliary Surgery Hospital, Navy Medical University, Shanghai 200438, China

E-mail: ffeiling@163.com


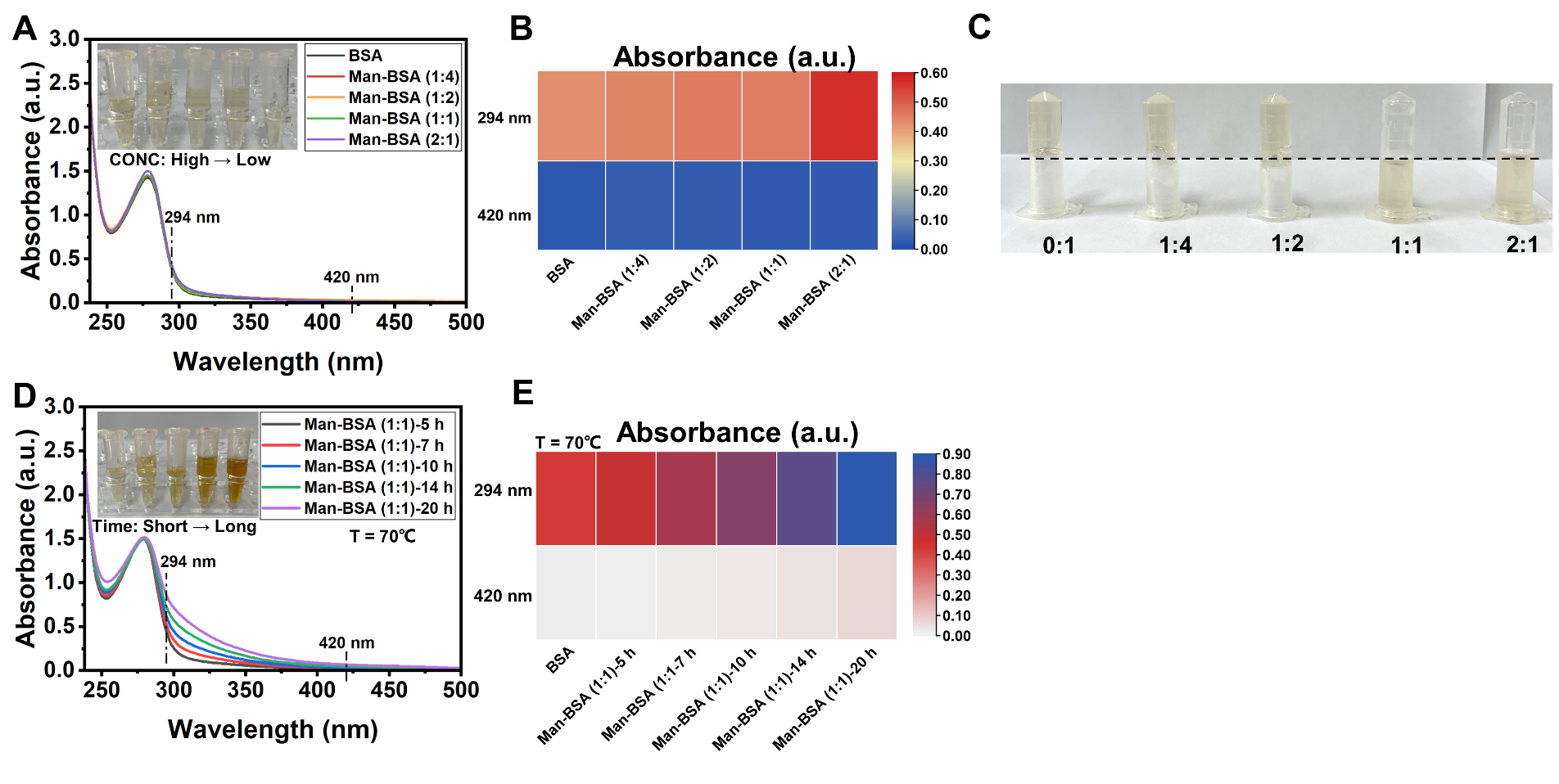


**Figure S1.** (A) UV−vis spectra of BSA and Man-BSA solutions with different mannose-to-BSA mass ratios, following heating at 70°C for 30 min. The inset shows the corresponding macrographs of these solutions. (B) Heatmap illustrating the absorbance values at 294 nm and 420 nm for BSA and Man-BSA solutions across different mannose-to-BSA mass ratios. (C) Macrophotographs of Man-BSA solutions prepared with different mannose-to-BSA mass ratios by heating at 70°C for 30 min. (D) UV−vis spectra of Man-BSA (1:1) obtained at various heating times at 70°C. The inset shows the corresponding macrographs. (E) Heatmap illustrating the absorbance values at 294 nm and 420 nm for BSA and Man-BSA (1:1) at different heating times at 70°C.


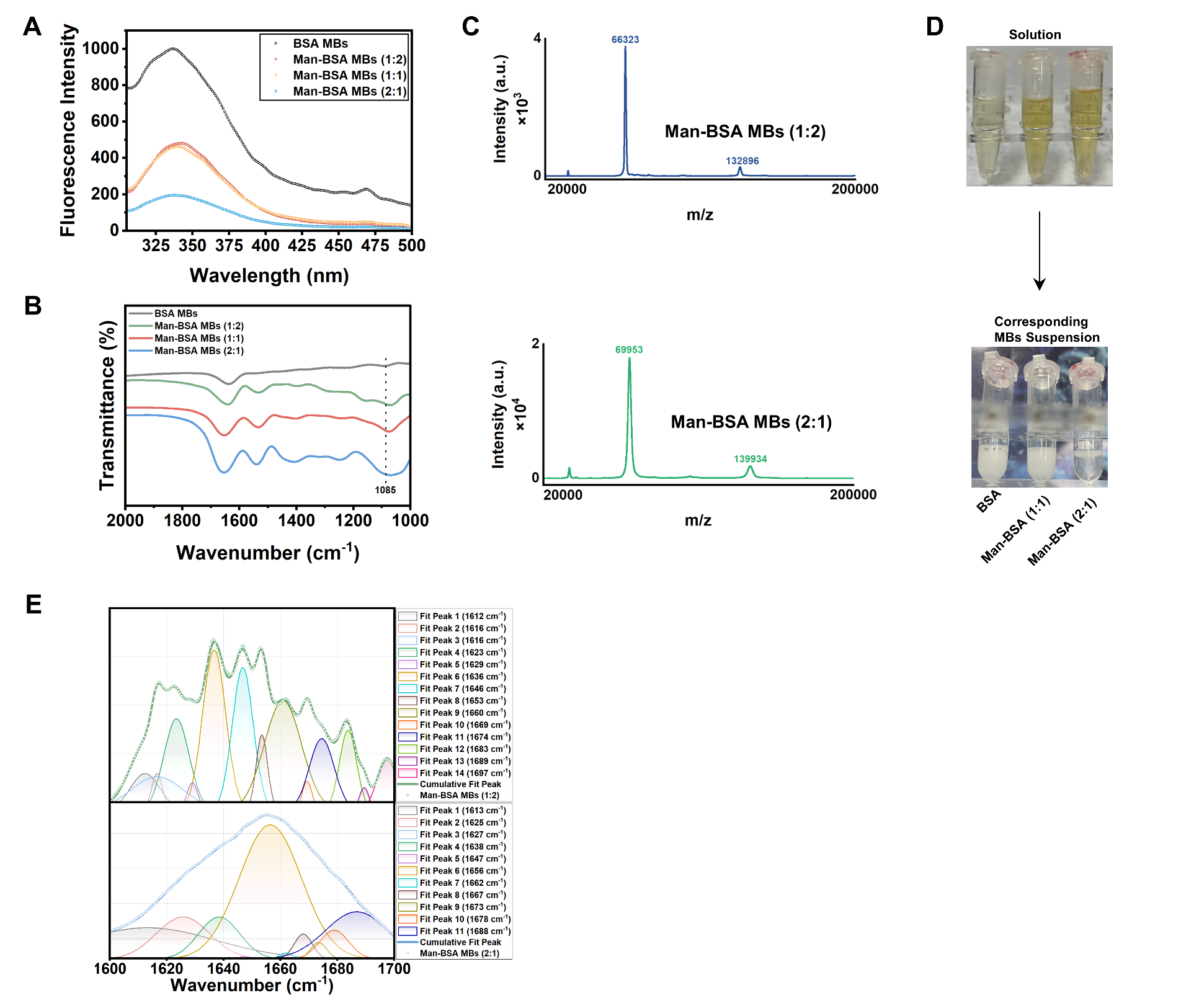


**Figure S2.** (A) Fluorescence spectra of BSA MBs and Man-BSA MBs with different mannose-to-BSA mass ratios. (B) The FTIR spectra of BSA MBs and Man-BSA MBs with different mannose-to-BSA mass ratios. (C) MALDI-TOF mass spectrometry analysis of BSA MBs (1:2 mannose-to-BSA ratio) and Man-BSA MBs (2:1 mannose-to-BSA ratio). (D) Macrophotographs of BSA, Man-BSA (1:1), and Man-BSA (2:1) solutions, along with their corresponding MBs suspensions. (E) Curve-fitting results of the FTIR spectra for BSA MBs (1:2) and Man-BSA MBs (2:1) in the region of the amide I band (1600-1700 cm⁻¹).


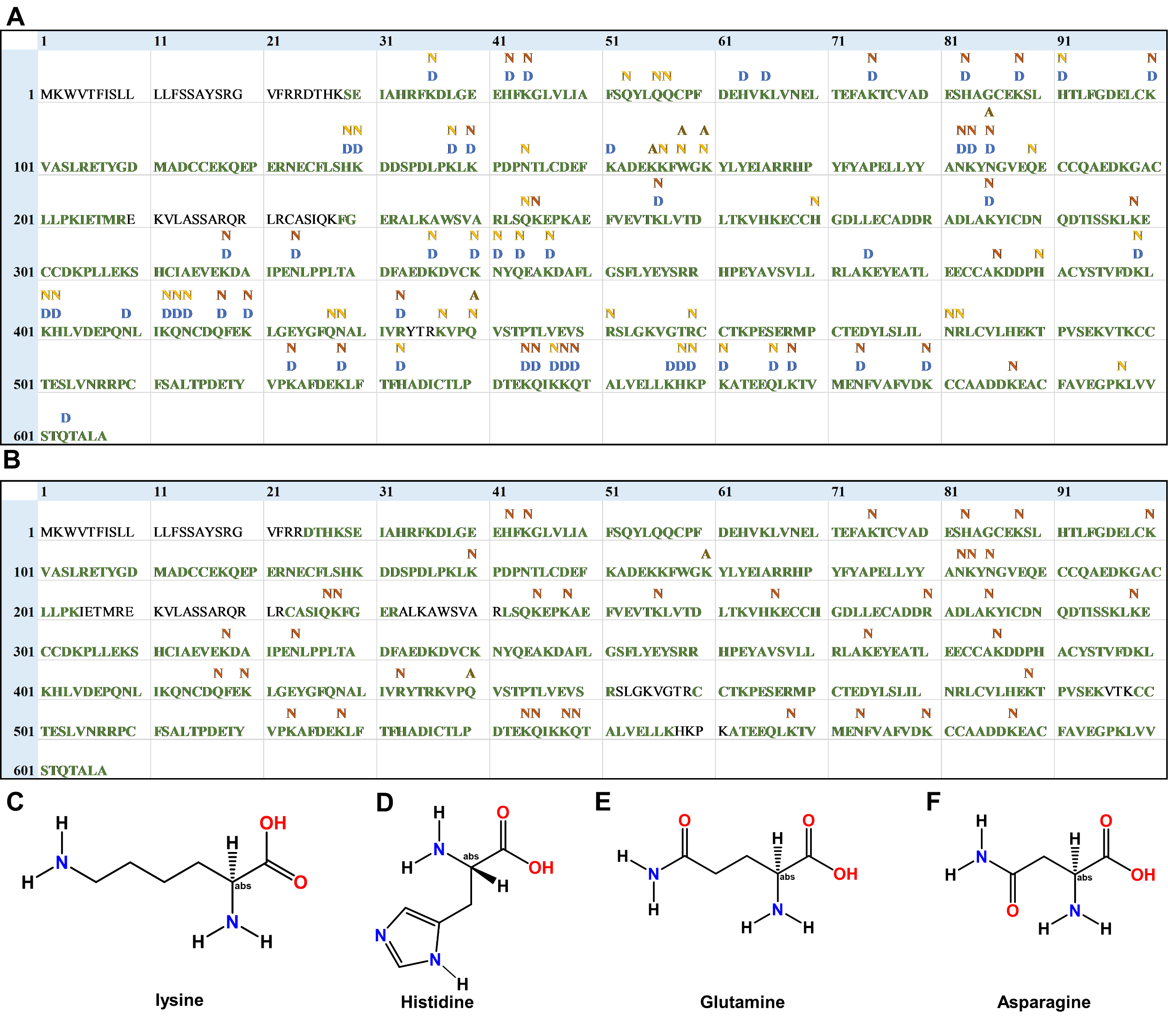


**Figure S3.** (A) All potential mono-glycation sites ("N"), dual-glycation sites ("D"), and AGEs sites ("A") in Man-BSA MBs. Yellow "N" indicates newly added mono-glycation sites, while orange "N" denotes originally existing glycation sites. (B) All potential mono-glycation and AGEs sites, of BSA MBs. (C-F) Chemical structure of lysine, histidine, glutamine, and asparagine.


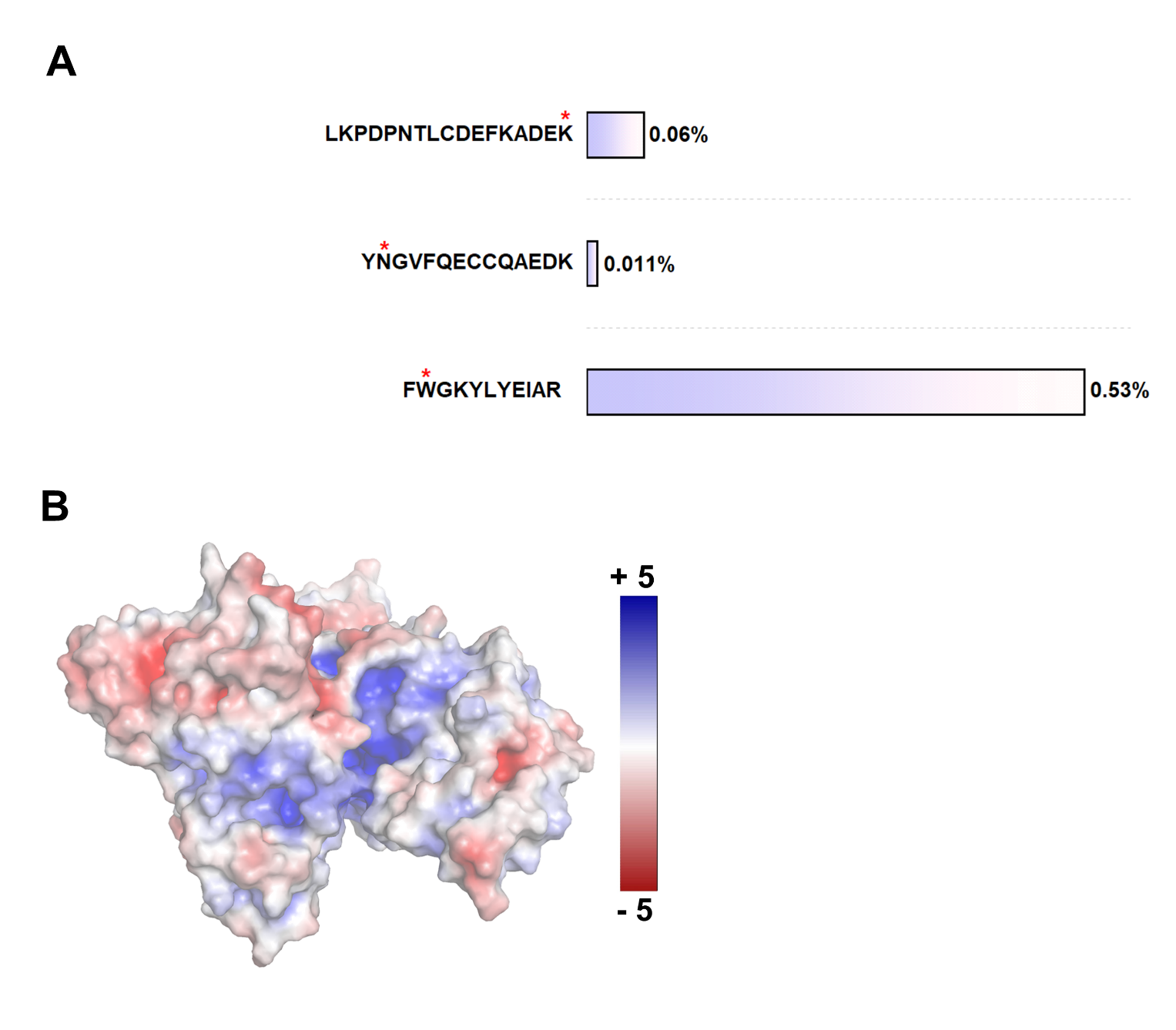


**Figure S4.** (A) The quantitative analysis of newly added AGEs sites on Man-BSA MBs. The glycation sites are marked by the symbol *. (B) The surface electrostatic potential mapping of BSA.


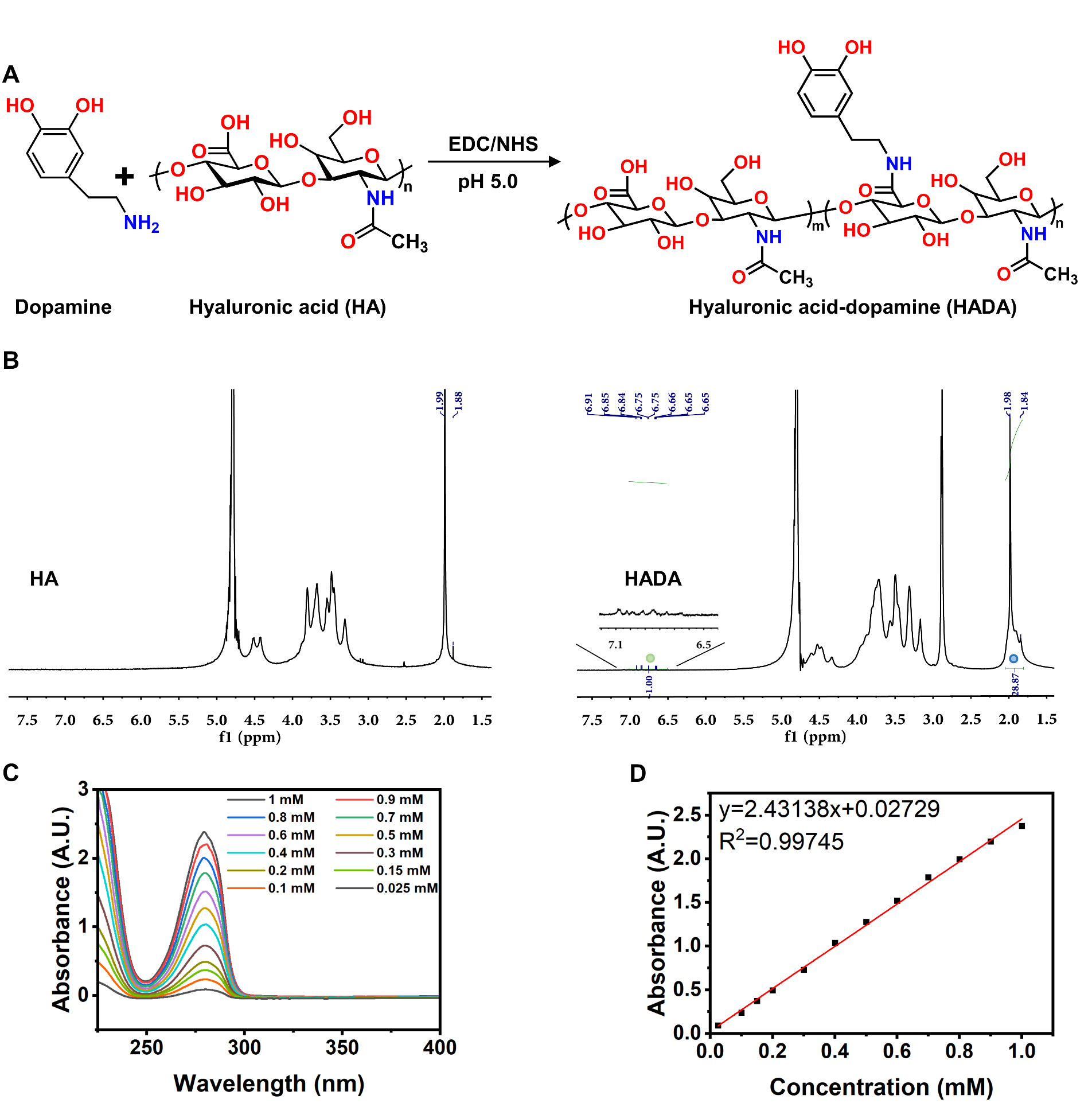


**Figure S5.** Characterization of the synthesized HADA. (A) The synthesis details of HADA. (B) ^1^H NMR spectrum of HA and the synthesized HADA. (C) UV-vis spectra of dopamine with different molar concentrations. (D) Standard absorption curve of dopamine in water by UV-vis spectrum.


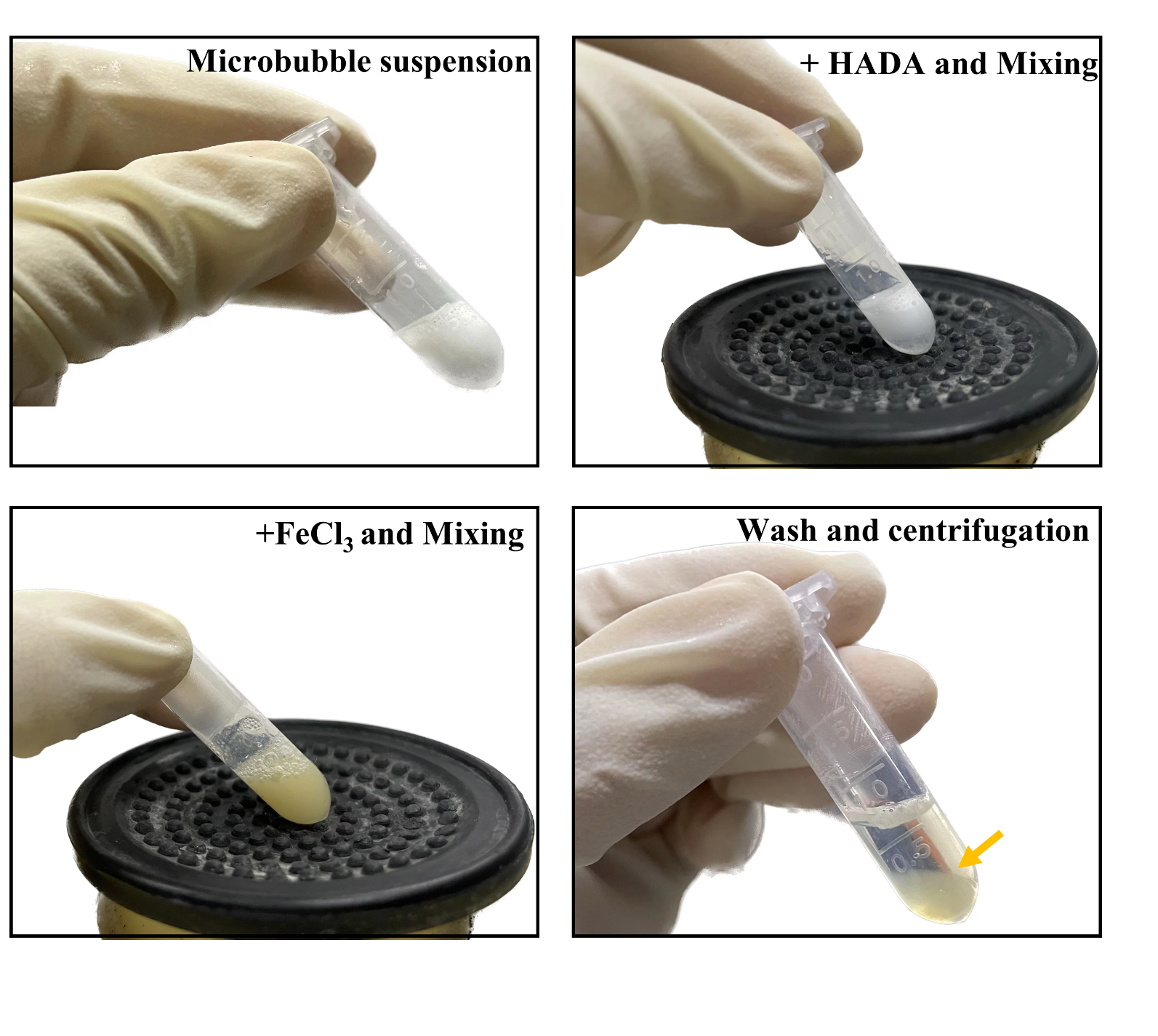


**Figure S6.** The preparation process of mMPMs templated by Man-BSA microbubbles.


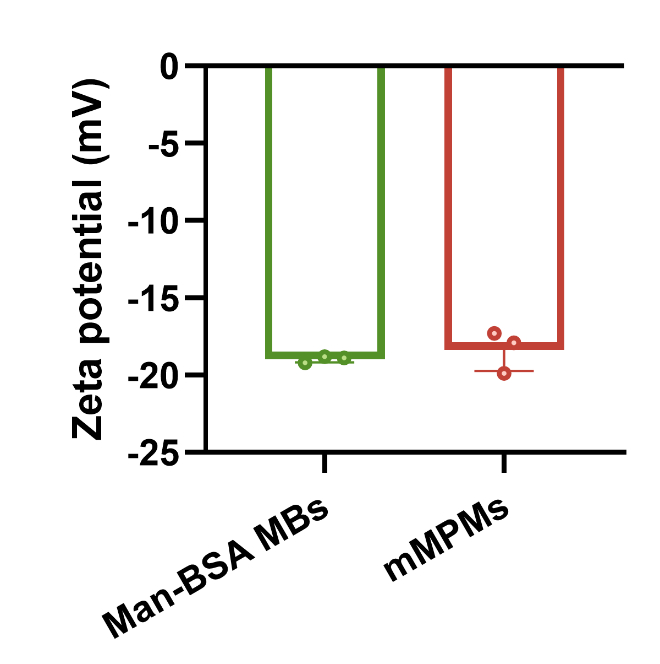


**Figure S7.** Zeta potential of Man-BSA MBs and mMPMs (n = 3 samples).


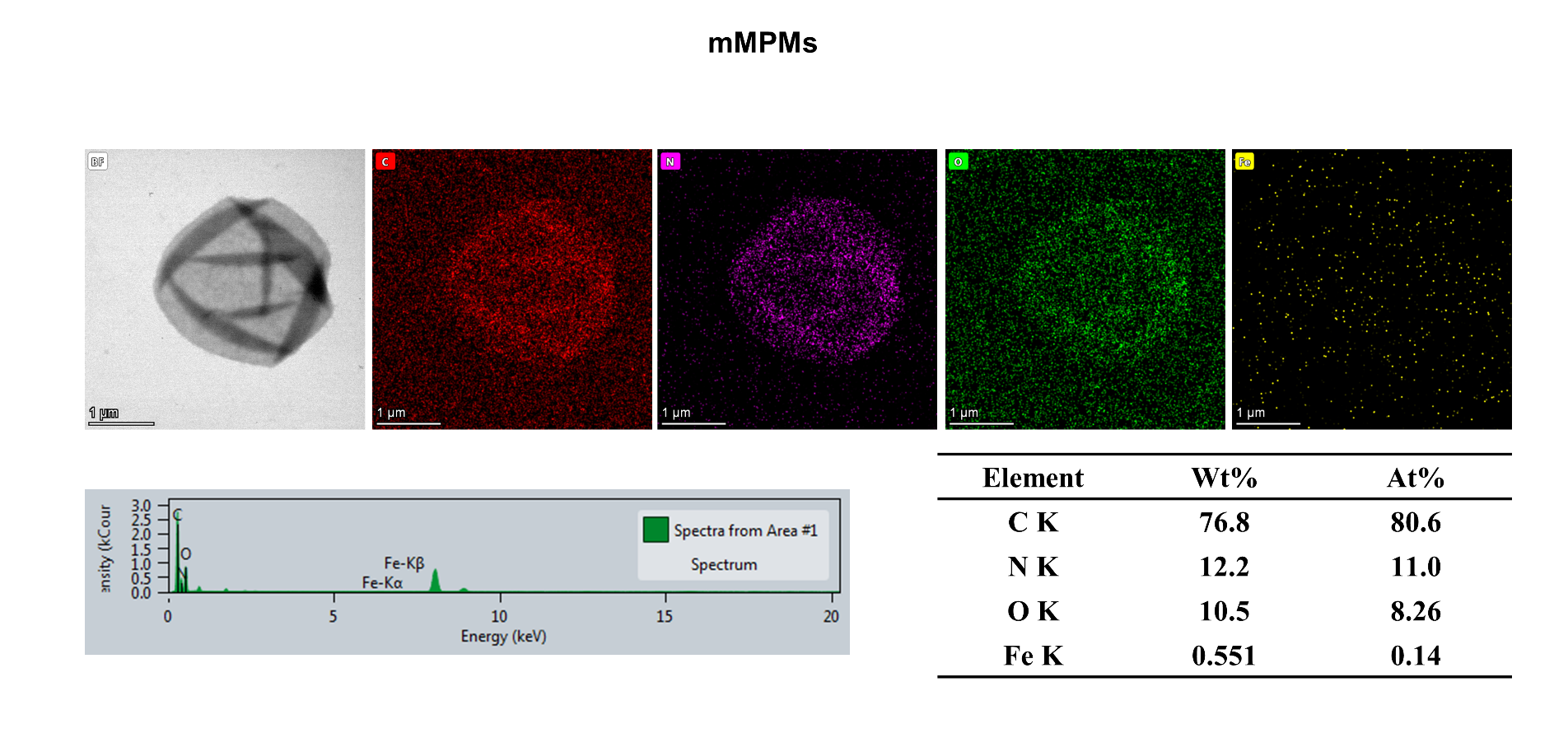


**Figure S8.** TEM, EDX elemental mapping and corresponding element analysis of mMPMs.


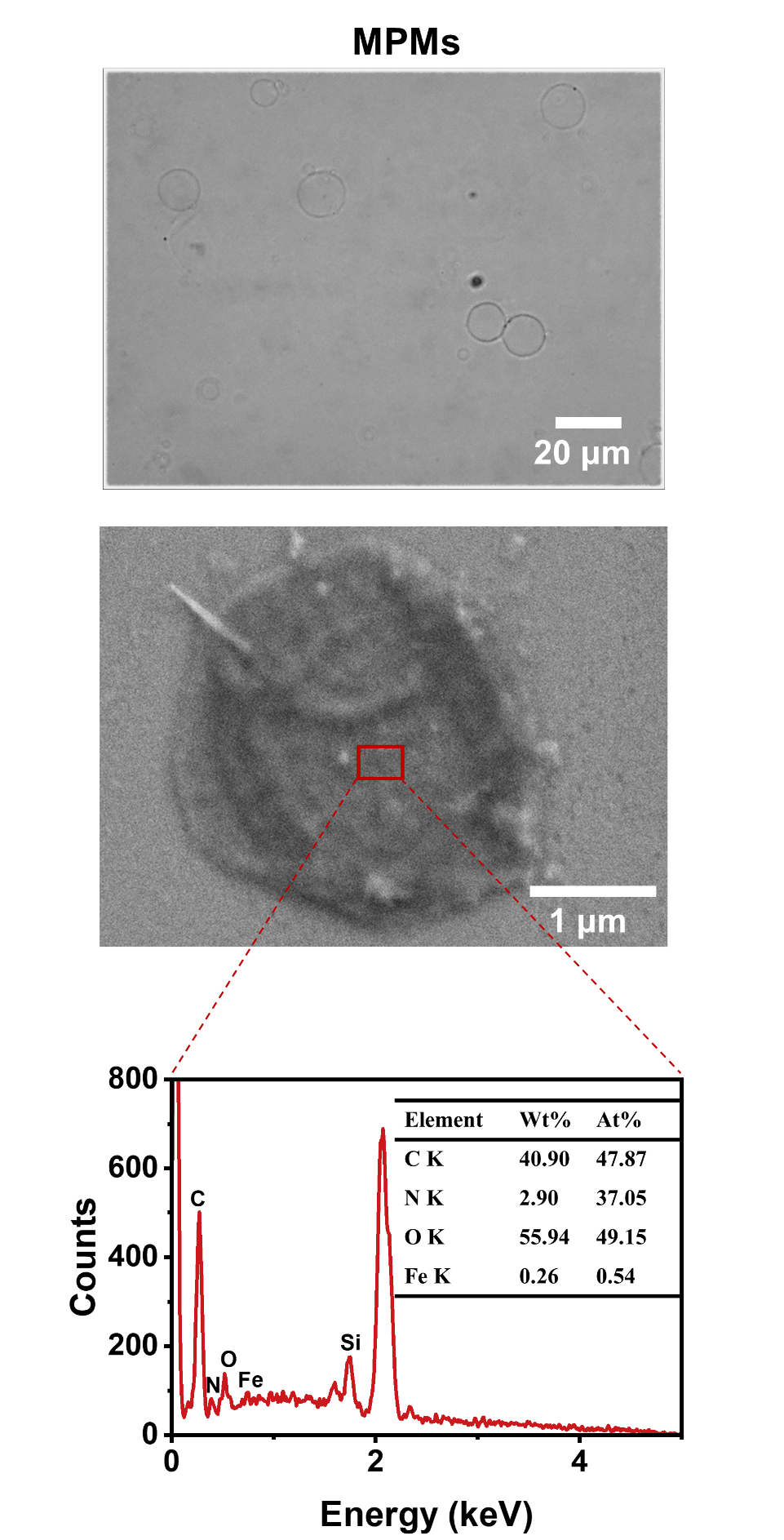


**Figure S9.** Optical microscopy image, TEM image and corresponding element analysis of MPMs.


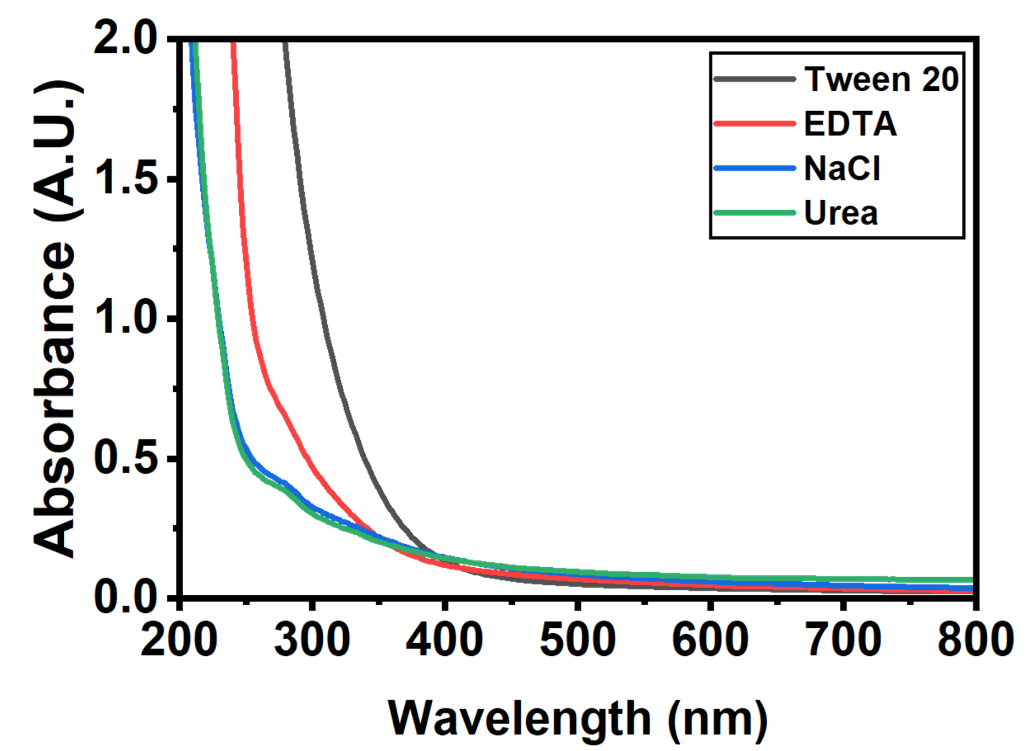


**Figure S10.** UV-vis spectra of mMPMs in the presence of 100 mM of urea, Tween 20, NaCl, or EDTA.


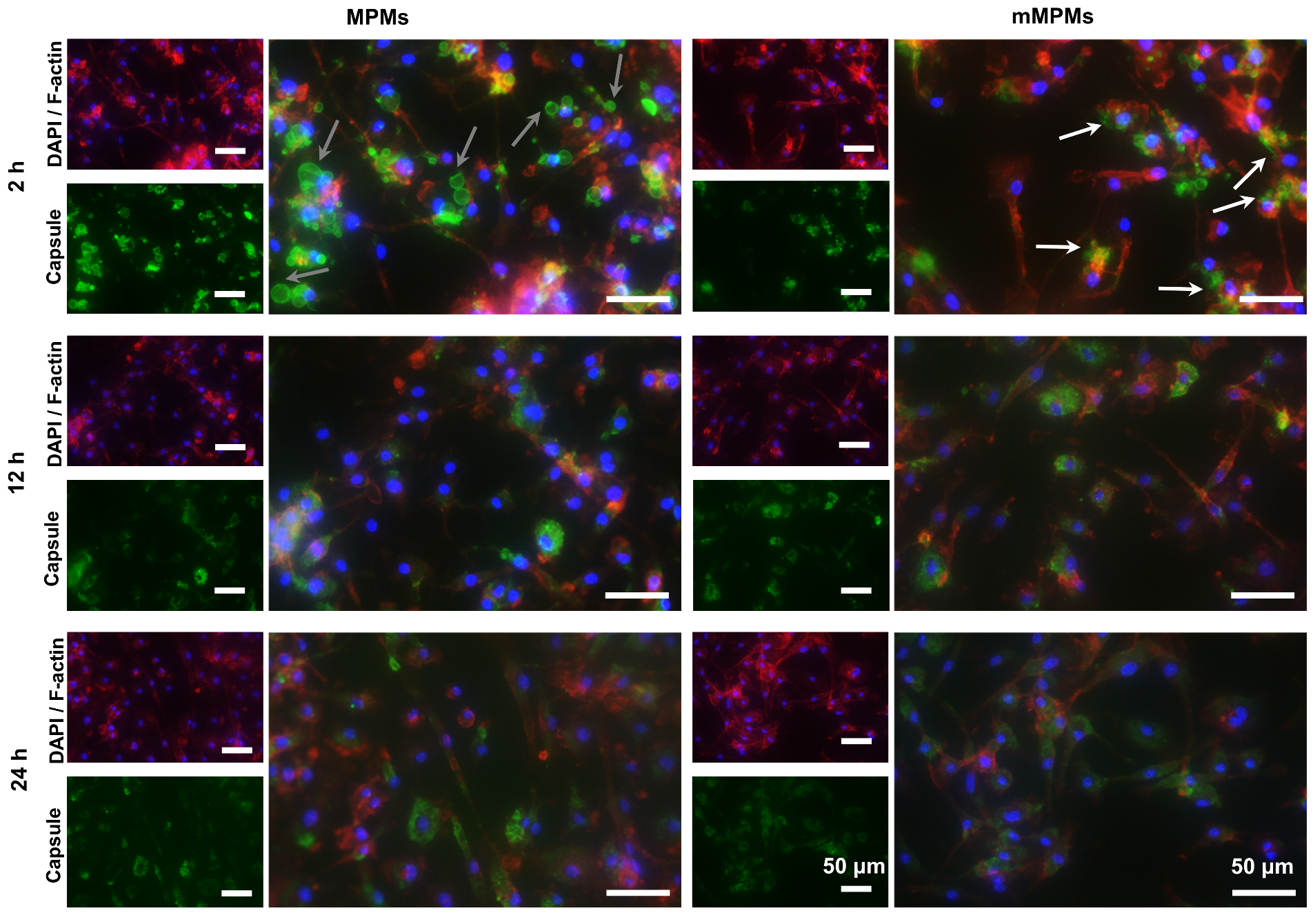


**Figure S11.** Fluorescence microscopy images of BMDMs following exposure to MPMs or mMPMs for 2, 12, and 24 h. The microcapsules are stained with FITC (green), while cell nuclei and cytoskeleton are visualized in blue (DAPI) and red (Actin-Tracker Red), respectively. White arrows indicate microcapsules that are interacting with or have been internalized by the cells, whereas gray arrows point to microcapsules remaining outside the cells.


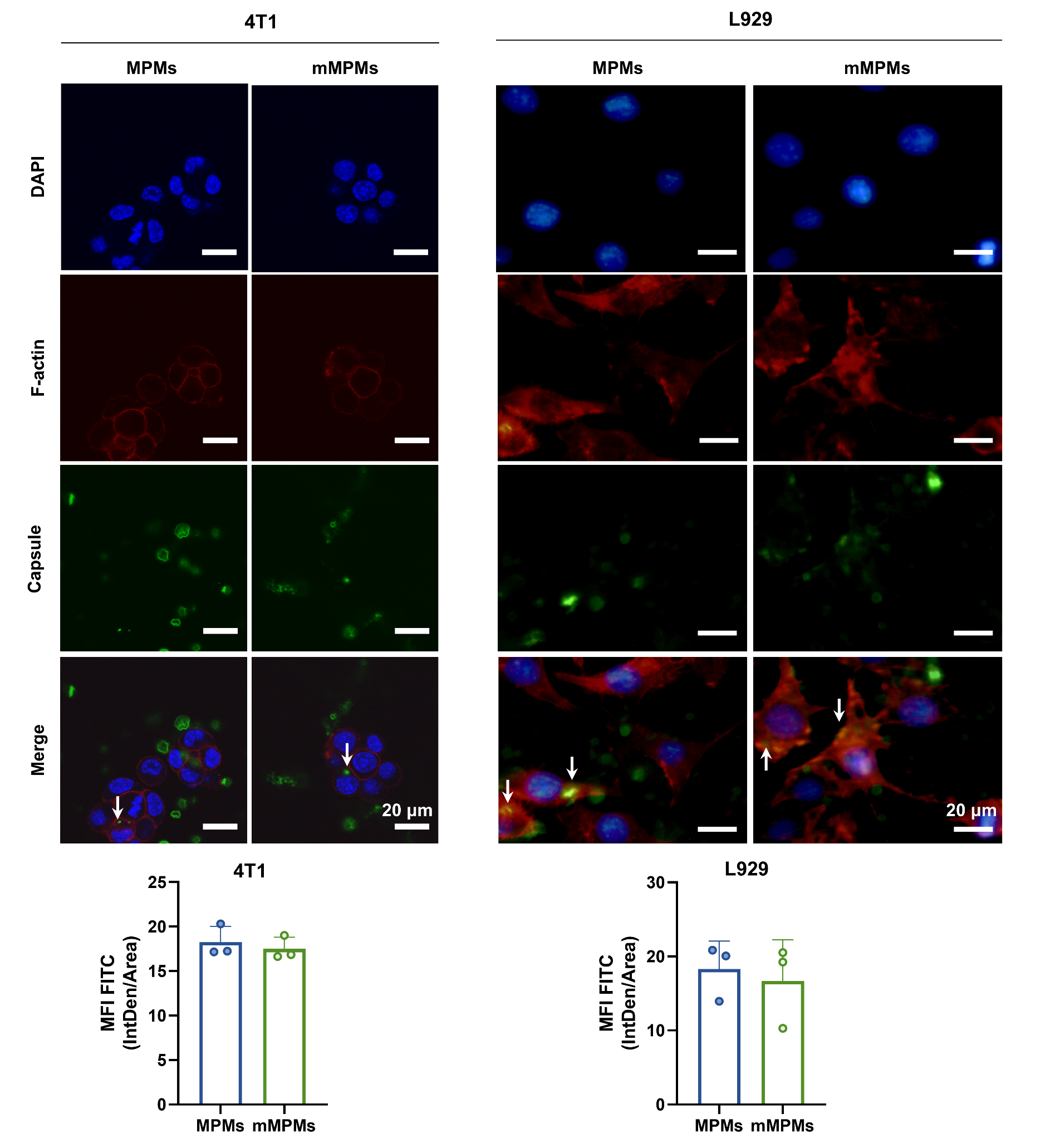


**Figure S12.** Confocal microscopy images and fluorescence microscopy images of 4T1 cells and L929 cells following exposure to MPMs or mMPMs for 2 h. Intracellular mean fluorescence intensity (MFI) derived from 3 independent experiments (n = 3 independent experimental units (EUs)). The microcapsules are stained with FITC (green), while cell nuclei and cytoskeleton are visualized in blue (DAPI) and red (Actin-Tracker Red), respectively. White arrows indicate the capsules internalized by the cells.


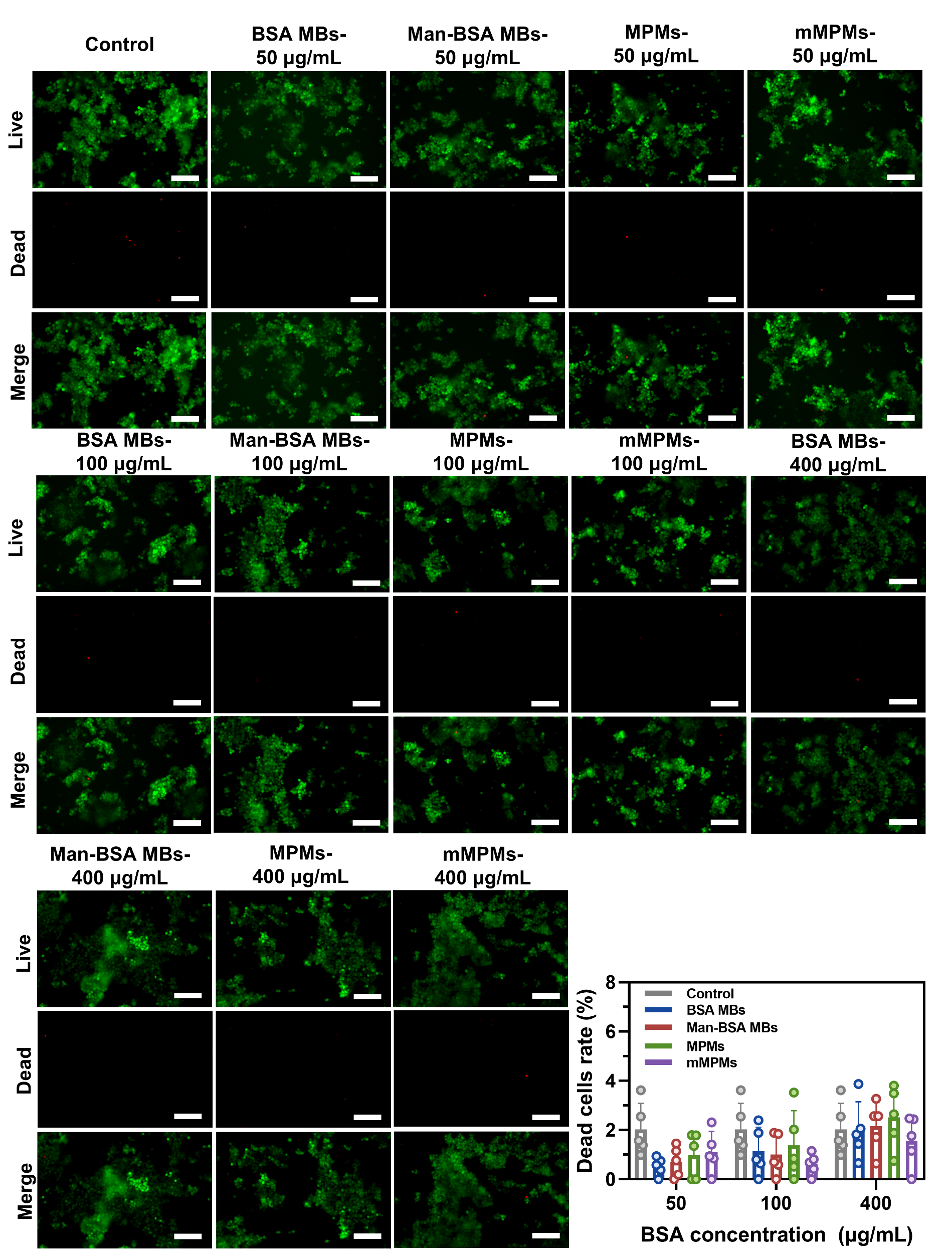


**Figure S13.** Live/dead staining and corresponding quantitative analysis of RAW 264.7 cells incubated with control, BSA MBs, Man-BSA MBs, MPMs and mMPMs for 24 h (n = 5 independent EUs). Scale bars are 100 μm.


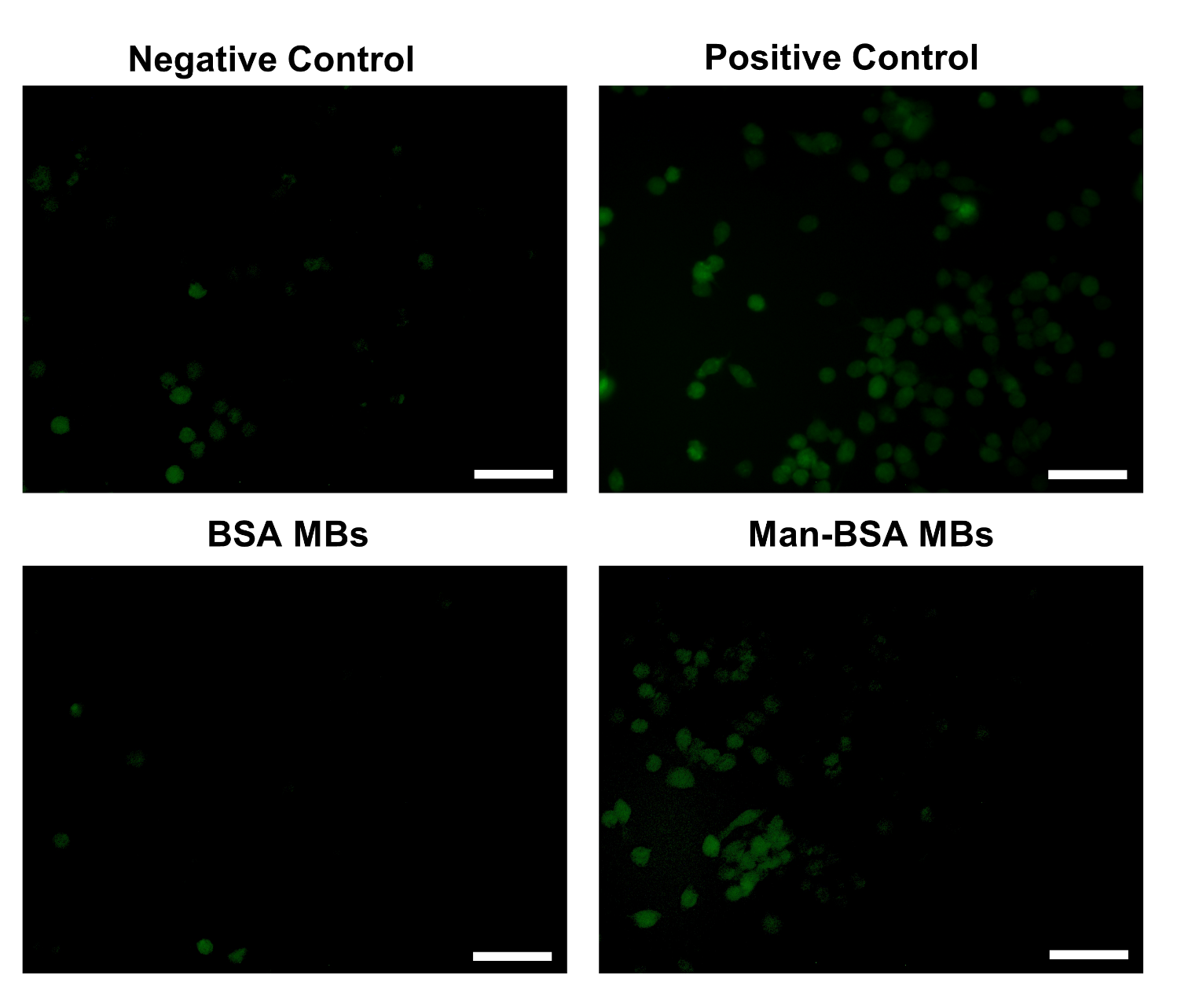


**Figure S14.** Representative images of ROS staining in macrophages treated with BSA MBs or Man-BSA MBs at the concentration of 200 μg/mL for 24 h. The untreated cell was set as a negative control, and the LPS-stimulated one was set as a positive control. Scale bars are 100 μm.


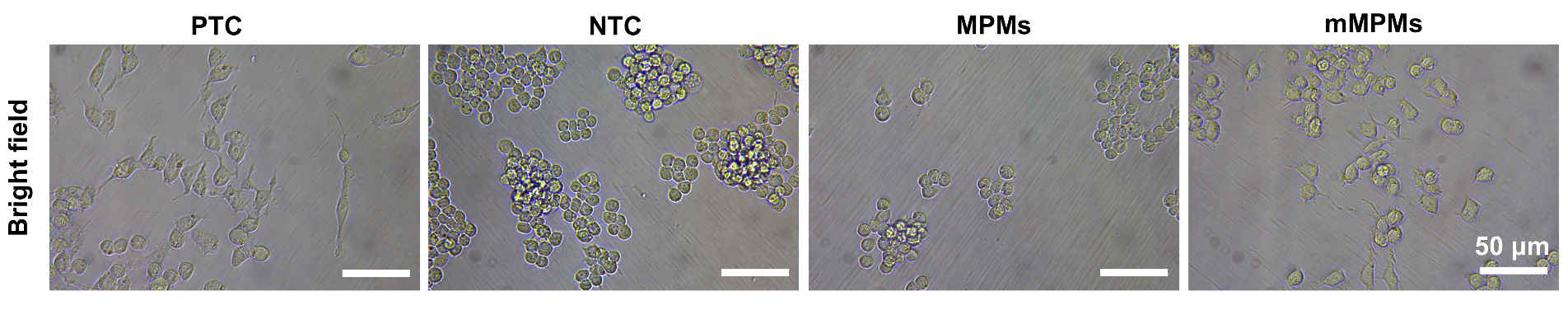


**Figure S15.** The optical images of RAW 264.7 cells treated with LPS, MPMs or mMPMs at the concentration of 200 μg/mL for 24 h. The untreated cell was set as a NTC.


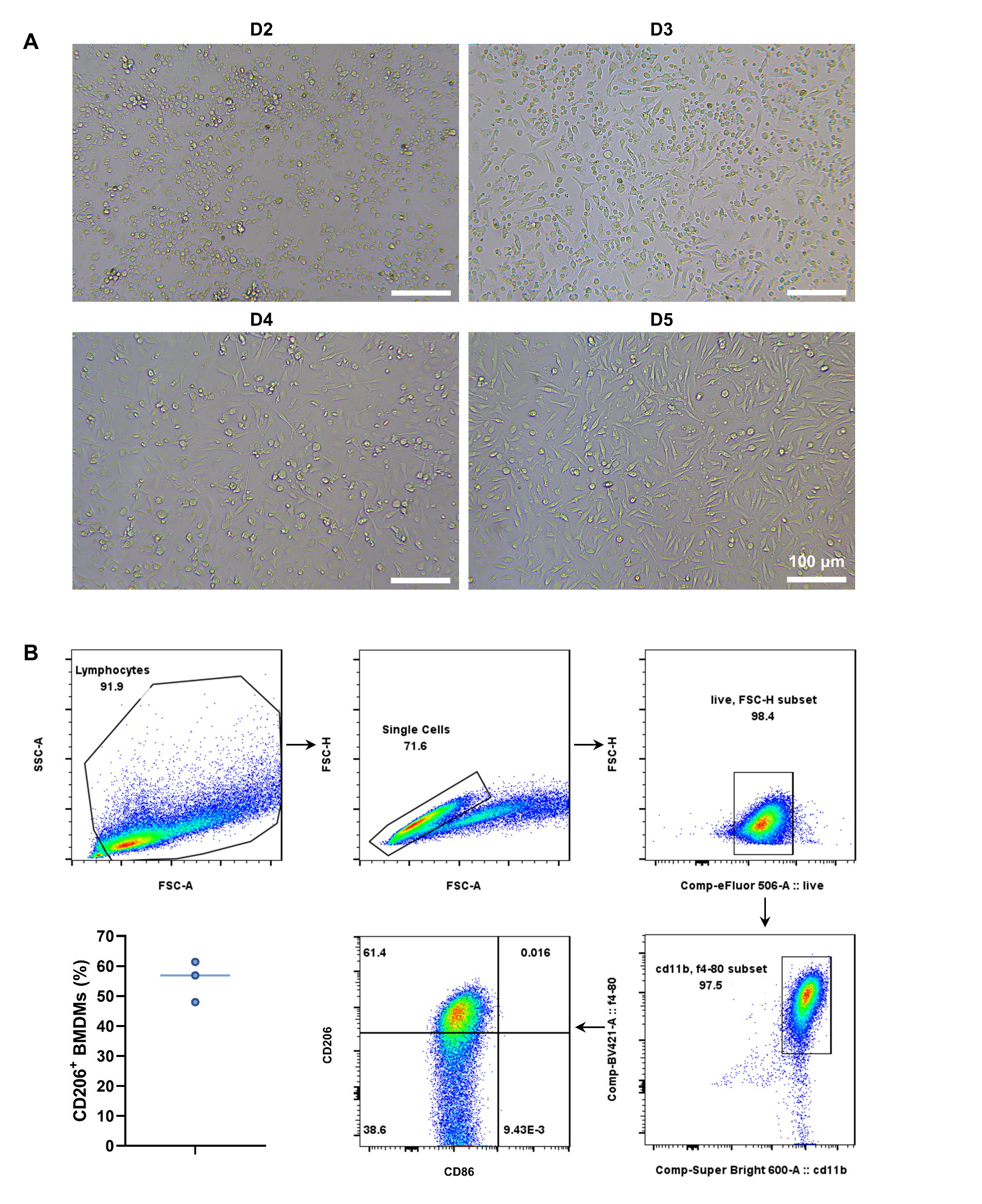


**Figure S16.** (A) Morphological changes of mouse bone marrow-derived cells incubated with 20% L929 cell supernatant for 2 to 5 days. (B) Flow cytometry analysis of cells harvested on day 5 and the corresponding proportion of CD206^+^ cells (n = 3 independent EUs).


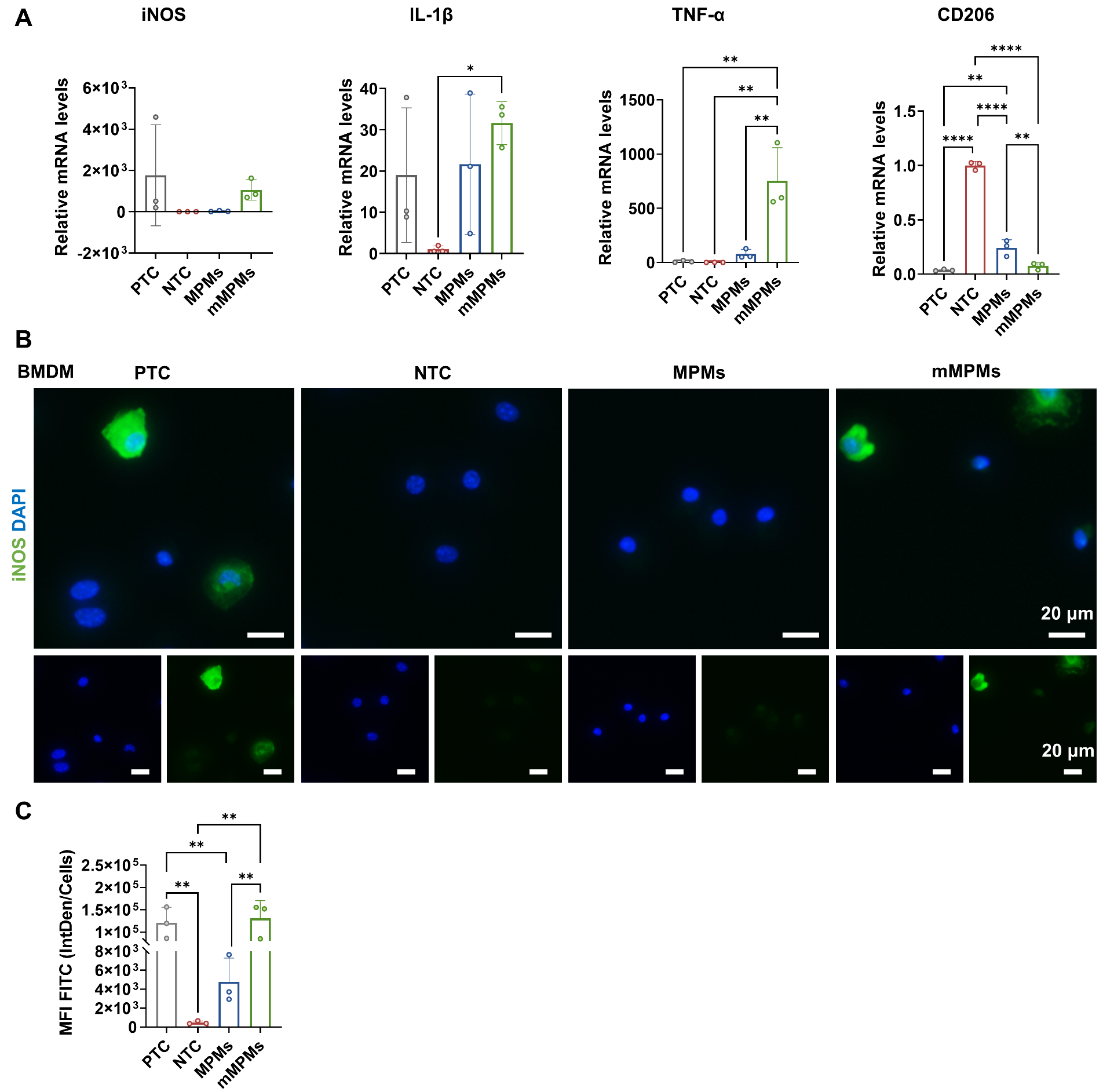


**Figure S17.** (A) Relative gene expressions of *iNOS*, *IL-1β*, *TNF-α*, and *CD206* in BMDMs treated with LPS (300 ng/mL), MPMs (200 μg/mL) or mMPMs (200 μg/mL) for 48 h (n = 3 independent EUs). (B) Representative images of immunofluorescence staining for iNOS in BMDMs after LPS, MPMs or mMPMs treatment for 48 h. Scale bars = 20 µm. (C) Quantitative analysis of immunofluorescence staining for iNOS (n = 3 independent EUs). * (p < 0.05); ** (p < 0.01); *** (p < 0.001); and **** (p < 0.0001) determined using one-way ANOVA with Tukey’s post hoc test (A and C).


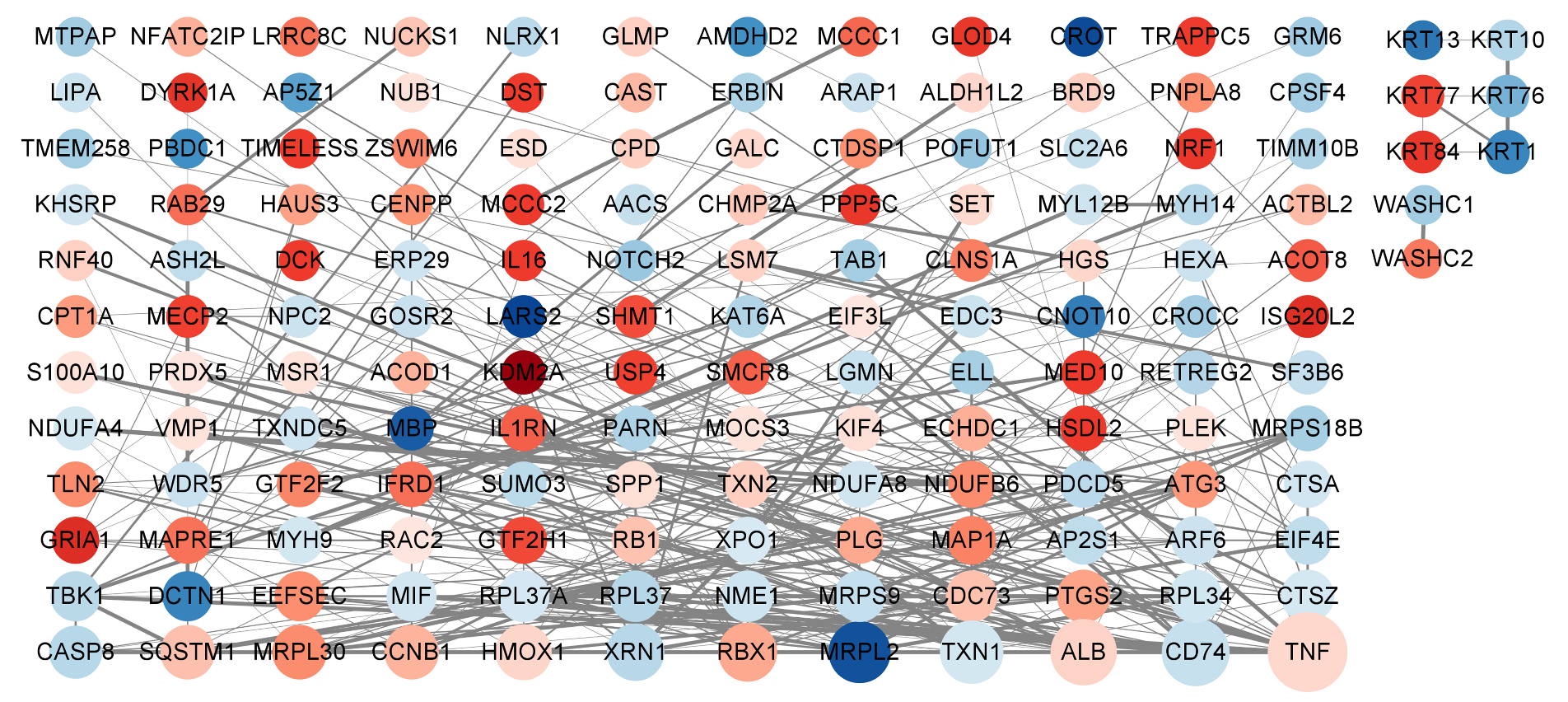


**Figure S18.** The protein interaction network for all DEPs (MPMs vs. mMPMs). In the protein network, a greater node size signifies a higher occurrence within the network. Up-regulated proteins are depicted in red, whereas down-regulated proteins are shown in blue. The intensity of node colors correlates with the degree of fold change in Differentially Expressed Proteins (DEPs).


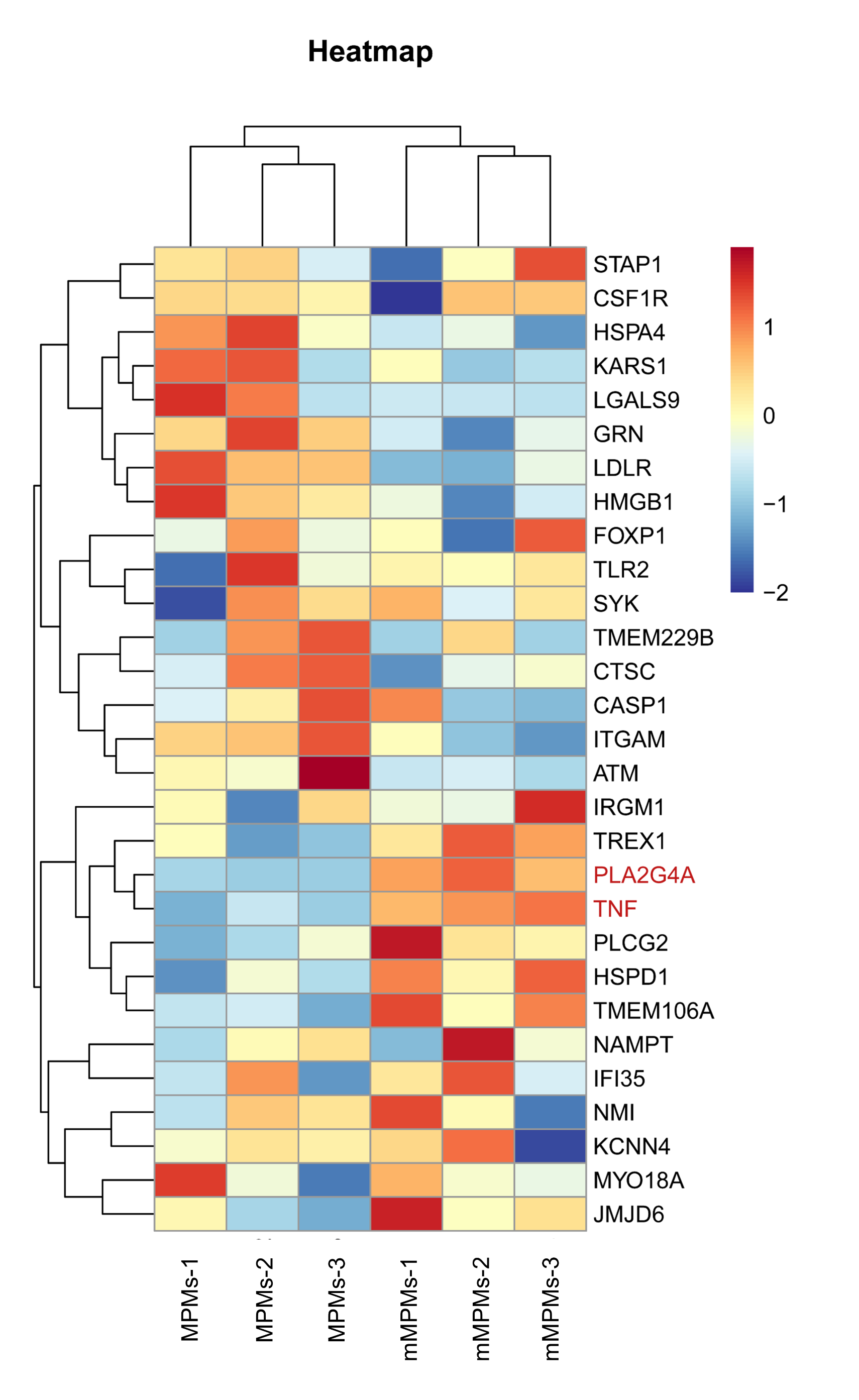


**Figure S19.** Protein expression of macrophage activation-related proteins from proteomic profiles (mMPMs vs. MPMs).


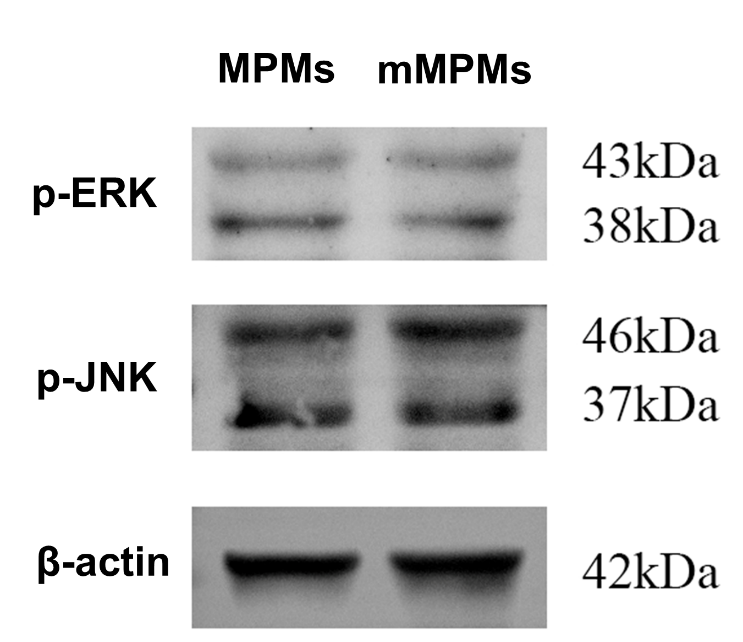


**Figure S20.** Immunoblot analyses of the specified proteins in the total cell lysate of RAW 264.7 cells following treatment with MPMs or mMPMs for 48 h.


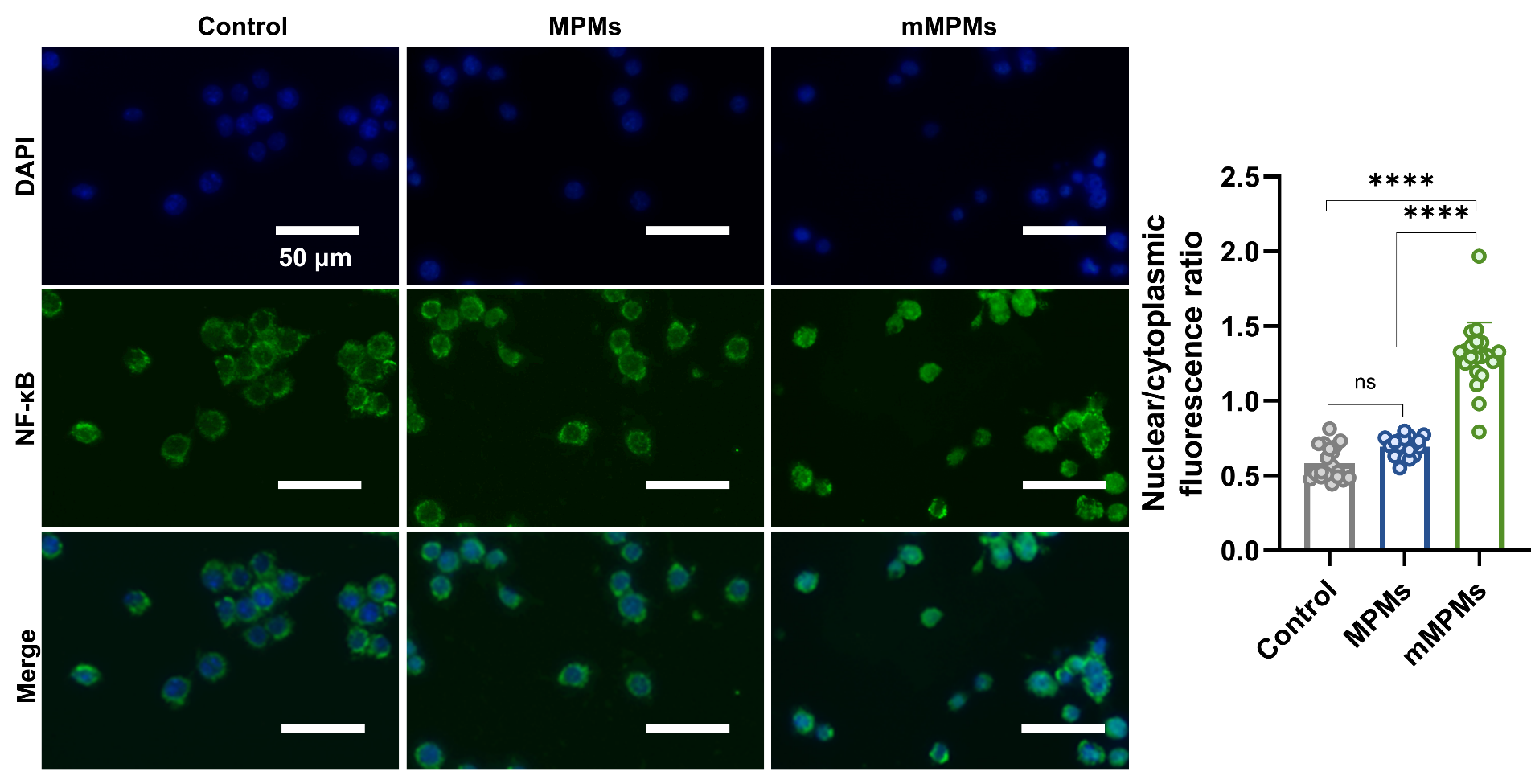


**Figure S21.** Illustrative immunofluorescence images displaying NF-κB staining in RAW 264.7 cells after MPMs or mMPMs exposure for 48 h. The untreated cells were set as control. Scale bars are 50 µm. The quantitative examination of NF-κB immunofluorescence (n = 20 cells from 3 independent experiments) was conducted, with results depicted as means ± SEM. Statistical analysis was performed using the Kruskal-Wallis test to determine P values. ****p < 0.0001.


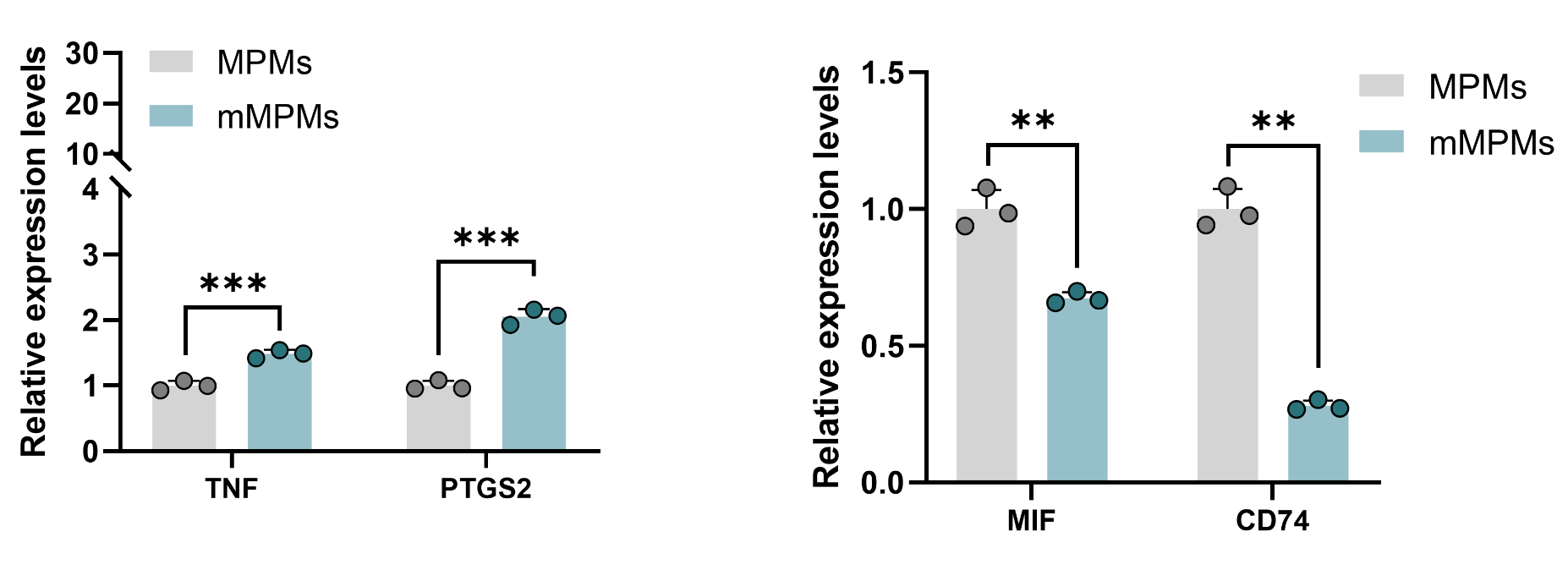


**Figure S22.** Protein expression of several inflammation-related mediators, macrophage migration inhibitory factor (MIF) and CD74 from proteomic profiles (n = 3 independent EUs).


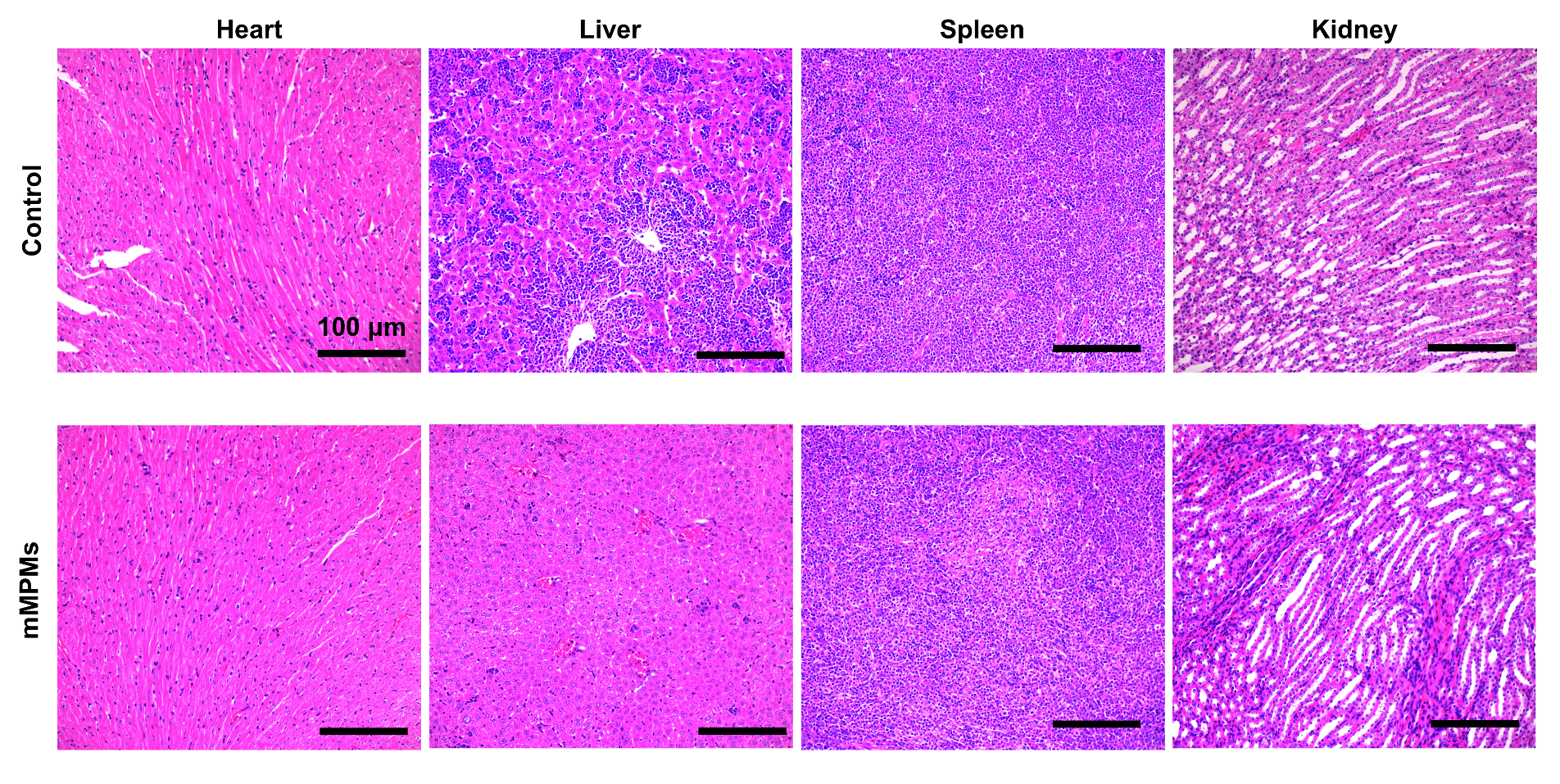


**Figure S23.** Histological sections of heart, liver, spleen, and kidney tissues from control and mMPMs groups were stained with hematoxylin and eosin (H&E). The images show representative sections from each group. Scale bars represent 100 μm.


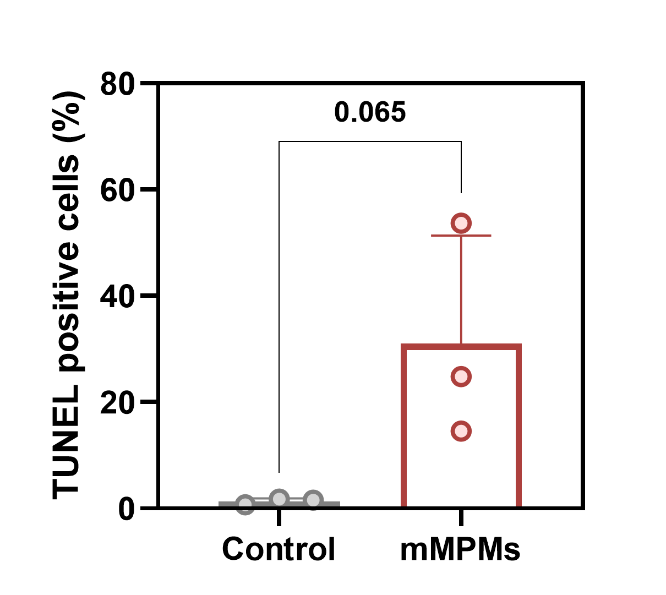


**Figure S24.** Quantification of TUNEL-positive cells from TUNEL staining images (n = 3 independent EUs).


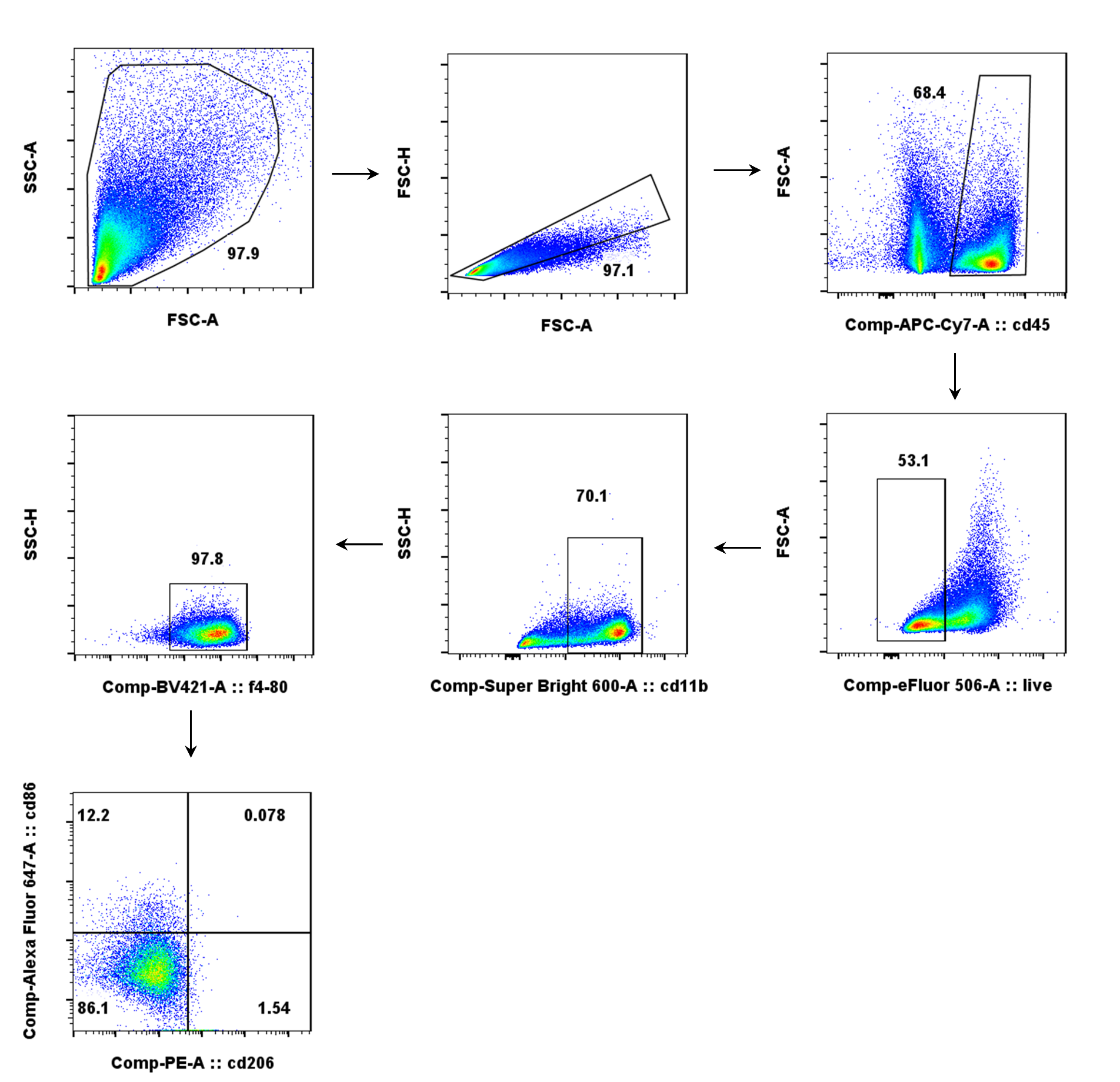


**Figure S25.** The flow cytometric gating strategy of M1 macrophages and M2 macrophages in tumors.

**Table S1.** The secondary structure percentages of BSA MBs, Man-BSA MBs at ratios of 1:2, 1:1, and 2:1.

| Wavenumber  (cm^-1^) | BSA MBs | Man-BSA MBs  (1:2) | Man-BSA MBs  (1:1) | Man-BSA MBs  (2:1) |
| --- | --- | --- | --- | --- |
| (1610-1620)  Intermolecular β-sheet | 21.72% | 14.59% | 5.91% | 15.29% |
| (1623-1636)  β, extended | 33.00% | 28.70% | 25.51% | 20.38% |
| (1642-1646)  Random coil | 10.12% | 13.62% | 4.94% | 0.13% |
| (1650-1655)  α-helix | 2.62% | 3.95% | 1.89% | 0% |
| (1656-1660)  α, unordered | 23.99% | 18.53% | 48.96% | 42.42% |
| (1662-1674)  turn | 3.64% | 9.43% | 2.59% | 4.04% |
| (1680-1690)  β-turn | 4.00% | 7.23% | 8.47% | 13.49% |

**Table S2.** Glycation sites identified in this work.

| Entry | Sequence | Modification | % site  Glycation |
| --- | --- | --- | --- |
| 1 | VPQVSTPTLVEVSR | 1×AGEs [Q3] | 88.35% |
| 2 | FWGKYLYEIAR | 1×AGEs [K/W] | 0.53% |
| 3 | YNGVFQECCQAEDK | 1×AGEs [N2] | 0.011% |
| 4 | LKPDPNTLCDEFKADEK | 1×AGEs [K17] | 0.06% |
| 5 | AEFVEVTKLVTDLTK | 1×Dou-mannose [K8] | 44.54% |
| 6 | AFDEKLFTFHADICTLPDTEK | 1×Dou-mannose [K5] | 9.38% |
| 7 | ATEEQLKTVMENFVAFVDK | 1×Dou-mannose [K7] | 2.99% |
| 8 | DAIPENLPPLTADFAEDKDVCKNYQEAK | 1×Dou-mannose [K18] | 0.76% |
| 9 | DDPHACYSTVFDKLKHLVDEPQNLIK | 1×Dou-mannose [K/H] | 3.81% |
| 10 | DDSPDLPKLKPDPNTLCDEFK | 1×Dou-mannose [K] | 17.92% |
| 11 | DDSPDLPKLKPDPNTLCDEFKADEK | 1×Dou-mannose [K8] | 21.40% |
| 12 | DLGEEHFKGLVLIAFSQYLQQCPFDEHVKLVNELTEFAK | 1×Dou-mannose [K/H] | 1.92% |
| 13 | FKDLGEEHFK | 1×Dou-mannose [K2] | 0.075% |
| 14 | FKDLGEEHFKGLVLIAFSQYLQQCPFDEHVK | 1×Dou-mannose [K/H] | 92.31% |
| 15 | GLVLIAFSQYLQQCPFDEHVKLVNELTEFAK | 1×Dou-mannose [K/H] | 91.63% |
| 16 | HLVDEPQNLIKQNCDQFEK | 1×Dou-mannose [K/Q] | 83.41% |
| 17 | LAKEYEATLEECCAK | 1×Dou-mannose [K3] | 4.49% |
| 18 | LFTFHADICTLPDTEKQIKK | 1×Dou-mannose [K/Q] | 90.47% |
| 19 | LFTFHADICTLPDTEKQIKK | 2×Dou-mannose [Q17; K20] | 49.97% |
| 20 | LGEYGFQNALIVR | 1×Dou-mannose [R13] | 62.08% |
| 21 | LKPDPNTLCDEFKADEKK | 1×Dou-mannose [K13] | 41.39% |
| 22 | QNCDQFEKLGEYGFQNALIVR | 1×Dou-mannose [K/Q] | 3.53% |
| 23 | RPCFSALTPDETYVPKAFDEK | 1×Dou-mannose [K16] | 9.25% |
| 24 | SHCIAEVEKDAIPENLPPLTADFAEDKDVCK | 1×Dou-mannose [N15] | 0.17% |
| 25 | SLHTLFGDELCKVASLR | 1×Dou-mannose [K12] | 11.46% |
| 26 | TCVADESHAGCEKSLHTLFGDELCK | 1×Dou-mannose [K/H] | 1.26%` |
| 27 | TCVADESHAGCEKSLHTLFGDELCKVASLR | 1×Dou-mannose [K/H] | 9.39% |
| 28 | TVMENFVAFVDKCCAADDKEACFAVEGPK | 1×Dou-mannose [K12] | 30.93% |
| 29 | AEFVEVTKLVTDLTK | 1×Mannose [K8] | 88.60% |
| 30 | AFDEKLFTFHADICTLPDTEK | 1×Mannose [H/K] | 7.13% |
| 31 | ATEEQLKTVMENFVAFVDK | 1×Mannose [Q/K/N] | 80.42% |
| 32 | CCAADDKEACFAVEGPK | 1×Mannose [K7] | 67.69% |
| 33 | DDPHACYSTVFDKLKHLVDEPQNLIK | 1×Mannose [K] | 57.44% |
| 34 | DLGEEHFKGLVLIAFSQYLQQCPFDEHVK | 1×Mannose [K8] | 93.22% |
| 35 | ECCHGDLLECADDR | 1×Mannose [H4] | 0.64% |
| 36 | EYEATLEECCAKDDPHACYSTVFDK | 1×Mannose [K/H] | 85.27% |
| 37 | FKDLGEEHFK | 1×Mannose [K/H] | 9.22% |
| 38 | FKDLGEEHFKGLVLIAFSQYLQQCPFDEHVK | 1×Mannose [Q/K/H] | 80.35% |
| 39 | GLVLIAFSQYLQQCPFDEHVKLVNELTEFAK | 1×Mannose [H/K/Q/N] | 44.08% |
| 40 | HLVDEPQNLIKQNCDQFEK | 1×Mannose [K11] | 63.76% |
| 41 | HLVDEPQNLIKQNCDQFEK | 2×Mannose [Q12; Q16] | 14.95% |
| 42 | HPYFYAPELLYYANK | 2×Mannose [N14; K15] | 94.88% |
| 43 | HPYFYAPELLYYANKYNGVFQECCQAEDK | 1×Mannose [Q/N/K] | 39.18% |
| 44 | KQTALVELLK | 1×Mannose [Q2] | 0.38% |
| 45 | KVPQVSTPTLVEVSR | 1×Mannose [K/Q] | 0.046% |
| 46 | LFTFHADICTLPDTEK | 1×Mannose [H5] | 0.0061% |
| 45 | LFTFHADICTLPDTEKQIK | 1×Mannose [K16] | 83.77% |
| 46 | LGEYGFQNALIVR | 1×Mannose [R/Q/N] | 0.038% |
| 47 | LGEYGFQNALIVR | 2×Mannose [Q7; R13] | 55.9% |
| 48 | LKECCDKPLLEK | 1×Mannose [K2] | 11.09% |
| 49 | LKHLVDEPQNLIK | 1×Mannose [K/H] | 78.98% |
| 50 | LKPDPNTLCDEFK | 1×Mannose [N/K] | 0.049% |
| 51 | LKPDPNTLCDEFKADEK | 1×Mannose [K2] | 68.14% |
| 52 | LSQKFPKAEFVEVTK | 1×Mannose [K/Q] | 92.60% |
| 53 | LVNELTEFAKTCVADESHAGCEKSLHTLFGDELCK | 2×Mannose [K10; K23] | 11.30% |
| 54 | MPCTEDYLSLILNRLCVLHEK | 1×Mannose [R/N] | 0.0043% |
| 55 | NYQEAKDAFLGSFLYEYSR | 2×Mannose [N1; K/Q] | 2.65% |
| 56 | QNCDQFEKLGEYGFQNALIVR | 2×Mannose [Q/N/K] | 48.35% |
| 57 | RHPYFYAPELLYYANKYNGVFQECCQAEDK | 1×Mannose [N/K/Q] | 79.99% |
| 58 | RPCFSALTPDETYVPKAFDEK | 1×Mannose [K16] | 53.15% |
| 59 | SHCIAEVEKDAIPENLPPLTADFAEDK | 1×Mannose [K/N] | 74.85% |
| 60 | SHCIAEVEKDAIPENLPPLTADFAEDKDVCK | 1×Mannose [K/N] | 0.25% |
| 61 | SHCIAEVEKDAIPENLPPLTADFAEDKDVCK | 2×Mannose [K9; K27] | 82.77266364 |
| 62 | SLHTLFGDELCKVASLR | 1×Mannose [K12] | 55.32% |
| 63 | TCVADESHAGCEKSLHTLFGDELCK | 2×Mannose [H16; H/K] | 51.75% |
| 64 | TVMENFVAFVDKCCAADDK | 1×Mannose [K12] | 56.44% |
| 65 | TVMENFVAFVDKCCAADDKEACFAVEGPK | 1×Mannose [K/N] | 84.13% |

**Table S3.** The percentages of secondary structures of MPMs and mMPMs.

| Wavenumber  (cm^-1^) | MPMs | mMPMs |
| --- | --- | --- |
| (1610-1620)  Intermolecular β-sheet | 1.40% | 10.97% |
| (1623-1636)  β, extended | 41.21% | 30.92% |
| (1642-1646)  Random coil | 12.65% | 12.52% |
| (1650-1655)  α-helix | 4.32% | 3.99% |
| (1656-1660)  α, unordered | 20.26% | 16.47% |
| (1662-1674)  turn | 7.43% | 12.95% |
| (1680-1690)  β-turn | 8.82% | 10.37% |

**Table S4.** Primer sequences are specific to RAW 264.7 for qPCR.

| Species | Gene | Forward & Reverse | Sequence (5’- 3’) |
| --- | --- | --- | --- |
| Mouse | *GAPDH* | Forward | GCAAGTTCAACGGCACAG |
|  |  | Reverse | CGCCAGTAGACTCCACGAC |
| Mouse | *CCR7* | Forward | ATGGACCCAGGTGTGCTTCT |
|  |  | Reverse | TCAGTATCACCAGCCCGTTG |
| Mouse | *IL-10* | Forward | CCAAGCCTTATCGGAAATGA |
|  |  | Reverse | TTTTCACAGGGGAGAAATCG |
| Mouse | *CD206* | Forward | AGCTTCATCTTCGGGCCTTTG |
|  |  | Reverse | GGTGACCACTCCTGCTGCTTTAG |
| Mouse | *iNOS* | Forward | AAGGAGAACCAAGCAACGACAAAA |
|  |  | Reverse | TGGGGAACTCTGCAGACTCAAACT |
| Mouse | *ARG-1* | Forward | GTGAAGAACCCACGGTCTGT |
|  |  | Reverse | CTGGTTGTCAGGGGAGTGTT |
| Mouse | *TNF-α* | Forward | CGTCAGCCGATTTGCTATCT |
|  |  | Reverse | CGGACTCCGCAAAGTCTAAG |
| Mouse | *IL-1β* | Forward | AAGGAGAACCAAGCAACGACAAAA |
|  |  | Reverse | TGGGGAACTCTGCAGACTCAAACT |
